# Integrated Scaffold Redesign and iPSC-Based Screening Reveal Potent Antifibrotic Artemisinin Analogs in Systemic Sclerosis Models

**DOI:** 10.1021/acscentsci.6c00658

**Published:** 2026-09-12

**Authors:** Takehiro Ishiga, Tetsuya Ikawa, Nobuto Kaneko, Norihito Takahashi, Krishanu Mondal, Yasuhiro Nakano, Yutaro Hori, Atsushi Miyajima, Taketomo Kido, Yoshihide Asano, Hiroki Oguri

**Affiliations:** † Department of Chemistry, Graduate School of Science, 13143 The University of Tokyo, 7-3-1 Hongo, Bunkyo-ku, Tokyo 113-0033, Japan; ‡ Department of Dermatology, Graduate School of Medicine, 38047 Tohoku University, 1-1 Seiryo-machi, Aoba-ku, Sendai 980-8574, Japan; § Department of Applied Chemistry, Graduate School of Engineering, 98293 Tokyo University of Agriculture and Technology, 2-24-16, Naka-cho, Koganei, Tokyo 184-8588, Japan; ∥ Integrated Systems of Aging Research Unit, Institute for Frontier Science Initiative, Kanazawa University, Kakuma-machi, Kanazawa, Ishikawa 920-1192, Japan; ⊥ Laboratory of Genome Regeneration, Institute for Quantitative Biosciences, 13143 The University of Tokyo, 1-1-1 Yayoi, Bunkyo-ku, Tokyo 113-0032, Japan; # Laboratory of Cell Growth and Differentiation, Institute for Quantitative Biosciences, 13143 The University of Tokyo, 1-1-1 Yayoi, Bunkyo-ku, Tokyo 113-0032, Japan

## Abstract

Fibrotic diseases remain among the most intractable human disorders, largely due to the absence of therapies capable of directly modulating the core cellular programs that drive pathological matrix deposition and tissue remodeling. To address this unmet medical need, we report an integrated, chemistry-driven discovery platform for function-oriented molecular design and discovery that combines scaffold redesign of a classical natural product pharmacophore with human induced pluripotent stem cell (iPSC)-based phenotypic screening to identify potent antifibrotic agents. Systematic modification of the artemisinin scaffold led to the identification of 6-aza-artemisinins with markedly enhanced antifibrotic activity, including an N6–N6′ dimeric analog exhibiting high potency at sub-micromolar concentrations. These compounds suppressed collagen production in systemic sclerosis patient-derived fibroblasts and ameliorated fibrosis in a bleomycin-induced murine model, with superior efficacy relative to the clinically used antimalarial drug artesunate. Notably, efficacy was observed even when treatment was initiated after fibrosis establishment. Transcriptomic analysis revealed coordinated suppression of core fibrotic and inflammatory pathways, providing mechanistic insight into the observed therapeutic effects. Collectively, these findings establish 6-aza-artemisinins as a new chemotype for antifibrotic intervention and illustrate how scaffold-level redesign of natural products, integrated with disease-relevant stem-cell-based models, can enable next-generation function-driven therapeutic discovery.

## Graphical abstract

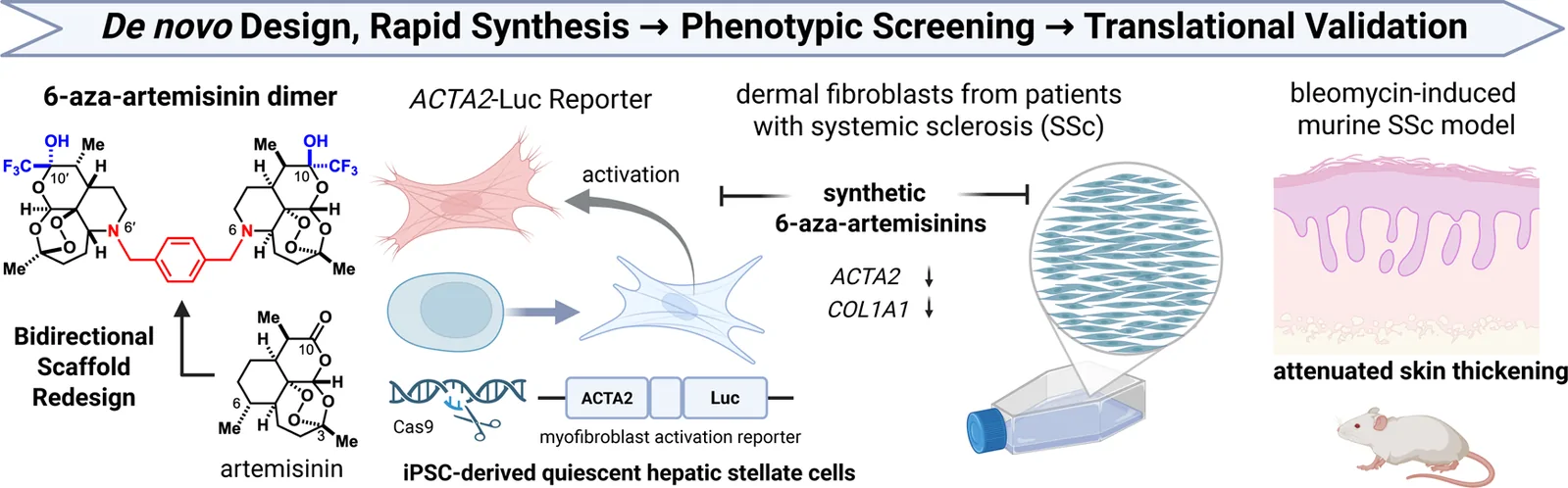

## Introduction

Fibrosis is a defining pathological feature of numerous chronic diseases and a major contributor to organ failure and mortality.^1^ Regardless of the affected organ—whether liver, lung, skin, or kidney—the fibrotic process is orchestrated by the persistent activation of fibroblasts into extracellular matrix-producing myofibroblasts, driven by sustained exposure to inflammatory and profibrotic cues such as transforming growth factor-β (TGF-β).^2,3^ Despite its central role in disease progression, effective antifibrotic therapies remain elusive, underscoring the need for new strategies that directly modulate fibrotic pathways beyond general immunosuppression.

Systemic sclerosis (SSc) is a progressive autoimmune disorder marked by microvascular injury and widespread fibrosis affecting both the skin and internal organs.^4,5^ Among its complications, interstitial lung disease (ILD) is particularly prevalent and represents the leading cause of SSc-related mortality. Although current treatment options—including immunosuppressive agents and antifibrotic drugs such as nintedanib^6^ —may decelerate disease progression, their ability to ameliorate established fibrosis remains limited.^4,5,7^ Consequently, there is an urgent need for novel therapeutics that can directly target fibrotic mechanisms without relying on broad immunosuppression.

To overcome the longstanding challenge of limited access to primary human hepatic stellate cells (HSCs) for antifibrotic drug discovery, we developed an innovative *in vitro* platform utilizing quiescent HSCs differentiated from human induced pluripotent stem cells (iPSCs) harboring an *ACTA2*–red fluorescent protein (RFP) reporter construct.^8,9^ This system enables real-time and quantitative monitoring of myofibroblast activation—a central pathogenic event in liver fibrosis—through fluorescent tracking of α-smooth muscle actin (α-SMA) expression. Leveraging this physiologically relevant model, we performed a high-throughput screen of approximately 1,500 clinically approved compounds and identified the antimalarial natural product artemisinin^10^ as a top-ranking hit that robustly inhibited HSC activation.^11^

Building on this foundation, we established a next-generation reporter platform in which the RFP fluorescent readout was replaced with Firefly luciferase (*ACTA2*-Luc) (Figure 1A). Leveraging recent advances in luciferase-based assay technologies,^12^ this modification markedly improved sensitivity, signal-to-noise ratio, and dynamic range while preserving the physiological relevance of the iPSC-derived quiescent HSC model. The enhanced assay allowed for more quantitative and high-throughput-compatible evaluation of candidate antifibrotic agents (see Results).

**Figure 1:**
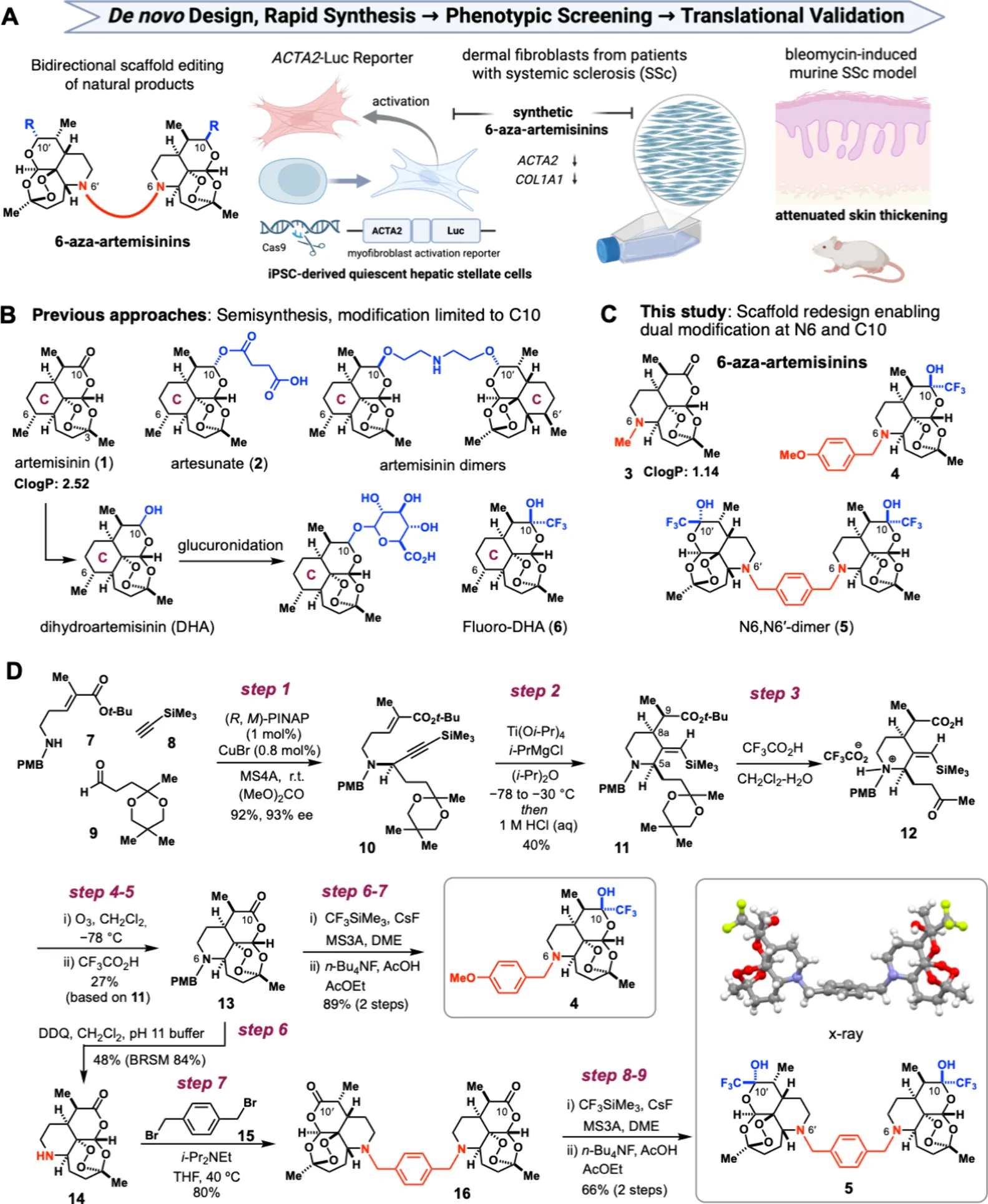
**Integrated discovery platform and** ***de novo*** **synthesis of 6-aza-artemisinins**. (A) Schematic overview of the study workflow linking *de novo* synthesis, iPSC-based screening, and *in vivo* evaluation. (B) Metabolic transformations of artemisinin (**1**) and representative semisynthetic antimalarials such as artesunate (**2**). (C) Fully synthetic 6-aza-artemisinins developed in this study feature bidirectional tunability at both N6 and C10 positions, enabling scaffold-level diversification beyond naturally derived analogs. (D) Modular asymmetric *de novo* synthesis affording the 6-aza-artemisinins monomer **4** and dimer **5** in seven and nine steps overall, respectively.

Although the identification of artemisinin as an antifibrotic agent marked a promising step toward drug repurposing for liver fibrosis, its direct therapeutic application is severely hampered by poor aqueous solubility and limited opportunities for chemical derivatization.^13,14^ These challenges have hindered the development of artemisinin-based or inspired antifibrotic agents.

Recent advances in synthetic biology have enabled the large-scale production of artemisinic acid—a biosynthetic precursor of artemisinin—which can be converted into the natural product artemisinin (**1**) through a four-step semisynthetic process, facilitating its commercial availability (Figure 1B).^15^ However, nearly all clinically used derivatives, exemplified by the widely adopted artesunate, rely on modifications at the C10 position to improve pharmacokinetic properties such as solubility and metabolic stability.^13,14^ While these semisynthetic analogs have revolutionized malaria treatment, the complex peroxide-bridged tetracyclic core of artemisinin remains largely unmodified. Consequently, the therapeutic potential of scaffold-level redesign beyond C10 substitution remains untapped, highlighting the urgent need for a synthetically tractable and rationally modifiable scaffold capable of retaining or even enhancing the antifibrotic activity of the widely used antimalarial agent.

To address these challenges, we designed and developed 6-aza-artemisinins—a novel class of synthetic analogs in which the C6 carbon of the artemisinin tetracyclic core is replaced with a nitrogen atom (Figure 1C).^16–19^ The ″6-aza-artemisinin″ platform provides a strategic entry point for diversification. Replacement of the C6 carbon with a nitrogen atom unlocks a chemically addressable site for molecular derivatization that is inaccessible in the natural product, while enabling modulation of the physicochemical and pharmacological properties of the tetracyclic scaffold. This key innovation allowed us to construct the N6–N6′-linked dimer **5**, a topology previously unexplored in artemisinin medicinal chemistry. By accessing this new chemical space, we overcame the limitations of C10-only modifications, leading to the discovery of analogs with fundamentally improved biological activity and metabolic stability.

In this study, we report the rational design, synthesis, and preclinical validation of 6-aza-artemisinins as a structurally and mechanistically innovative class of antifibrotic agents (Figure 1A). These fully synthetic small molecules display potent and broad-spectrum efficacy in both hepatic and dermal fibrotic models, suppressing key profibrotic markers such as *ACTA2* and *COL1A1* in iPSC-derived HSCs and dermal fibroblasts from patients with systemic sclerosis (SSc). The lead analogs **4** and **5** demonstrated strong therapeutic benefit in bleomycin-induced murine models, significantly reducing dermal fibrosis under both prophylactic and therapeutic conditions. By overcoming the intrinsic pharmacological and chemical limitations of natural artemisinins, this new scaffold establishes a synthetically accessible and modular platform for mechanism-guided antifibrotic drug discovery, bridging iPSC-based phenotypic screening with *in vivo* therapeutic validation (Figure 1A).

## Results

### Design of 6-Aza-artemisinins

In our previous high-throughput phenotypic screen of 1,453 clinically relevant compounds, artemisinin was identified as a potent inhibitor of myofibroblast activation using human iPSC-derived quiescent hepatic stellate cells (qHSCs) engineered with an *ACTA2*-RFP reporter.^11^ Artemisinin (**1**), a sesquiterpene lactone featuring a distinctive endoperoxide bridge, has long served as a cornerstone of antimalarial therapy.^10,13^ Although a concise total synthesis of (+)-artemisinin (**1**) has been reported,^20^ its current production predominantly relies on a semisynthetic route that begins with the fermentation-based biosynthesis of artemisinic acid using genetically engineered *Saccharomyces cerevisiae*, followed by a four-step chemical transformation to afford **1**.^15^ This method allows for large-scale production and has further enabled the development of clinically useful C10-modified derivatives, such as artesunate (**2**), through an additional two-step semisynthetic transformation. Despite these advancements, structural modifications have thus far been largely limited to the C10 position.^13,14^

To address these challenges, we pursued a strategic redesign of the artemisinin core scaffold to enable a *de novo* catalytic asymmetric synthesis, while expanding opportunities for structural diversification. As reported previously, we established a synthetic route to 6-aza-artemisinin analogs (**3**) as a fully synthetic artemisinin-inspired scaffold in which the C6 stereogenic center of **1** is replaced by a nitrogen atom.^16–19^ Building on this fully synthetic platform, we sought to investigate whether systematic scaffold diversification could uncover derivatives with enhanced antifibrotic activity (Figures 1C and D). This elemental substitution allows for a modular, step-economical, and catalytic asymmetric construction of the tetracyclic core, while simultaneously introducing a synthetically versatile heteroatom handle at the previously inaccessible C6 position for late-stage diversification.

### *De Novo* Synthesis of the 6-Aza-artemisinin and C10 Trifluoromethylation to Improve Metabolic Stability

Building upon our previously established catalytic asymmetric synthesis,^16–19^ the key tetracyclic intermediate **13**, bearing both an endoperoxide bridge and a *p*-methoxylbenzylated (PMB) amino group at the 6 position, was accessed in just five steps with modification to the late-stage transformations (**11**→**12**→**13**) (Figure 1D). Artemisinins are metabolically converted to dihydroartemisinin (DHA) via reduction of the C10 lactone, and DHA is believed to be primarily responsible for their antimalarial activity. However, DHA is rapidly glucuronidated at its C10 hydroxyl group, resulting in poor metabolic stability and a short half-life.^21^ To overcome this limitation by suppressing glucuronidation at the C10 position, Delpon and colleagues reported that trifluoromethylation of the C10 lactone using CF_3_SiMe_3_ (Ruppert–Prakash reagent) to afford a hemiacetal derivative, fluoro-DHA (**6**).^22^ The electron-deficient and sterically demanding nature of the trifluoromethyl group renders the fluoro-DHA resistant to glucuronidation. Inspired by this strategy, we performed C10 trifluoromethylation on 6-aza-artemisinin **13**. A modified protocol was developed involving activation of the Ruppert–Prakash reagent with CsF in THF, followed by treatment with tetrabutylammonium fluoride (TBAF) in the presence of acetic acid. This sequence afforded the desired trifluoromethylated 6-aza-artemisinin **4** with excellent diastereocontrol, and its structure was unambiguously confirmed by single-crystal X-ray analysis (Figure S1). As previously observed in the conversion of artemisinin (**1**) to its corresponding trifluoromethylated hemiacetal, the initial trifluoromethylation proceeds from the β-face, and subsequent desilylation induces almost-complete inversion at the cyclic hemiacetal center.^22^ This stereocontrolled process ultimately furnishes compound **4** with high diastereoselectivity. Collectively, this modular synthetic platform allows precise control over three-dimensional connectivity and bidirectional structural modification, establishing a new dimension of diversity in artemisinin-based pharmacophores.

### Generation of a N6–N6′-Linked Dimeric Aza-artemisinins

Dimeric natural products often exhibit enhanced biological functions, such as improved potency and selectivity, due to their conformationally constrained and spatially organized architectures that facilitate multivalent interactions with molecular targets.^23,24^ Building on this principle, several dimeric artemisinin derivatives have been reported to exhibit superior antimalarial and anticancer activities compared to their monomeric counterparts.^25–27^ These studies have primarily focused on installing linkers at the C10 lactone position, yielding C10–C10′ linked dimers (Figure 1B). It has been suggested that both the stereochemistry of the C10 hemiacetal center and the nature of the linker play crucial roles in modulating biological activity. However, to date, all reported dimerization strategies have relied exclusively on C10-based conjugation. No studies have explored the C-ring substructure as a dimerization site, and the dimerization through the 6 position—accessibly only through scaffold-level redesign—remains entirely unexplored. Importantly, while the antifibrotic potential of artemisinin-type compounds is gaining increasing attention, there are, to our knowledge, no reports evaluating the antifibrotic activity of dimeric artemisinin derivatives.

To address these gaps, we designed and synthesized a structurally unique N6, N6′-dimeric analog (**5**) by leveraging the modularity and bidirectional tunability of the 6-aza-artemisinin scaffold (Figures 1A and 1C). This dimer was constructed via N6, N6′ linkage through the aliphatic amino groups embedded in the C-ring of each monomer, enabling a conjugation strategy that is synthetically inaccessible from naturally occurring artemisinins. Removal of the *p*-methoxybenzyl (PMB) protecting group at the N6 position was achieved under oxidative conditions using DDQ (2,3-dichloro-5,6-dicyano-*p*-benzoquinone) in a biphasic system comprising pH11 phosphate buffer and CH_2_Cl_2_, affording the corresponding secondary amine **14** (Figure 1D). Subsequent alkylation of **14** with 1,4-bis­(bromomethyl)­benzene **15** proceeded smoothly at 40 °C to afford the N6, N6′-linked dimer **16** in 80% yield. Trifluoromethylation at the C10 and C10′ positions was then performed using our modified protocol: activation of CF_3_SiMe_3_ (Ruppert–Prakash reagent) with CsF in THF, followed by silyl group removal with TBAF in the presence of acetic acid.^22^ This sequence furnished the targeted N6, N6′-dimeric 6-aza-artemisinin **5** in 66% for two steps, and single-crystal X-ray analysis confirmed its *C*_2_-symmetric dimeric structure (Figure S2).

### *In Vitro* Antifibrotic Evaluation Using ACTA2-Luc Reporter iPSC-Derived qHSCs

To build on previously established iPSC-based reporter systems for monitoring myofibroblast activation in hepatic stellate cells (HSCs)^11^—in which the *ACTA2* promoter drives a red fluorescent protein (RFP) reporter, we developed a more sensitive and quantitative platform by replacing the fluorescent reporter with Firefly luciferase (Figures 1A, S3A–C). The resulting human iPSC line stably harboring an *ACTA2*–luciferase (*ACTA2*-Luc) cassette enabled real-time and highly sensitive detection of *ACTA2* promoter activity via luminescence readout. Compared with the RFP-based system, the luciferase assay exhibited greater sensitivity and throughput compatibility, providing an optimized platform for the quantitative evaluation of 6-aza-artemisinin derivatives under standardized assay conditions (2 μM, 72 h). Following identification of artemisinin in the initial RFP reporter-based screen, this ACTA2-luciferase platform was subsequently used for the evaluation and optimization of newly synthesized 6-aza-artemisinin derivatives.

### Structure–Activity Relationships (SAR) at the N6 Substituent

We next explored the preliminary structure–activity relationship (SAR) focusing on substitution at the N6 nitrogen atom through antifibrotic evaluation using the *ACTA2*-Luc reporter iPSC-derived qHSC assay (Figure 2A). Compared with artemisinin (**1**), artesunate (**2**), and 6-aza-artemisinin (**3**)—all bearing a methyl substituent at 6 positions—6-aza-artemisinins **13**, **17**, and **18** bearing benzyl-type substituents generally exhibited stronger suppression of *ACTA2* expression. Notably, the para-substituted benzyl derivatives **13** and **18** showed greater suppression than **17**. Considering both potency and synthetic accessibility, **13** emerged as the optimal monomeric scaffold. Although some variation was within the range of experimental variability, the overall SAR trend provided the foundation for subsequent structure-guided optimization.

**Figure 2:**
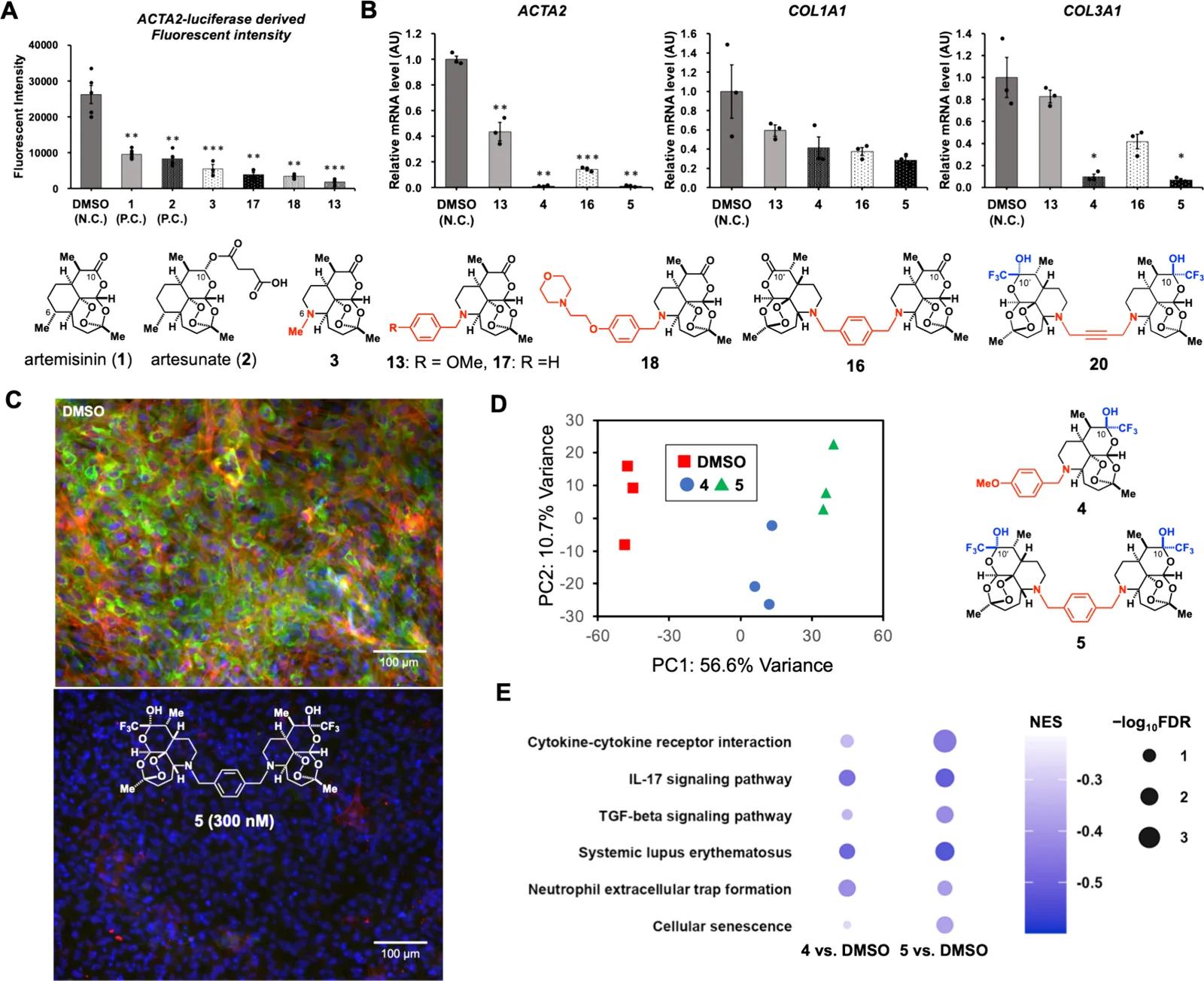
Antifibrotic activity and transcriptomic characterization of 6-aza-artemisinins in iPSC-derived quiescent hepatic stellate cells (qHSCs). (A) Screening of N6-substituted analogs (**3**, **13**, **17**, and **18**; 2 μM, 72 h) in *ACTA2*–luciferase reporter qHSCs. Data are presented as mean ± s.e. (*n* = 3–6). (B) qRT-PCR analysis of fibrosis-associated genes (*ACTA2*, *COL1A1*, and *COL3A1*) in qHSCs treated with artesunate (**2**) and 6-aza-artemisinins (**4**, **5**, **13**, and 16; 2 μM, 72 h). Data are presented as mean ± s.e. (*n* = 3). Gene expression levels were normalized to *GAPDH*. Additional evaluation of the N6, N6′-linked dimer **20**, including its synthesis and activity comparison with dimer **5**, is provided in Figures S4 and S5. (C) Immunofluorescence imaging of qHSCs treated with DMSO or the dimer **5** (300 nM). DMSO controls displayed strong ACTA2/α-SMA (red) and collagen I (green, antibody staining) signals, whereas treatment with **5** markedly reduced both. Nuclei stained with DAPI (blue). Scale bars, 100 μm. (D) Principal component analysis (PCA) of global transcriptomic profiles demonstrating distinct clustering of qHSCs treated with **4**, **5**, or DMSO. (E) Bubble plot showing GSEA results highlighting fibrosis-related KEGG pathways that were significantly down-regulated in iPSC-derived qHSCs treated with **4** or **5** compared with DMSO. Color indicates normalized enrichment score (NES), and dot size represents – log_10_FDR. * *p* < 0.05, ** *p* < 0.01, *** *p* < 0.001.

### Synergistic Effect of C10 Trifluoromethylation and N6–N6′ Dimerization in Enhancing *In Vitro* Antifibrotic Activity

Guided by the SAR trends identified for N6 substitution, introduction of a trifluoromethyl group at C10 of **13** afforded the monomeric analog **4**, which showed markedly improved suppression of *ACTA2*, *COL1A1*, and *COL3A1* expression compared with its parent monomer **13** (Figure 2B). This enhancement is plausibly attributed to the sterically demanding, strongly electron-withdrawing CF_3_ group, which reduces susceptibility to C10 glucuronidation and enhances metabolic stability. Subsequent N6–N6′ dimerization of C10 trifluoromethylated unit via a para-substituted benzyl linker furnished **5**—the first dimeric artemisinin analogue with a *C*_2_-symmetric framework bearing two trifluoromethylated hemiketals at the C10 and C10′ positions. Notably, this dual-CF_3_ design resulted in a striking gain in potency, establishing **5** as the most active antifibrotic agent in the series, with activity surpassing artesunate (**2**) and all other 6-aza-artemisinin derivatives tested (Figure 2B). In contrast, the corresponding N6–N6′ dimer retaining unmodified C10/C10′ lactones **16** exhibited lower activity, underscoring that the dual C10/C10′ CF_3_ substitution combined with N6–N6′ dimerization is essential to achieve the full synergistic enhancement of antifibrotic potency.

To further investigate the impact of linker structure, we synthesized an N–N′-linked dimer containing a 2-butyne linker (**20**) (Figure S4). In iPSC-derived qHSCs, **20** exhibited weaker antifibrotic activity than the 1,4-benzyl-linked dimer **5** (Figure S5). These findings suggest that dimerization alone is insufficient for potent activity and that linker structure plays an important role in determining antifibrotic efficacy, thereby supporting the use of the 1,4-benzyl linker in our dimer design.

### Direct Imaging Evidence for the Potent Antifibrotic Action of Dimer 5 in iPSC-Derived HSCs

Consistent with the quantitative findings, immunofluorescence imaging of iPSC-derived quiescent HSCs demonstrated the marked suppression of fibrogenic markers following treatment with dimer **5**. After 2 days of culture, DMSO-treated control cells (Figure 2C) exhibited ACTA2/α-SMA (red) and collagen I (green) signals, indicating myofibroblastic activation and collagen I accumulation under the present culture conditions. In contrast, treatment with 300 nM dimer **5** (Figure 2C) markedly reduced both markers; while faint ACTA2/α-SMA (red) remained detectable, collagen I staining was strongly reduced. These findings are consistent with the potent inhibitory activity of dimer **5** on HSC activation and collagen I accumulation under the present culture conditions. The present data do not distinguish whether the reduced collagen I staining resulted from suppression of collagen I accumulation or active removal of pre-existing extracellular matrix. Therefore, the present findings should not be interpreted as evidence for the latter.

To exclude the possibility that the marked reduction in ACTA2/α-SMA and collagen I staining simply reflected compound-induced cytotoxicity, we further performed live/dead viability staining of iPSC-derived qHSCs treated with three representative compounds (**4**, **5**, and **20**) at 500 nM for 72 h (Figure S6). Calcein/Ethidium staining showed abundant Calcein-positive viable cells and only a few Ethidium-positive dead cells in the compound-treated groups, in contrast to the saponin-treated positive control. These results indicate that the marked reduction in ACTA2/α-SMA and collagen I staining observed following treatment with dimer **5** under the present conditions was not primarily attributable to overt cytotoxicity.

### Transcriptomic Analysis Reveals Shared Adaptive Responses of 6-Aza-artemisinins and a Deeper Antifibrotic Signaling Suppression by Dimer 5

To gain mechanistic insight into how 6-aza-artemisinins exert their potent antifibrotic activity, we profiled transcriptional responses in iPSC-derived quiescent HSCs treated with DMSO, **4**, or **5**. Global transcriptomic analyses—including principal component analysis and differential expression mapping—revealed that all three treatment groups segregated into well-separated clusters (Figures 2D, S7A–C), demonstrating that each compound induces distinct transcriptional states relative to the control and one another.

Gene set enrichment analysis (GSEA)^28^ against KEGG pathways revealed broad and coordinated down-regulation of transcriptional programs associated with core profibrotic signaling modules (Figure 2E), including cytokine–cytokine receptor interaction, IL-17 signaling, and TGF-β signaling—central axes that drive fibroblast activation, inflammatory amplification, and extracellular matrix production. Beyond these canonical pathways, both **4** and **5** suppressed modules associated with autoimmune inflammation, including systemic lupus erythematosus, as well as pathways related to cellular senescence^29,30^ and neutrophil extracellular trap (NET) formation,^31,32^ both increasingly recognized as contributors to fibrotic progression. Consistently, the dimer **5** induced deeper repression across fibrosis-related pathways than the monomer **4**, in agreement with its superior cellular potency. Direct comparison of transcriptomic profiles by GSEA further revealed additional fibrosis-related pathways that were preferentially suppressed by the dimer **5** (Figure S7D), consistent with its enhanced antifibrotic potency in functional assays. Pathway-level mapping likewise demonstrated coordinated down-regulation of multiple nodes across the TGF-β, IL-17, and Toll-like receptor–mediated inflammatory signaling cascades (Figures S8–S14), supporting coordinated transcriptional attenuation of fibrosis- and inflammation-associated pathways rather than isolated gene-specific effects. Together, these transcriptional signatures indicate that the antifibrotic effects of **4** and **5** extend beyond suppression of HSC activation, engaging conserved molecular circuits implicated in autoimmune and systemic fibrotic diseases (Figure S8).

To functionally validate the transcriptomic suppression of TGF-β-associated profibrotic pathways, we further examined the effects of **4** and **5** under exogenous TGF-β stimulation in iPSC-derived qHSCs. Under these conditions, TGF-β caused only modest additional induction of fibrogenic marker genes in DMSO-treated cells, consistent with the cells already exhibiting a substantially activated fibrogenic state under the culture conditions used. Nevertheless, dimer **5** maintained low *ACTA2* expression in both the absence and presence of TGF-β, while its suppression of *COL1A1* was partially overridden by exogenous TGF-β (Figure S15). Together, these results suggest that dimer **5** attenuates the activated fibrogenic state of iPSC-derived qHSCs without simply behaving as a direct ALK5/TGF-β receptor inhibitor. We next evaluated the translational relevance of these findings in dermal fibroblasts derived from patients with SSc.

### Evaluation of Antifibrotic Activity in SSc Patient-Derived Dermal Fibroblasts

Building on the efficacy observed in iPSC-derived qHSCs, we next investigated the translational potential of these compounds in systemic sclerosis (SSc). SSc is a severe disorder characterized by skin thickening and progressive fibrosis of internal organs such as the heart and lungs, driven by a complex interplay of autoimmunity, vasculopathy, and dysregulated fibrogenesis. To date, no approved therapy has been shown to ameliorate established fibrosis, highlighting the urgent need for agents that directly target fibrotic pathways. Importantly, two independent lines of evidence support the translational relevance of the HSC-based findings to SSc. First, RNA-seq analysis in iPSC-derived qHSCs revealed that both compounds down-regulate transcriptional programs associated with core profibrotic pathways that are also fundamental to SSc pathogenesis, including cytokine receptor interaction, IL-17 signaling, and TGF-β signaling (Figure 2E). Second, HSCs and dermal or visceral fibroblasts share a conserved TGF-β–driven activation signature, characterized by induction of ACTA2 and COL1A1.^2,3^ Together, these observations indicate that the transcriptional networks attenuated by 6-aza-artemisinin derivatives in HSCs overlap with molecular programs relevant to fibroblast activation in SSc.

This mechanistic alignment prompted us to validate the antifibrotic activity of 6-aza-artemisinin derivatives in dermal fibroblasts from patients with SSc, thereby bridging preclinical screening with clinically relevant models. Gene expression of fibrotic markers (*ACTA2* and *COL1A1*) was quantified by RT-qPCR and normalized to *GAPDH*. All compounds tested, including artesunate (**2**) and 6-aza-artemisinins (**4** and **5**), elicited a dose-dependent suppression of fibrotic gene expression (Figure 3A). Notably, the dimeric analog **5** exhibited pronounced potency, significantly reducing transcript levels even at 0.1 μM—a concentration at which artesunate (**2**) and **4** (monomer) were inactive. Moreover, the N–N′-linked dimer **20** containing a 2-butyne linker exhibited substantially weaker antifibrotic activity than the corresponding 1,4-benzyl-linked dimer **5** in SSc patient-derived dermal fibroblasts (Figure S16). These results demonstrate that **5** (dimer) exerts superior antifibrotic activity in patient-derived dermal fibroblasts, consistent with its pronounced activity in iPSC-derived qHSCs.

**Figure 3:**
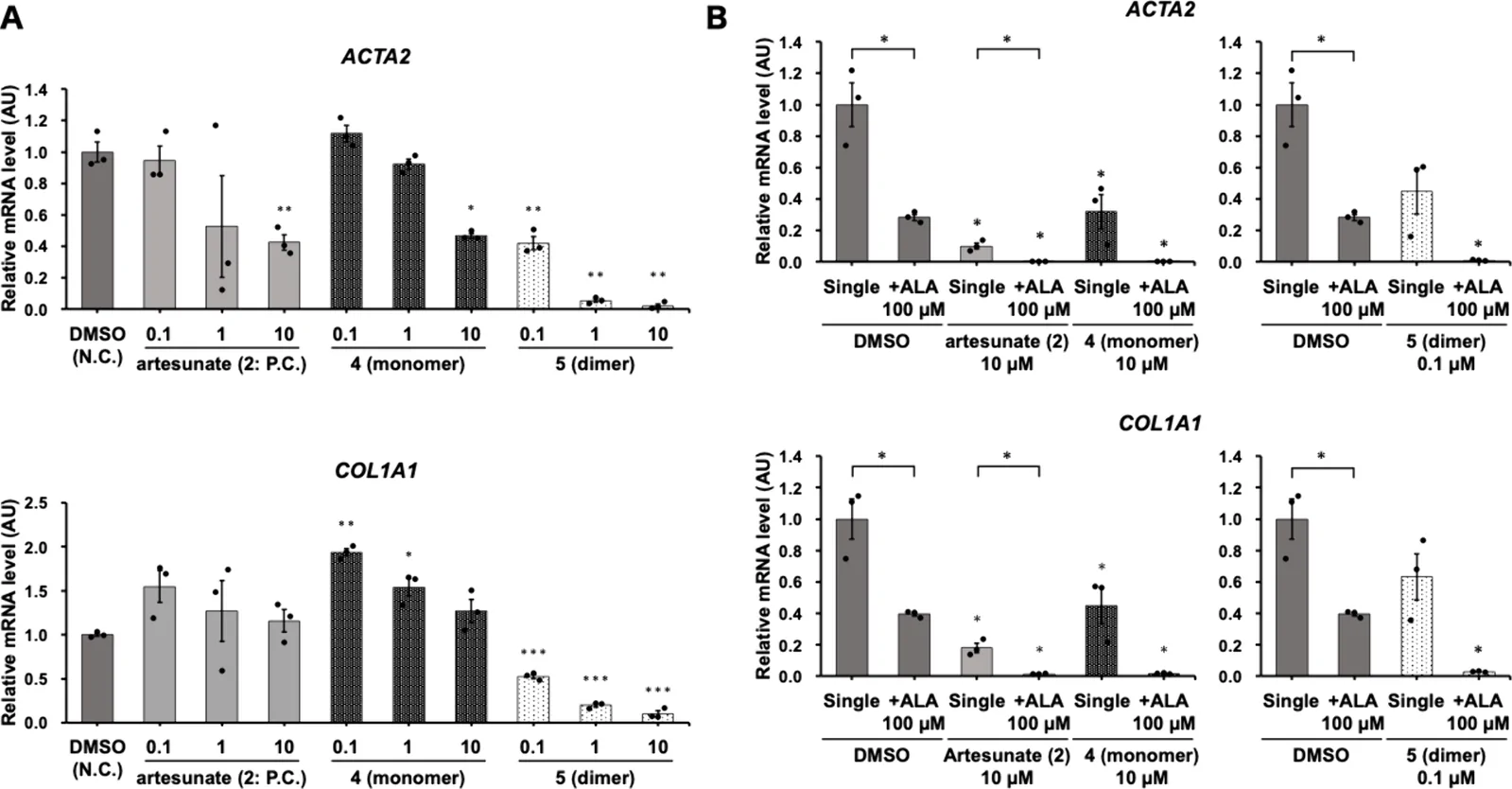
Antifibrotic evaluation of 6-aza-artemisinins in dermal fibroblasts derived from a patient with systemic sclerosis (SSc). (A) qRT-PCR analysis of fibrosis-associated genes (*ACTA2*, *COL1A1*) in SSc fibroblasts treated with artesunate (**2**), 6-aza-artemisinins **4** (monomer) or **5** (dimer) at 0.1, 1, and 10 μM for 72 h. The antifibrotic activity of the N–N′-linked dimer **20** relative to **5** is shown in Figure S16. (B) qRT-PCR analysis of the same genes in SSc fibroblasts treated with **2** (10 μM), **4** (10 μM), or **5** (0.1 μM) alone or in combination with ALA (100 μM) for 72 h. Gene expression levels were normalized to *GAPDH*. Data are presented as mean ± s.e. (*n* = 3 independent culture wells from the same patient-derived fibroblast line). Each dot represents one independent well. In (B), asterisks above individual bars indicate statistical significance compared with the DMSO control, whereas bracketed comparisons indicate statistical significance between the corresponding conditions in the presence or absence of ALA. * *p* < 0.05, ** *p* < 0.01, *** *p* < 0.001.

### Evaluation of Antifibrotic Activity in SSc Fibroblasts in Combination with 5-Aminolevulinic Acid (ALA)

The pharmacological activity of antimalarial artemisinin derivatives is largely dependent on reductive cleavage of the endoperoxide bridge by ferrous (Fe^2+^) species, such as heme iron.^33,34^ Because 5-aminolevulinic acid (ALA) serves as a biosynthetic precursor of heme, coadministration of ALA has been reported to potentiate the anticancer efficacy of artesunate (**2**).^35^ We therefore examined whether ALA could enhance the antifibrotic activity of fully synthetic 6-aza-artemisinins (**4** and **5**) in dermal fibroblasts derived from patients with SSc.

Fibroblasts were treated with artesunate (**2**) and the lead candidates **4** (monomer) and **5** (dimer) in the presence or absence of ALA (100 μM), and expression levels of fibrosis-related genes were quantified after 72 h (Figure 3B). To assess combination effects, the ALA concentration was fixed at 100 μM, while compound concentrations were selected based on the preceding results such that single-agent treatment reduced fibrosis marker expression by approximately 50%. The ALA concentration was selected as a proof-of-concept condition to evaluate the potential contribution of heme biosynthesis to the activity of aza-artemisinins and was not intended to define a therapeutically optimized combination regimen. Accordingly, artesunate (**2**) and **4** (monomer) were tested at 10 μM, whereas the markedly more potent **5** (dimer) was evaluated at 0.1 μM. For clarity, these conditions were also shown in separate graphs. ALA alone modestly reduced several fibrosis markers, but its effect was consistently weaker than that of artemisinin derivatives and was further enhanced upon combination.

Consistent with this trend, artesunate (**2**) suppressed *COL1A1* expression to approximately 20% of the control, and this effect was potentiated by ALA cotreatment, reducing *COL1A1* expression to nearly 2%. Building on this observation, both **4** and **5** exhibited enhanced activity in the presence of ALA. Notably, **4** (monomer) combined with ALA reduced *ACTA2* and *COL1A1* expression to less than 10% of control (*p* < 0.05), highlighting the potentiating effect of ALA. Consistent with the results obtained using iPSC-derived HSCs, **5** (dimer) achieved near-complete suppression of profibrotic gene expression at 0.1 μM (<10% of control), confirming its exceptional potency. The reproducibility of these effects in both iPSC-derived HSCs and patient-derived dermal fibroblasts underscores the broad antifibrotic efficacy of 6-aza-artemisinins across organ systems. In addition, ALA coadministration, particularly with **4** (monomer), further potentiated these responses, suggesting translational potential given its established clinical use and safety profile. Interestingly, the potentiating effect of ALA was more pronounced for monomer **4** than for dimer **5**. Given the exceptionally high intrinsic activity of dimer **5**, this observation may reflect partial saturation of the biological response and/or differences in the contribution of heme-dependent activation. Further mechanistic studies will be required to distinguish between these possibilities.

### Metabolic Stability of Lead 6-Aza-artemisinin Analogs

To further assess the pharmacological potential of the lead compounds, microsomal metabolic stability was evaluated using mouse and human liver microsomes (Table S3). Species-dependent but informative trends were observed among the 6-aza-artemisinin analogs. In mouse liver microsomes, the compounds **4** and **16** exhibited high intrinsic clearance, whereas the C10/C10′-trifluoromethylated N6–N6′ dimer **5** showed markedly reduced clearance. The alkyne-linked dimer **20** displayed intermediate clearance, indicating that dimerization alone was insufficient to achieve the metabolic stability observed for **5**. In human liver microsomes, the N6–N6′ dimer **5** again exhibited the lowest intrinsic clearance among the 6-aza-artemisinin derivatives evaluated, whereas compounds **16** and **20** showed substantially higher clearance values. The monomeric analog **4** also displayed relatively low clearance, although it remained less stable than the dimer **5**. Notably, the dimer **5** exhibited approximately 25-fold lower intrinsic clearance than either **4** or **16** in mouse liver microsomes and showed lower clearance than both artemisinin (**1**) and artesunate (**2**) under the same assay conditions. Collectively, these results demonstrate that the combination of C10/C10′ trifluoromethylation and the N6–N6′ benzyl-linked dimeric architecture provides the greatest improvement in microsomal metabolic stability, with the dimer **5** exhibiting the highest metabolic stability among all compounds evaluated. Together with its potent antifibrotic activity, the favorable microsomal stability of dimer **5** further supported its prioritization for subsequent *in vivo* evaluation.

### In Vivo Antifibrotic Efficacy of Synthetic 6-Aza-artemisinins in a Bleomycin-Induced Skin Fibrosis Model

Building on the robust *in vitro* evidence, we next evaluated the *in vivo* antifibrotic efficacy of fully synthetic 6-aza-artemisinins (**4** and **5**) in SSc–related skin fibrosis models to validate their therapeutic potential (Figures 4–5 and S17). The monomeric analog **4** showed enhanced *in vitro* activity in SSc patient-derived dermal fibroblasts when combined with 5-aminolevulinic acid (ALA), whereas the dimeric analog **5** alone displayed high potency at submicromolar concentrations (Figure 3). Given the well-established clinical safety of ALA and its dual role in promoting mitochondrial heme biosynthesis and maintaining redox homeostasis, its combination with **4** provided a mechanistically rational strategy to augment antifibrotic efficacy. To assess prophylactic efficacy, we employed a bleomycin (BLM)-induced skin fibrosis model, with the treatment schedule illustrated in Figure S17A. Mice were treated with BLM (200 μg) together with **4** (5 mg/kg, racemate), while ALA (1.5 mg/mL) was supplied ad libitum in the drinking water. Histological analysis using hematoxylin and eosin (H&E) and Masson’s trichrome staining revealed that cotreatment with **4** + ALA effectively preserved dermal architecture, markedly reducing collagen bundle density and dermal thickening relative to vehicle-treated controls (Figure S17B).

**Figure 4:**
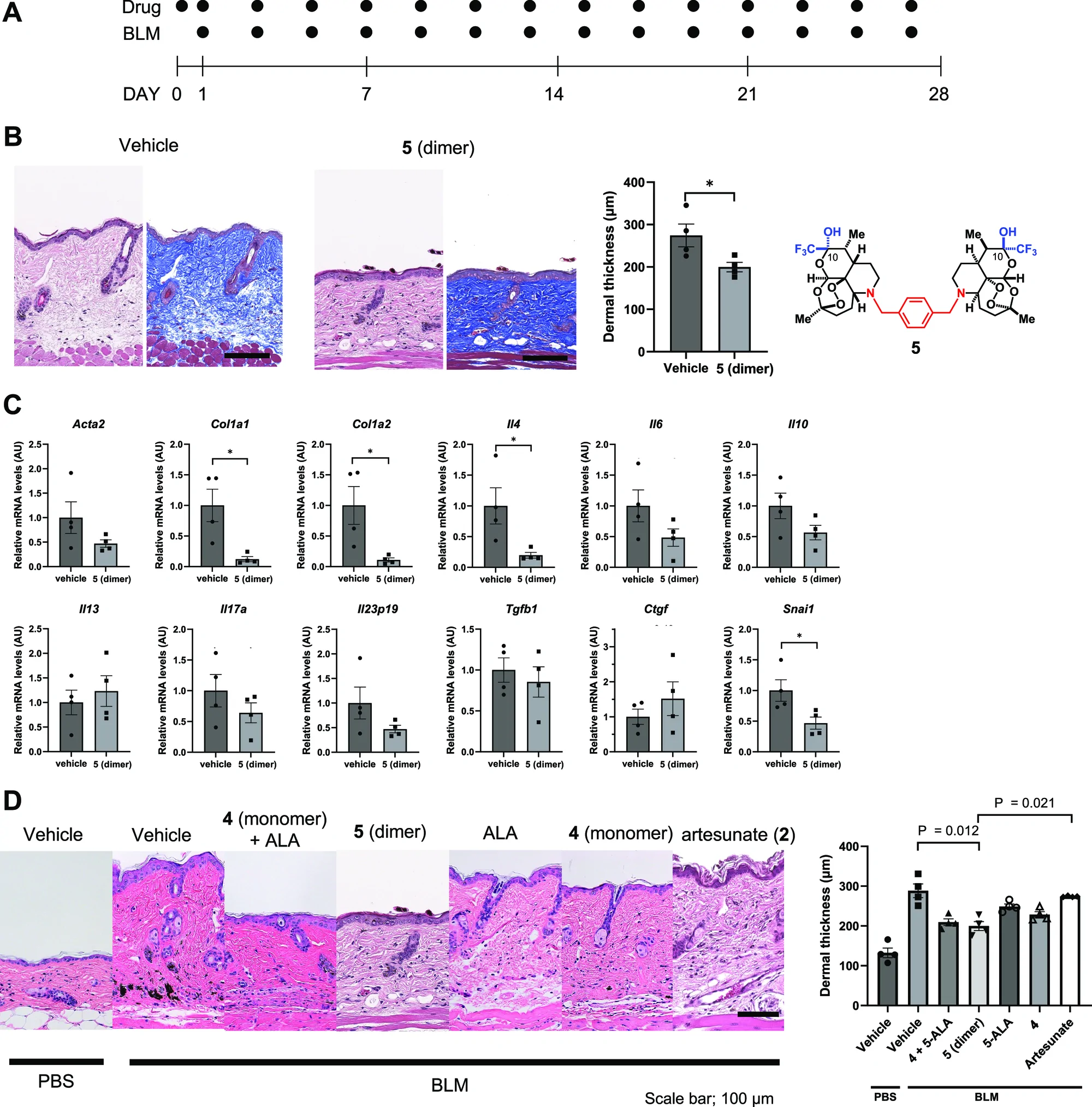
Dimer **5** (0.3 mg/kg) ameliorated skin fibrosis in the prophylactic model. (A) Treatment schedule of the prophylactic model. (B) Representative histological images showing dermal thickness in BLM-treated mouse skin following treatment with vehicle or **5** (dimer). Scale bar, 100 μm. (C) qRT-PCR analysis of fibrosis- and cytokine-related gene expression in skin tissue from mice treated with vehicle or **5** (0.3 mg/kg). (D) Comparative dermal thickness in PBS- or BLM-treated mice receiving vehicle or various artemisinin derivatives. Scale bar,100 μm. Abbreviations: BLM, bleomycin; ALA, 5-aminolevulinic acid. Data are presented as mean ± s.e. (*n* = 4). Gene expression levels were normalized to *Gapdh*. * *p* < 0.05, ** *p* < 0.01, *** *p* < 0.001.

**Figure 5:**
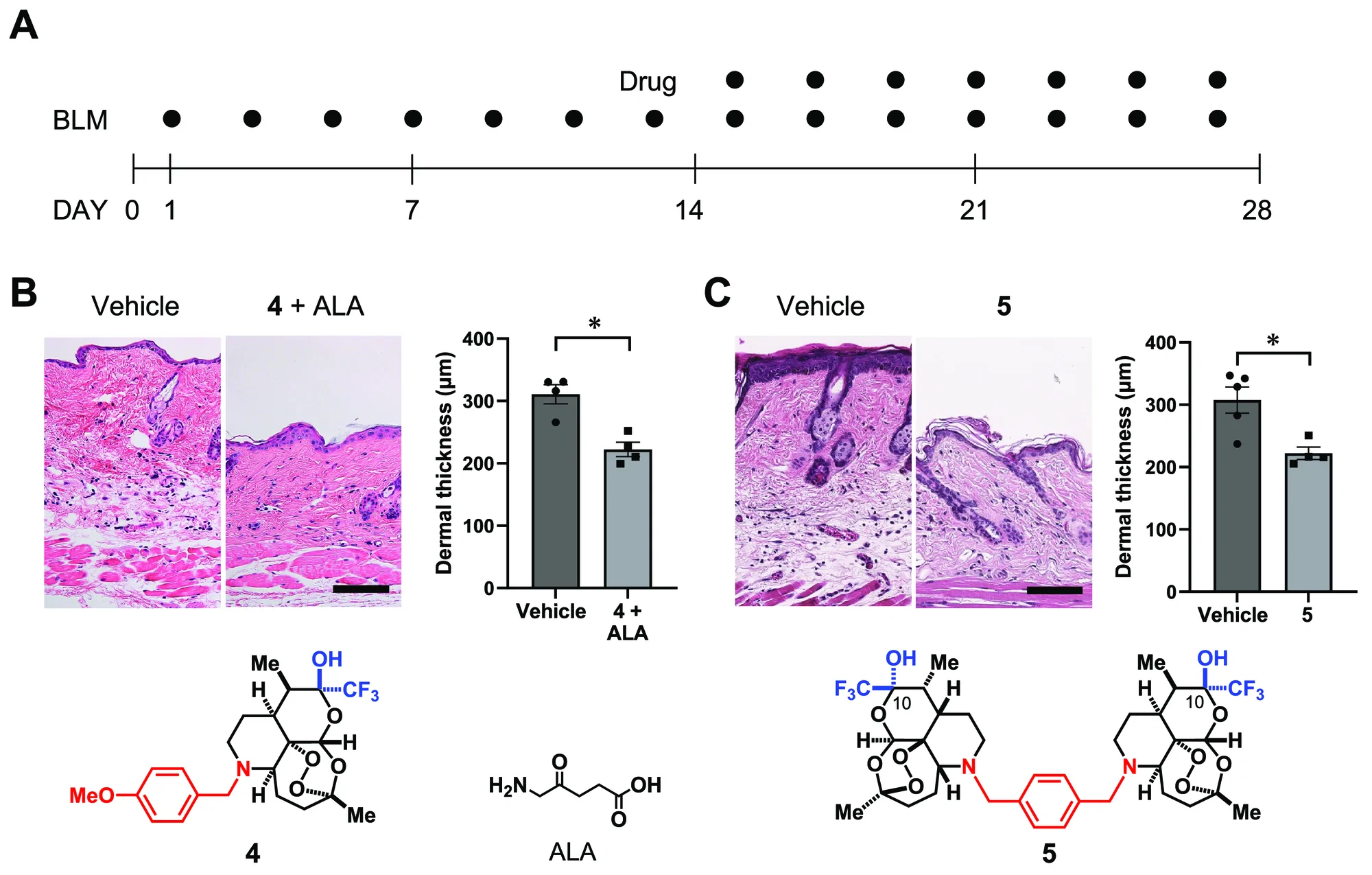
Monomer **4** (5 mg/kg) with 5-aminolevulinic acid (ALA) and dimer **5** (0.3 mg/kg) ameliorated skin fibrosis in the treatment model. (A) The treatment schedule of the treatment model. Dermal thickness in BLM-treated mouse skin following treatment with (B) monomer **4** + ALA, or (C) dimer **5**. Scale bar = 100 μm. BLM, bleomycin. Data are presented as mean ± s.e. (*n* = 4). * *p* < 0.05, ** *p* < 0.01, *** *p* < 0.001.

Consistent with these morphological improvements, qRT-PCR analysis of whole-skin samples demonstrated broad suppression of profibrotic (*Col1a2*, *Tgfb1*, *Acta2*) and proinflammatory (*Il4*, *Il6*, *Il13*) genes (Figure S17C), indicative of inhibition of the TGF-β signaling axis. Immunohistochemical analysis further confirmed a pronounced reduction in α-SMA–positive myofibroblasts within the dermis (Figure S17D), reflecting effective attenuation of fibroblast activation.

Although antimalarial artemisinins are known to undergo reductive activation through reaction between their endoperoxide bridge and ferrous heme, generating radical intermediates that may elevate local oxidative stress, the present dosing regimen of the **4** + ALA combination produced no detectable signs of oxidative stress or inflammatory exacerbation. This suggests that the antifibrotic benefits of the combination occur within a therapeutically balanced redox window. Collectively, these findings establish the translational potential of this combination and motivate subsequent evaluation of the more potent dimeric 6-aza-artemisinin **5** even in the absence of ALA coadministration.

### Potent Antifibrotic Efficacy of the Dimeric 6-Aza-artemisinin 5 at Low Dosage

Parallel *in vivo* experiments demonstrated that the dimeric 6-aza-artemisinin **5** exerted remarkably potent antifibrotic activity even when administered alone at a low dose of 0.3 mg/kg (Figures 4A–B). Histological analysis revealed a striking restoration of dermal architecture, characterized by thinner, more regularly organized collagen bundles, along with a significant reduction in dermal thickness relative to vehicle-treated controls.

Notably, these effects were achieved without the coadministration of ALA, underscoring the intrinsic potency of the dimeric scaffold featuring an N6–N6′ linkage that is inaccessible through semisynthetic approaches relying on naturally occurring artemisinins.

At the molecular level, qRT-PCR analysis of whole-skin tissue revealed broad and statistically significant suppression of key profibrotic and proinflammatory transcripts, including *Col1a1*, *Col1a2*, *Il4*, and *Snai1* (Figure 4C). Notably, Snai1—a transcription factor critically involved in fibroblast activation and endothelial-to-mesenchymal transition (EndoMT)—was strongly suppressed, suggesting that the dimer **5** effectively disrupts multiple profibrotic signaling axes. Collectively, these results highlight the exceptional efficacy of dimer **5** in ameliorating skin fibrosis at submilligram-per-kilogram doses and reinforce that N6–N6′ dimerization synergistically amplifies antifibrotic potency beyond the levels achievable by monomeric analogs or ALA coadministration.

### Comparative and Therapeutic Validation of 6-Aza-artemisinins *In Vivo*

Comparative evaluation across all treatment groups demonstrated that both **4** (monomer, 5 mg/kg) + ALA and **5** (dimer, 0.3 mg/kg) achieved the most pronounced attenuation of dermal fibrosis, far exceeding the effects of artesunate (**2**, 4.2 mg/kg) and ALA alone (Figure 4D). **4** (M.W. 459.46, 5.00 mg = 10.9 μmol) and artesunate (**2**) (M.W. 384.43, 4.19 mg = 10.9 μmol) were administered at equimolar doses. Notably, the artesunate dose used here (4.2 mg/kg) is within a pharmacologically meaningful range relative to clinically used human doses for severe malaria (2.4 mg/kg per dose),^36^ supporting the translational validity of the *in vivo* comparison despite inherent interspecies differences. Histological analyses revealed nearly complete preservation of dermal architecture, with collagen bundles appearing thinner and more regularly aligned, consistent with a marked reduction in dermal thickening. These outcomes support the notion that ALA coadministration enhances mitochondrial heme-dependent redox homeostasis, thereby synergizing with the intrinsic reactivity of the 6-aza-artemisinin scaffold, whereas dimer **5** alone achieves comparable or superior efficacy through its conformationally preorganized dual-endoperoxide pharmacophores (Figure 4D).

To further establish the therapeutic relevance of these findings, we next evaluated a treatment model in which 6-aza-artemisinin administration was initiated after the establishment of fibrosis. Under these conditions, both **4** + ALA (5 mg/kg) and **5** (0.3 mg/kg) produced reproducible histological improvements in dermal fibrosis (Figure 5). Notably, these effects were observed when treatment was initiated after fibrosis establishment, highlighting the clinical relevance of targeting established fibrotic pathology rather than disease prevention. The consistency of efficacy across both prophylactic and therapeutic paradigms underscores the robustness and translational potential of these treatment strategies. Overall, these findings establish 6-aza-artemisinins as a new chemotype capable of ameliorating fibrotic pathology, providing a framework for further therapeutic development *in vivo*.

## Discussion

### Integrative Discovery Platform Bridging iPSC-Based Screening, Synthetic Innovation, and *In Vivo* Therapeutic Validation

Systemic sclerosis (SSc) represents one of the most intractable fibrotic disorders, reflecting a profound unmet medical need. Current therapeutic options remain largely symptomatic, focusing on inflammation or immune activation rather than the irreversible fibrotic process itself. Although biological agents such as tocilizumab (anti-IL-6 receptor antibody)^37^ and rituximab (anti-CD20 antibody)^38^ have offered partial clinical benefits, their limited ability to reverse established fibrosis underscores the need for new therapeutic strategies that act directly on fibroblast activation and tissue remodeling.^4,5^ To address this central pathogenic driver, we sought to develop a small-molecule approach that engages the core fibrotic machinery, rather than merely modulating its associated inflammatory components.

To this end, we constructed a multidisciplinary discovery pipeline integrating stem cell biology, synthetic chemistry, and disease modeling. Using human iPSC-derived qHSCs equipped with an *ACTA2*–luciferase reporter, we established a high-sensitivity screening platform capable of quantitatively monitoring myofibroblast activation (Figure 1A). This system faithfully recapitulates the key profibrotic features of SSc, providing a translationally relevant model for early phase compound evaluation. Through this screening, we identified the antimalarial natural product artemisinin (**1**) as a reproducible antifibrotic hit, which prompted us to redesign the tetracyclic scaffold, leading to fully synthetic 6-aza-artemisinins. Strategic nitrogen incorporation at the C6 position and late-stage trifluoromethylation at C10 afforded analogs with improved metabolic stability, tunable physicochemical properties, and synthetic versatility—including the creation of N6–N6′ dimers.

This strategic elemental substitution created a new synthetic handle enabling systematic modification at both the N6 and C10 positions, including dimerization via N6–N6′ linkage—an otherwise inaccessible feature in natural artemisinins (Figure 1). The resulting modular platform enabled simultaneous optimization of biological activity and metabolic stability. Consistent with the microsomal stability studies, the benzyl-linked dimer **5** exhibited substantially lower intrinsic clearance than both the corresponding nontrifluoromethylated dimer **16** and the alkyne-linked dimer **20**, indicating that both C10/C10′ trifluoromethylation and linker architecture contribute to the favorable metabolic profile (Table S3). These rational modifications generated monomeric (**4**) and dimeric (**5**) analogs with substantially improved antifibrotic potency relative to artemisinin (**1**) and its semisynthetic derivative artesunate (**2**). Preliminary structure–activity analyses further revealed that *N*-benzyl substitution and N6–N6′ dimerization correlate strongly with potency enhancement, thereby delineating the structural determinants underlying antifibrotic efficacy.

Importantly, the antifibrotic trends observed in iPSC-derived qHSCs were faithfully recapitulated in dermal fibroblasts derived from SSc patients, demonstrating that the pharmacological activity of 6-aza-artemisinins transcends tissue origin (Figures 2–3). These cellular findings were further validated by robust and reproducible efficacy in the bleomycin-induced skin fibrosis model, where both the monomer **4** + ALA combination and the dimer **5** markedly attenuated dermal fibrosis, substantially exceeding the effects of artemisinin-based drugs (Figures 4–5, S17). Collectively, these results embody the concept of bridging iPSC-derived phenotypic screening and *in vivo* efficacy, establishing a unified translational pipeline that connects phenotypic discovery with preclinical validation.

### Chemotype-Specific Activity and Translational Relevance of 6-Aza-artemisinins

Artemisinin and its derivatives exert broad biological activities far beyond their canonical antimalarial effects,^39^ including antifibrotic,^40,41^ anti-inflammatory, and immunomodulatory actions.^42^ Previous studies have shown that individual artemisinin derivatives can influence several signaling pathways relevant to fibrosis and immune regulation—such as TGF-β–Smad,^43,44^ PI3K–AKT,^45^ MD2/TLR4–NF-κB,^46–49^ and other innate immune pathways^42^—although the precise molecular targets appear to vary by compound and cellular context.^50^

Consistent with the transcriptomic evidence indicating suppression of inflammatory signaling pathways, dimer **5** also inhibited LPS-induced expression of the proinflammatory cytokines IL-1β and IL-6 in RAW264.7 macrophage-like cells (Figure S18). Although the direct molecular target remains to be identified, these findings suggest that modulation of inflammatory signaling pathways may contribute, at least in part, to the observed antifibrotic effects observed for this dimeric 6-aza-artemisinin series.

The monomeric regimen **4** + ALA showed broad suppression of profibrotic and cytokine-related markers, consistent with attenuation of activated fibroblast states. In contrast, the N6–N6′ dimer **5** exhibited superior potency across *in vitro* and *in vivo* assays and retained strong efficacy without ALA coadministration, highlighting the functional advantage conferred by scaffold-level dimerization. These findings suggest that chemical architecture, rather than simple derivatization of the natural product, plays a decisive role in shaping antifibrotic activity.

More broadly, the convergence of efficacy trends across iPSC-derived qHSCs, SSc patient-derived fibroblasts, and murine fibrosis models underscores the translational relevance of this platform. Thus, the present work establishes 6-aza-artemisinins not only as a new antifibrotic chemotype, but also as an example of how scaffold redesign can generate functionally differentiated small molecules with therapeutic relevance in complex fibrotic diseases.

## Conclusion

In summary, this study establishes 6-aza-artemisinins as a promising chemotype that extends the pharmacological scope of the artemisinin scaffold. Our *de novo* synthetic design provides concise and modular access to previously inaccessible nitrogen-embedded scaffolds, enabling systematic structural diversification and discovery of highly potent antifibrotic analogs. Among these, the N6–N6′ dimer **5** exhibited exceptional efficacy at a submilligram-per-kilogram dose in a murine bleomycin-induced skin fibrosis model, substantially outperforming the semisynthetic antimalarial drug artesunate (**2**).

Beyond these therapeutic implications, our work demonstrates a generalizable translational strategy that links rational molecular design with iPSC-based phenotypic screening and *in vivo* validation. By establishing a functional bridge between stem-cell-derived assays and disease-relevant therapeutic outcomes, we outline a practical route for accelerating the development of next-generation antifibrotic agents. Collectively, these findings highlight the artemisinin scaffold as a versatile platform for precision molecular editing and illustrate how rational scaffold remodeling of natural products can yield new chemotypes for regenerative and fibrotic medicine.

## Supplementary Material













## Notes

Detailed experimental procedures and characterization for all compounds are provided in the Supporting Information, which includes the following sections: 1. Supplementary Tables and Figures, 2. Organic Synthesis, 3. Methods for *In Vitro* Activity Evaluation, 4. Transcriptomic analysis in iPSC-derived qHSCs, 5. Methods for *In Vivo* Activity Evaluation, and 6. NMR Spectra (PDF)
Crystallographic data for compound **4** CCDC 2514366 (CIF)
Crystallographic data for compound **5** CCDC 2514367 (CIF)
Data set S1, which lists differentially expressed genes (DEGs) identified by RNA-seq analysis of iPSC-derived qHSCs: S1, monomer **4** vs DMSO (CSV)
Data set S2, which lists differentially expressed genes (DEGs) identified by RNA-seq analysis of iPSC-derived qHSCs: S2, dimer **5** vs DMSO (CSV)
Data set S3, which lists differentially expressed genes (DEGs) identified by RNA-seq analysis of iPSC-derived qHSCs: S3, dimer **5** vs monomer **4** (CSV)

## Notes

Deposition Numbers 2514366 and 2514367 contain the supplementary crystallographic data for this paper. These data can be obtained free of charge via the joint Cambridge Crystallographic Data Centre (CCDC) and Fachinformationszentrum Karlsruhe Access Structures service.
