# Supplementary material for: Integrated Scaffold Redesign and iPSC-Based Screening Reveal Potent Antifibrotic Artemisinin Analogs in Systemic Sclerosis Models
Source: ACS Cent Sci. 2026 Sep 12;12(9):1372–84. doi: 10.1021/acscentsci.6c00658 (PMC13614056; doi:10.1021/acscentsci.6c00658)
Supplement: Supplementary file 1 [file oc6c00658_si_001.pdf]

Electronic Supplementary Information

Integrated Scaffold Redesign and iPSC-Based Screening Reveal Potent Antifibrotic Artemisinin Analogs in Systemic Sclerosis Models

Takehiro Ishiga,[1]† Tetsuya Ikawa,[2]† Nobuto Kaneko,[1] Norihito Takahashi,[3] Krishanu Mondal,[1] Yasuhiro Nakano,[4] Yutaro Hori,[5] Atsushi Miyajima,[6] Taketomo Kido,[6]* Yoshihide Asano,[2]* Hiroki Oguri[1]*

[1] Department of Chemistry, Graduate School of Science, The University of Tokyo, 7-3-1, Hongo, Bunkyo-ku, Tokyo, Japan.

[2] Department of Dermatology, Graduate School of Medicine, Tohoku University, 1-1 Seiryo-machi, Aoba-ku, Sendai 980-8574, Japan.

[3] Department of Applied Chemistry, Graduate School of Engineering, Tokyo University of Agriculture and Technology, 2-24-16, Naka-cho, Koganei, Tokyo, Japan.

[4] Integrated Systems of Aging Research Unit, Institute for Frontier Science Initiative, Kanazawa University, Kakuma-machi, Kanazawa, Ishikawa, Japan.

[5] Laboratory of Genome Regeneration, Institute for Quantitative Biosciences, The University of Tokyo, 1-1-1 Yayoi, Bunkyo-ku, Tokyo 113-0032, Japan.

[6] Laboratory of Cell Growth and Differentiation, Institute for Quantitative Biosciences, The University of Tokyo, 1-1-1 Yayoi, Bunkyo-ku, Tokyo 113-0032, Japan.

*corresponding authors:

kido@iqb.u-tokyo.ac.jp, yasano@derma.med.tohoku.ac.jp, and hirokioguri@g.ecc.u-tokyo.ac.jp

1. Supplementary Tables and Figures 3
2. Organic Synthesis 23
  2.1 General Considerations 23
  2.2 Ozone oxidation to form 6-aza-artemisinin **13** 23
  2.3 Dimerization to form **16** 24
  2.4 Trifluoromethylation to form monomer **4** 25
  2.5 Trifluoromethylation to form dimer **5** 26
  2.6 Dimerization to form **19** 27
  2.7 Trifluoromethylation to form dimer **20** 27
3. Methods for *In Vitro* and ADME Evaluation 29
  3.1 Maintenance of human iPSCs 29
  3.2 Generation of *ACTA2*-Luciferase (*ACTA2*-Luc) reporter iPSC line 29
  3.3 Generation of qHSC-like cells from human iPSCs (iPSC-derived qHSCs) 29
  3.4 Luciferase reporter assay 30
  3.5 Compound treatment in iPSC-derived qHSCs 30
  3.6 Cell viability and cytotoxicity assay 30
  3.7 Compound treatment and TGF-β stimulation in iPSC-derived qHSCs 30
  3.8 Quantitative Reverse Transcription (qRT)-PCR 31
  3.9 Immunocytochemistry 31
  3.10 Primary dermal fibroblasts from systemic sclerosis (SSc) patients 31
  3.11 Compound treatment in dermal fibroblasts from SSc patients 31
  3.12 Microsomal metabolic stability assay 32
  3.13 Compound treatment for RAW264.7 murine macrophage cells 32
4. Transcriptomic analysis in iPSC-derived quiescent hepatic stellate cells 33
  4.1 Compound treatment for RNA extraction 33
  4.2 mRNA library preparation and sequencing 33
  4.3 Differential gene expression and pathway enrichment analyses 33
  4.4 Pathview-based KEGG pathway mapping 34
5. Methods for *In Vivo* Activity Evaluation 35
  5.1 Ethics statement 35
  5.2 Mice 35
  5.3 A BLM-induced murine model of skin fibrosis 35
  5.4 Dermal thickness measurement 35
  5.5 RNA isolation and quantitative reverse transcription (qRT)-PCR 35
  5.6 Immunohistochemistry 36
6. Statistical analysis 37
7. References 38
8. NMR Spectra 39

1. Supplementary Tables and Figures

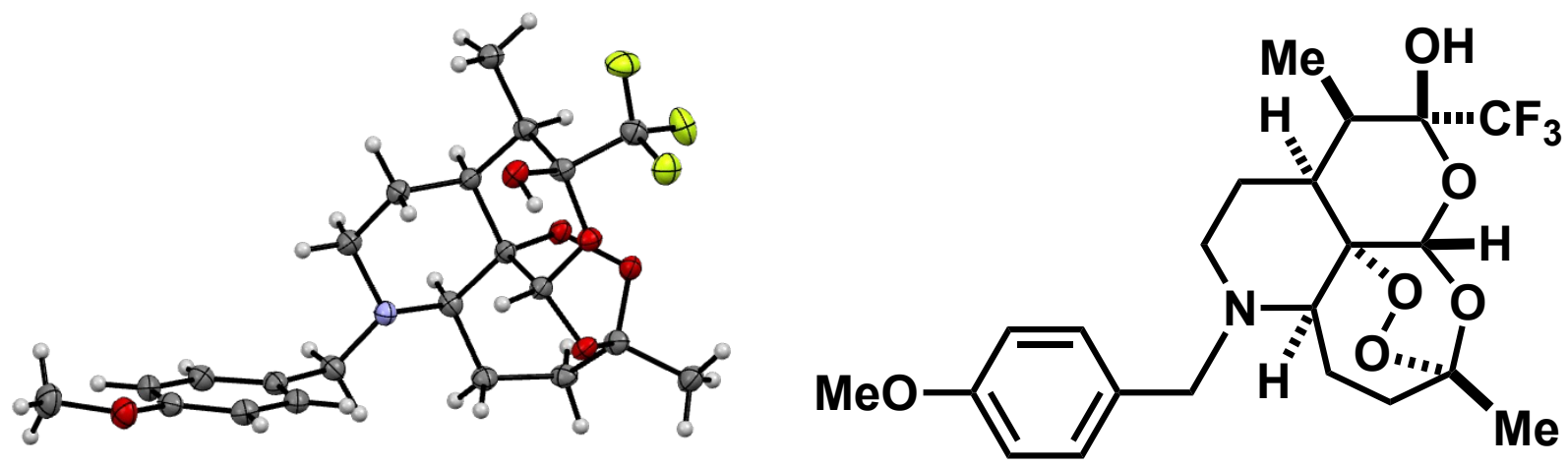

4

Figure S1. The molecular structure of monomer 4. Crystals of monomer **4** were obtained as colorless blocks by vapor diffusion of hexanes into a saturated CH_2Cl_2 solution at room temperature.

Table S1. Crystal data and structure refinement for compound 4.

Empirical formula	$C_{22}H_{28}NO_6F_3$
Formula weight	459.45
Temperature/K	100.15
Crystal system	triclinic
Space group	P-1
a/Å	9.6080(8)
b/Å	10.4818(8)
c/Å	11.8938(11)
α/°	88.335(4)
β/°	78.428(4)
γ/°	66.262(3)
Volume/Å^3	1072.40(16)
Z	2
Calculated density (g/cm^3)	1.423
Absorption coefficient (/mm)	0.119
F(000)	484.0
Crystal size/mm	0.1 × 0.1 × 0.05
Radiation	MoKα (λ = 0.71073)
2θ range for data collection/°	3.502 to 56.594
Limiting indices	-12 ≤ h ≤ 12, -13 ≤ k ≤ 13, -15 ≤ l ≤ 15
Reflections collected	80884
Independent reflections	5325 [R_{int} = 0.1484, R_{sigma} = 0.0547]
Data/restraints/parameters	5325/0/295
Final R indices [I>=2σ (I)]	R_1 = 0.0565, wR_2 = 0.1247
R indices [all data]	R_1 = 0.0957, wR_2 = 0.1701
Goodness-of-fit on F^2	1.109
Deposit number CCDC	2514366

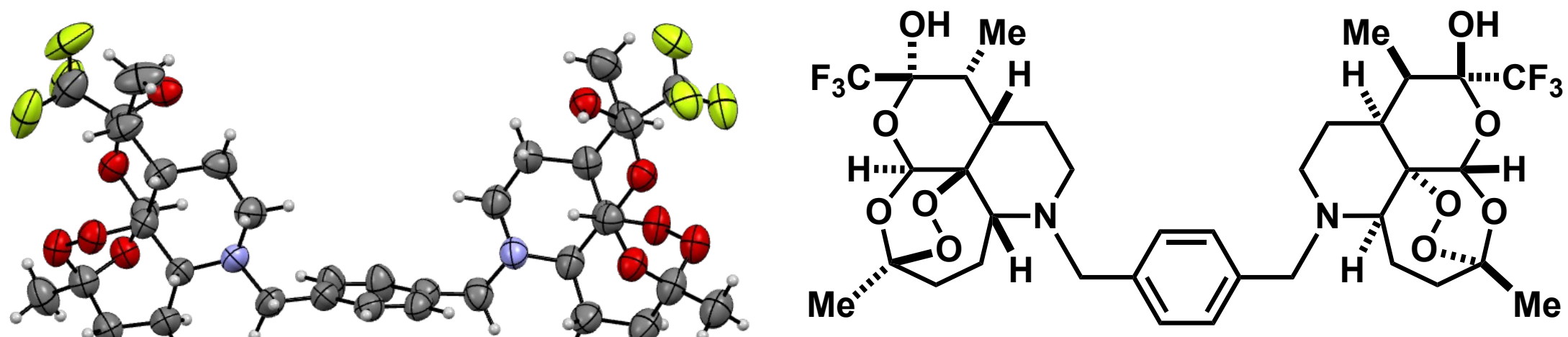

5

Figure S2. The molecular structure of dimer 5. Crystals of dimer **5** were obtained as colorless blocks by vapor diffusion of hexanes into a saturated $CHCl_3$ solution at 0 °C.

Table S2. Crystal data and structure refinement for compound 5.

Empirical formula	$4(C_{36}H_{46}F_6N_2O_{10})$
Formula weight	3122.98
Temperature/K	100.15
Crystal system	tetragonal
Space group	$P4_32_12$
a/Å	13.960(14)
b/Å	13.960(14)
c/Å	37.95(3)
α/°	90
β/°	90
γ/°	90
Volume/Å^3	7396(16)
Z	2
Calculated density (g/cm^3)	1.402
Absorption coefficient (/mm)	0.117
F(000)	3280.0
Crystal size/mm	0.1 × 0.1 × 0.05
Radiation	synchrotron (λ = 0.700)
2θ range for data collection/°	3.062 to 59.212
Limiting indices	$-19 \le h \le 19$, $-19 \le k \le 19$, $-52 \le l \le 53$
Reflections collected	122052
Independent reflections	10612 [R_{int} = 0.1827, R_{sigma} = 0.0700]
Data/restraints/parameters	10612/0/493
Final R indices [I>=2σ (I)]	R_1 = 0.0853, wR_2 = 0.2211
R indices [all data]	R_1 = 0.1298, wR_2 = 0.2530
Goodness-of-fit on F^2	0.943
Deposit number CCDC	2514367

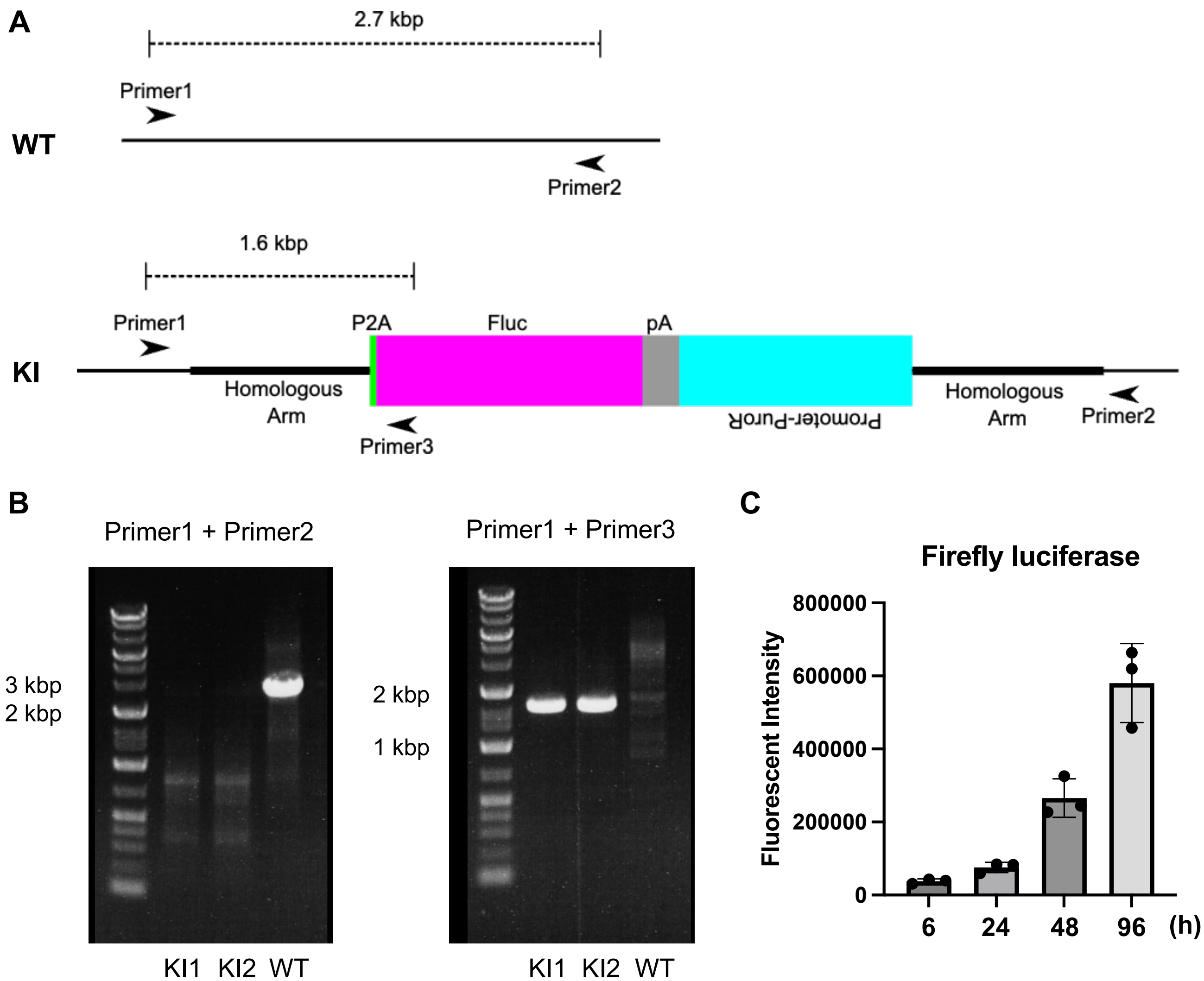

Figure S3. Generation and validation of an *ACTA2*-Luciferase reporter human iPSC line. (A) Schematic representation of the CRISPR-Cas9–mediated knock-in strategy at the human *ACTA2* locus. A P2A-linked firefly luciferase (Fluc) reporter gene, followed by a bidirectional SV40 polyadenylation signal and a puromycin resistance cassette, was inserted immediately upstream of the endogenous *ACTA2* stop codon via homologous recombination. Primer positions used for genotyping PCR are indicated. (B) PCR-based genotyping of representative *ACTA2*-Luc knock-in iPSC clones (KI1 and KI2). Primer1/Primer2 amplifies the wild-type allele, whereas Primer1/Primer3 specifically detects the targeted knock-in allele. WT, parental iPSCs. (C) Time-dependent increase in firefly luciferase activity in hepatic stellate cells derived from *ACTA2*-Luc reporter iPSCs following activation under two-dimensional culture conditions. Data represent mean ± s.d. (n = 3. Detailed experimental procedures related to this figure are provided in Section 3.2.

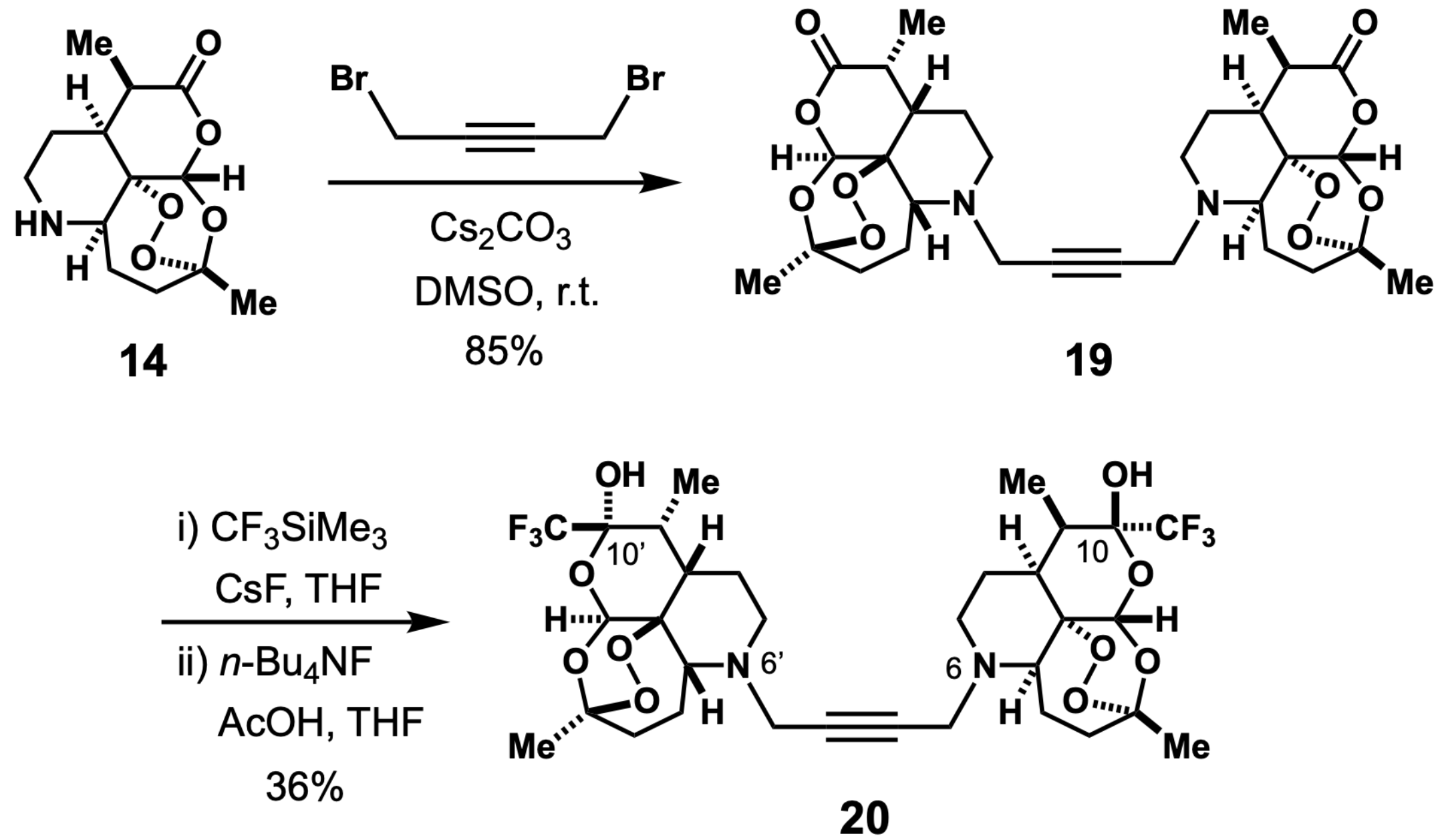

Figure S4. Synthetic routes to N6-linked dimers 19 and 20.

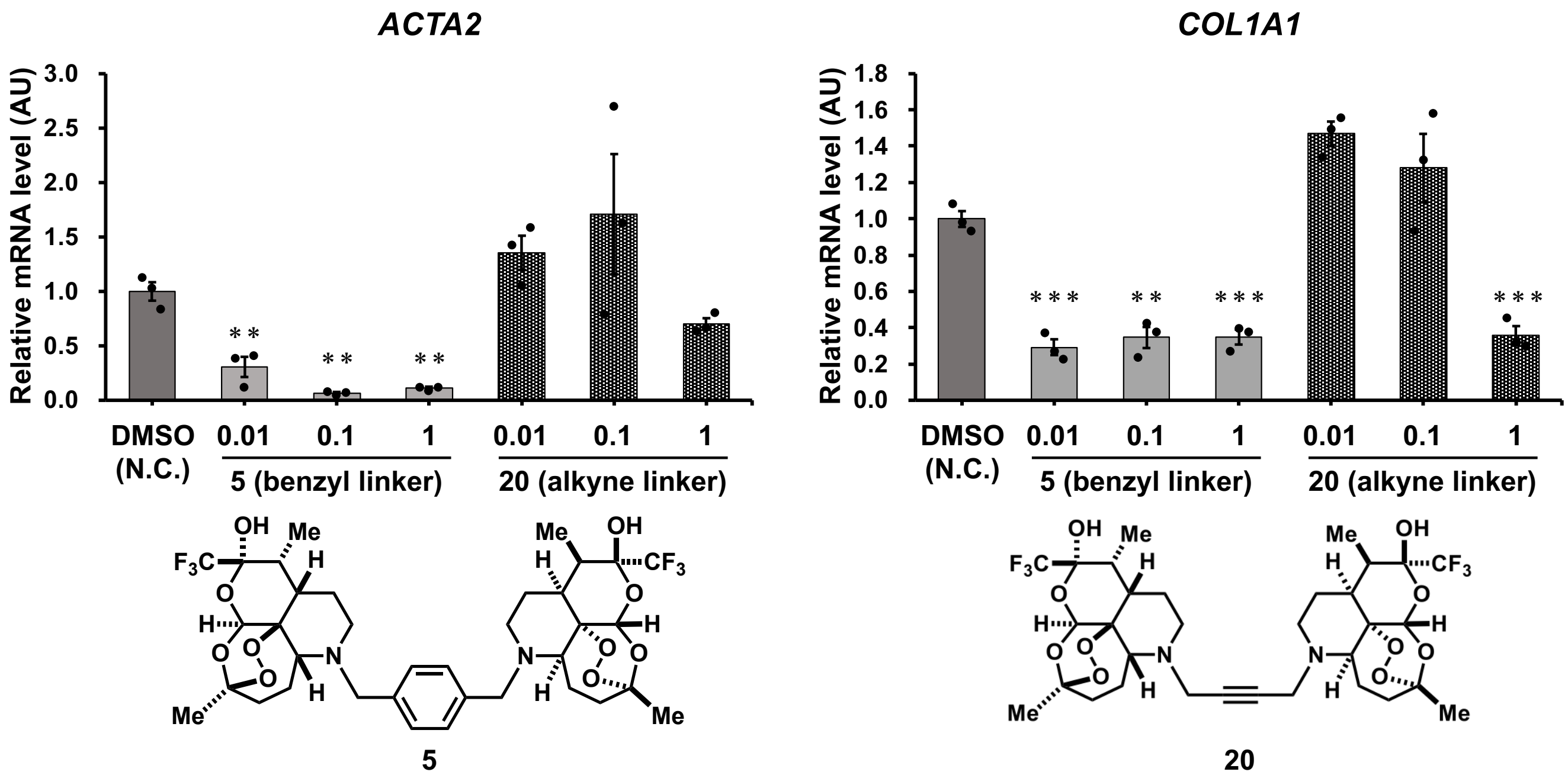

Figure S5. Linker-dependent antifibrotic activity of 6-aza-artemisinin dimers in iPSC-derived quiescent hepatic stellate cells (qHSCs). qRT-PCR analysis of fibrosis-associated genes (*ACTA2*, *COL1A1*) in iPSC-derived qHSCs treated with the benzyl-linked dimer **5** or the alkyne-linked dimer **20** at 0.01, 0.1, and 1 μM for 72 h. Gene expression levels were normalized to *GAPDH* and are shown relative to the DMSO-treated negative control. Data are presented as mean ± s.e. (n = 3 independent culture wells from the same differentiation batch of iPSC-derived qHSCs). Each dot represents one independent well and asterisks above individual bars indicate statistical significance compared with the DMSO control. * $p<0.05$, ** $p<0.01$, *** $p<0.001$.

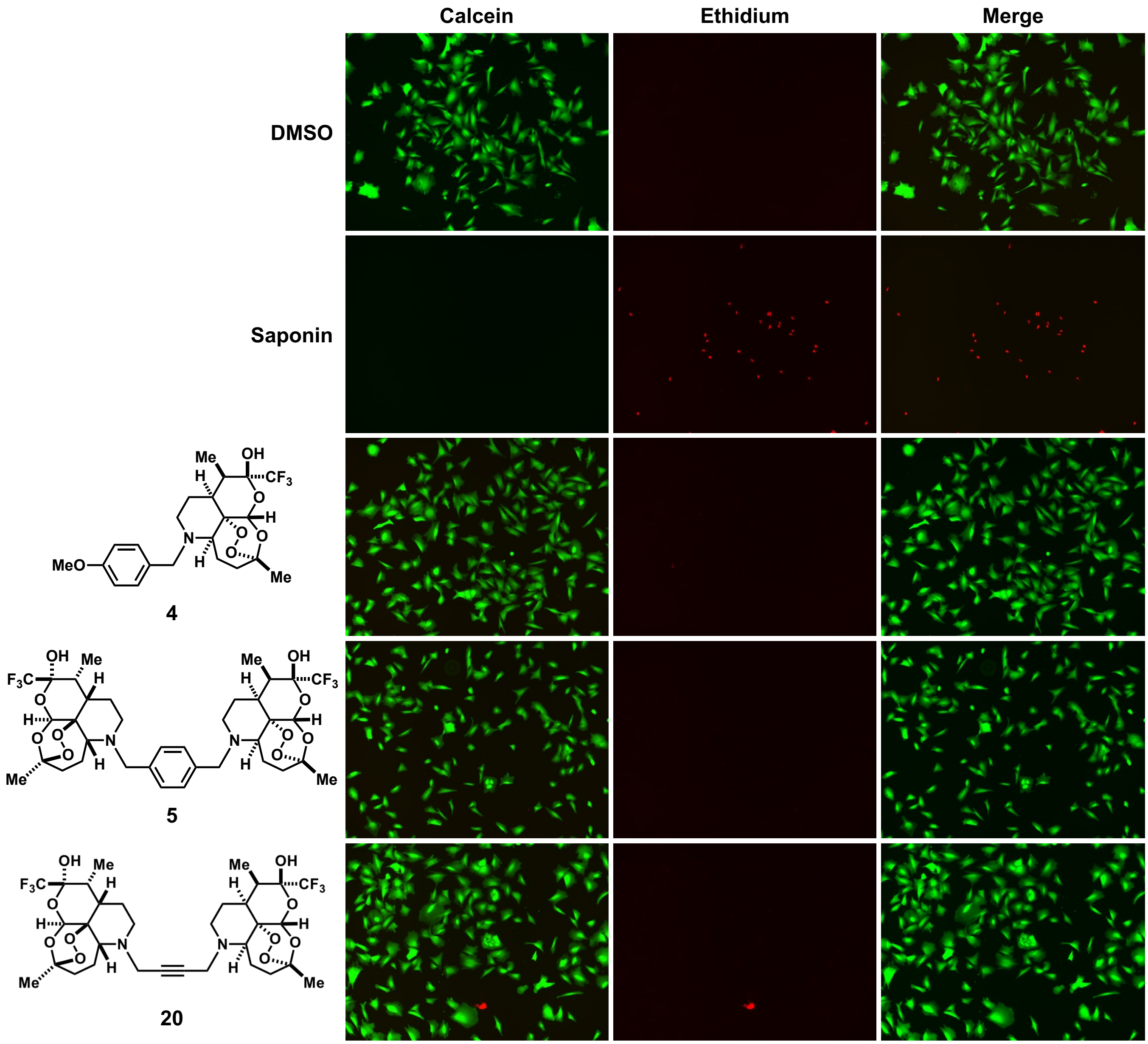

Figure S6. Live/dead staining of iPSC-derived qHSCs treated with 6-aza-artemisinins. Representative fluorescence images of iPSC-derived qHSCs treated with compounds **4**, **5**, or **20** at 500 nM for 72 h, followed by live/dead staining with Calcein AM and ethidium homodimer. Calcein-positive cells indicate viable cells, whereas ethidium-positive cells indicate membrane-compromised dead cells. DMSO-treated cells served as the vehicle control, whereas saponin-treated cells served as the positive control for membrane permeabilization.

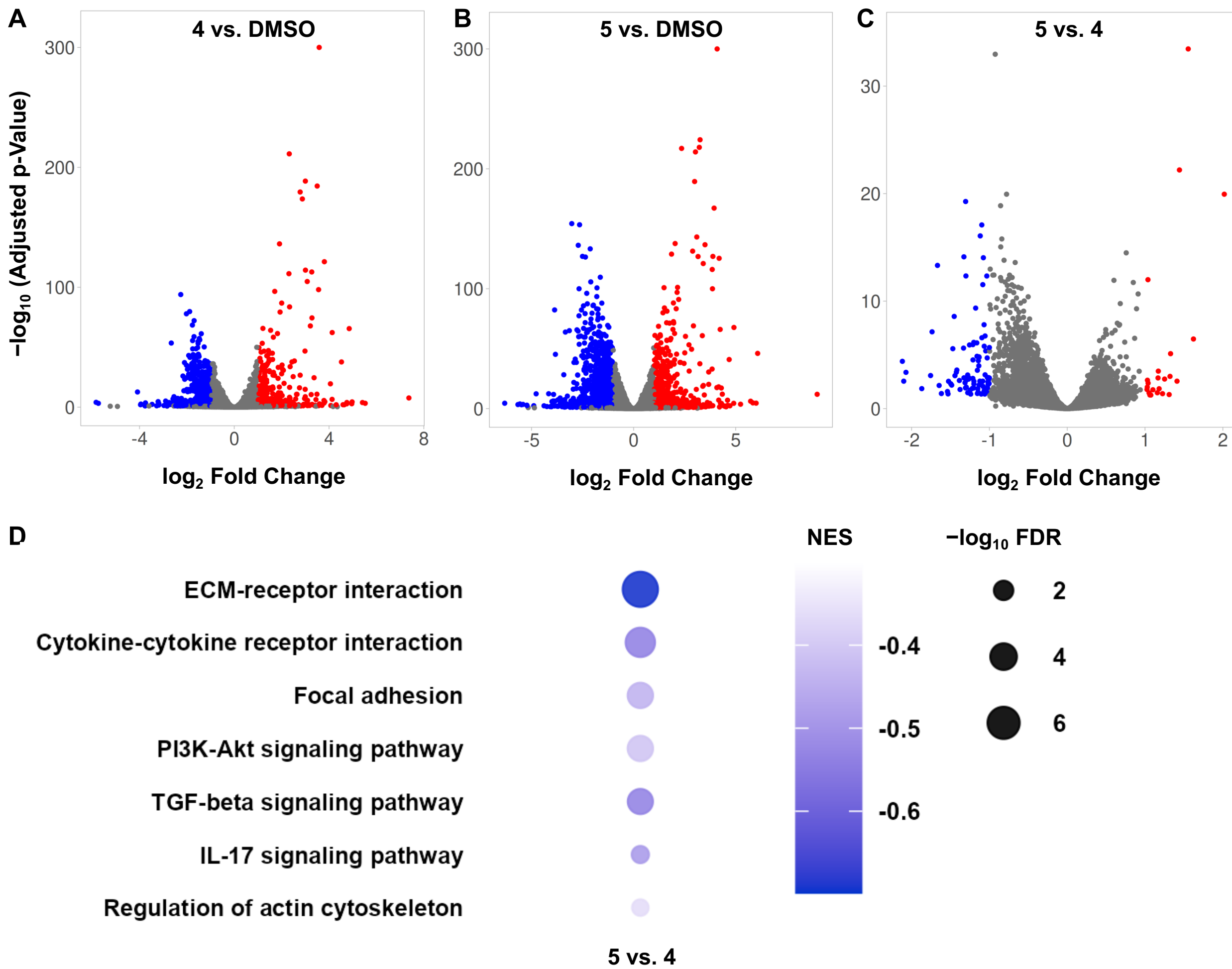

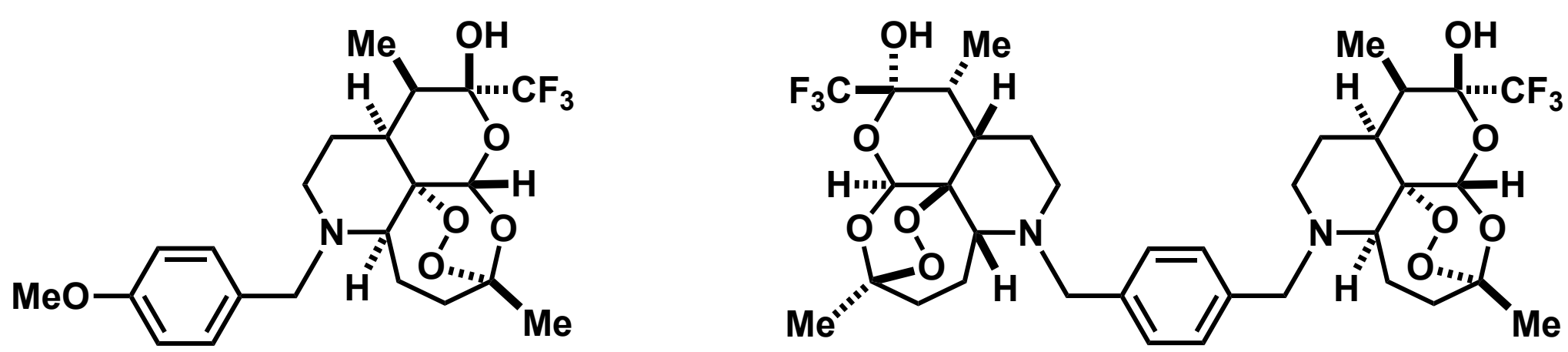

4 **5**

Figure S7. Differential gene expression and pathway enrichment analyses in iPSC-derived quiescent hepatic stellate cells. Volcano plots showing differentially expressed genes (DEGs) for (A) **4** vs DMSO, (B) **5** vs DMSO, and (C) **5** vs **4** comparisons. Red and blue dots indicate significantly up-regulated and down-regulated genes, respectively ($|\log_2(\text{fold change})| > 1$, adjusted $p < 0.05$). Complete lists of DEGs for each comparison are provided as a separate CSV file. (D) Bubble plot showing GSEA results highlighting fibrosis-related KEGG pathways that were significantly down-regulated in iPSC-derived quiescent hepatic stellate cells (qHSCs) treated with dimer **5** compared with monomer **4**. Color indicates normalized enrichment score (NES), and dot size represents $-\log_{10}$FDR.

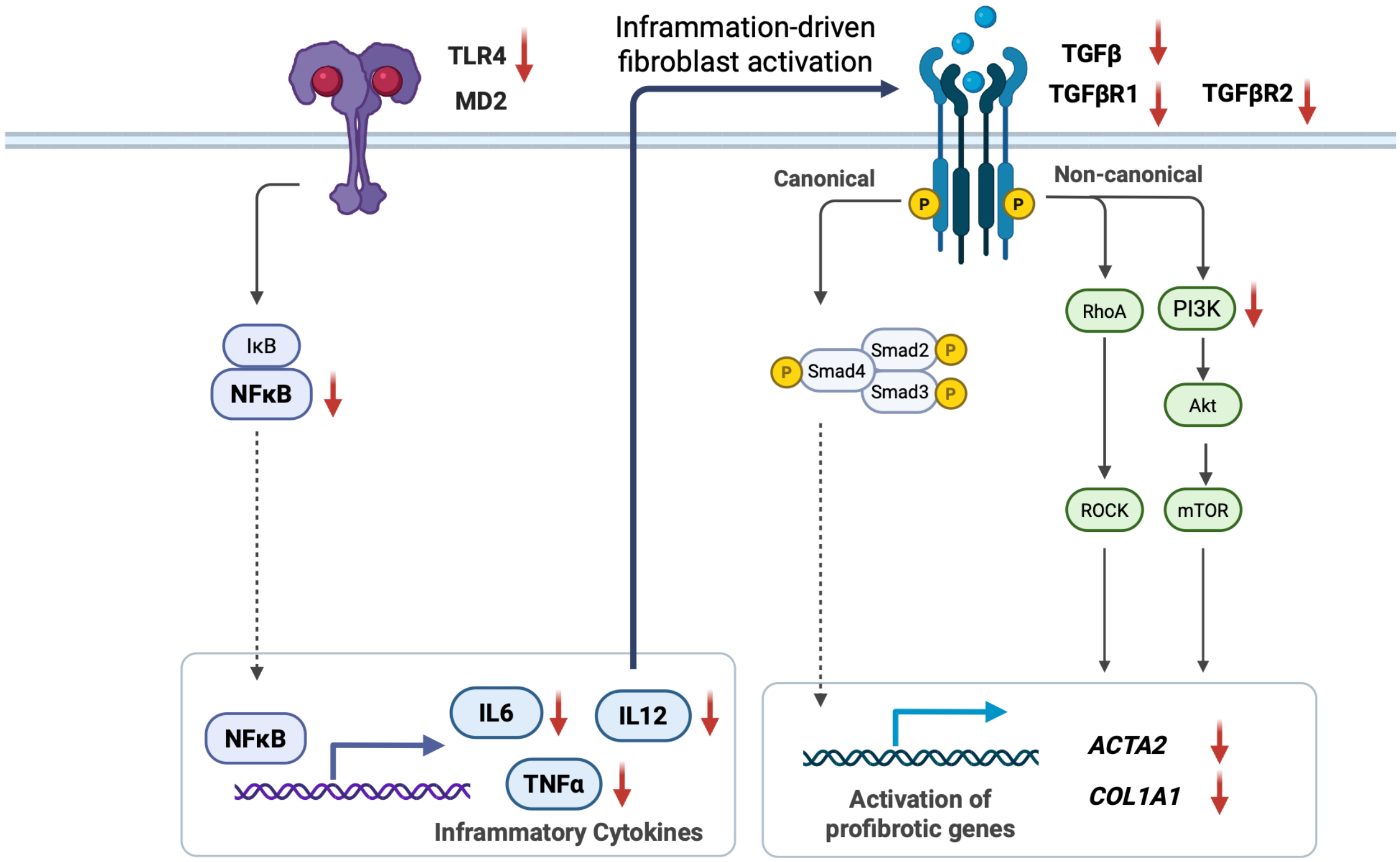

Figure S8. Schematic representation of inflammation-driven and TGF-β–dependent profibrotic signaling pathways relevant to fibroblast activation and fibrosis. Molecular components whose expression is reduced, or whose associated signaling programs are transcriptionally attenuated, upon treatment with 6-aza-artemisinins (**4** or **5**) are indicated by red downward arrows, as supported by RNA-seq and RT-qPCR analyses. Solid arrows denote established activating signaling relationships, whereas dashed arrows indicate nuclear translocation processes. The schematic highlights representative pathways directly relevant to fibroblast activation and fibrosis rather than providing a comprehensive map of all TGF-β–associated signaling networks. Detailed experimental procedures related to Figs. S8-14 are provided in Section 4.4.

We present KEGG pathway maps generated using Pathview to visualize transcriptional changes across individual pathway components in a data-driven manner (Figs. S9–S14). These pathway-resolved mappings serve as the primary reference for the simplified schematic summary shown in Fig. S8.

Within these KEGG/Pathview mappings, compound-induced transcriptional changes can be resolved at the level of individual pathway components, revealing coordinated modulation of inflammatory and profibrotic signaling networks by 6-aza-artemisinin monomer **4** and dimer **5**. Specifically, in the Toll-like receptor signaling pathway, Pathview mappings highlight down-regulation of nodes corresponding to *TLR4* and downstream NF-κB–dependent pro-inflammatory cytokine genes. In parallel, in the TGF-β signaling pathway, multiple nodes encompassing TGF-β, its receptors, and downstream non-canonical components such as RhoA exhibit down-regulation. Notably, comparison of the corresponding Pathview outputs further indicates that these pathway-level transcriptional changes are generally more pronounced upon treatment with dimer **5** than with monomer **4**, consistent with stronger transcriptional attenuation of profibrotic pathway components by the dimeric scaffold.

TGF-β signaling pathway (Figs. S9–S11): Consistent with the pathway-level trends summarized above, Pathview-based KEGG mapping of the TGF-β signaling pathway reveals compound-dependent suppression of key profibrotic nodes. Treatment with monomer **4** results in down-regulation of nodes encompassing TGF-β and selected downstream non-canonical components, including RHOA, indicating partial down-regulation of transcriptional components associated with the TGF-β pathway (Fig. S9). In contrast, dimer **5** induces a more pronounced and coordinated reduction across these pathway components relative to DMSO control (Fig. S10). Direct comparison of dimer **5** and monomer **4** further demonstrates stronger down-regulation of TGF-β–associated pathway components by the dimeric scaffold, as evidenced by down-regulation of TGF-β and its receptors *TGFBR1* and *TGFBR2* (Fig. S11). Together, these comparative mappings highlight differential transcriptional modulation of canonical and non-canonical TGF-β pathway components by monomeric and dimeric 6-aza-artemisinins.

TLR4/MD2 signaling pathway (Figs. S12–S14): Consistent with the pathway-level trends summarized above, Pathview-based KEGG mapping of the Toll-like receptor signaling pathway reveals compound-dependent suppression of inflammation-associated signaling nodes. Treatment with monomer **4** results in reduced expression of *TLR4*, the downstream transcription factor *NF-κB*, and multiple NF-κB–dependent pro-inflammatory cytokine genes, indicating attenuation of TLR4-mediated inflammatory signaling (Fig. S12). In contrast, dimer **5** induces a more pronounced and broader reduction across these pathway components relative to DMSO control, consistent with enhanced suppression of the TLR4–NF-κB inflammatory axis (Fig. S13). Direct comparison of dimer **5** and monomer **4** further demonstrates stronger down-regulation of *TLR4* and downstream pro-inflammatory cytokines, including *TNF*, *IL6*, and *IL12*, in cells treated with the dimeric scaffold (Fig. S14). Together, these comparative mappings highlight differential modulation of the TLR4/MD2–NF-κB inflammatory pathway by monomeric and dimeric 6-aza-artemisinins.

IL-17 signaling pathway (Fig. S7D): In addition to the TGF-β and Toll-like receptor pathways, GSEA-based pathway enrichment analysis further identified significant down-regulation of the IL-17 signaling pathway in qHSCs treated with dimer **5** compared with monomer **4** (Fig. S7D). IL-17 signaling is known to functionally intersect with the TLR4–NF-κB axis to amplify inflammatory gene expression in fibroblast-related contexts. The observed suppression of IL-17–associated transcriptional programs is therefore consistent with the coordinated attenuation of inflammation-driven signaling pathways highlighted in the subsequent Pathview-based analyses (Figs. S12–S14).

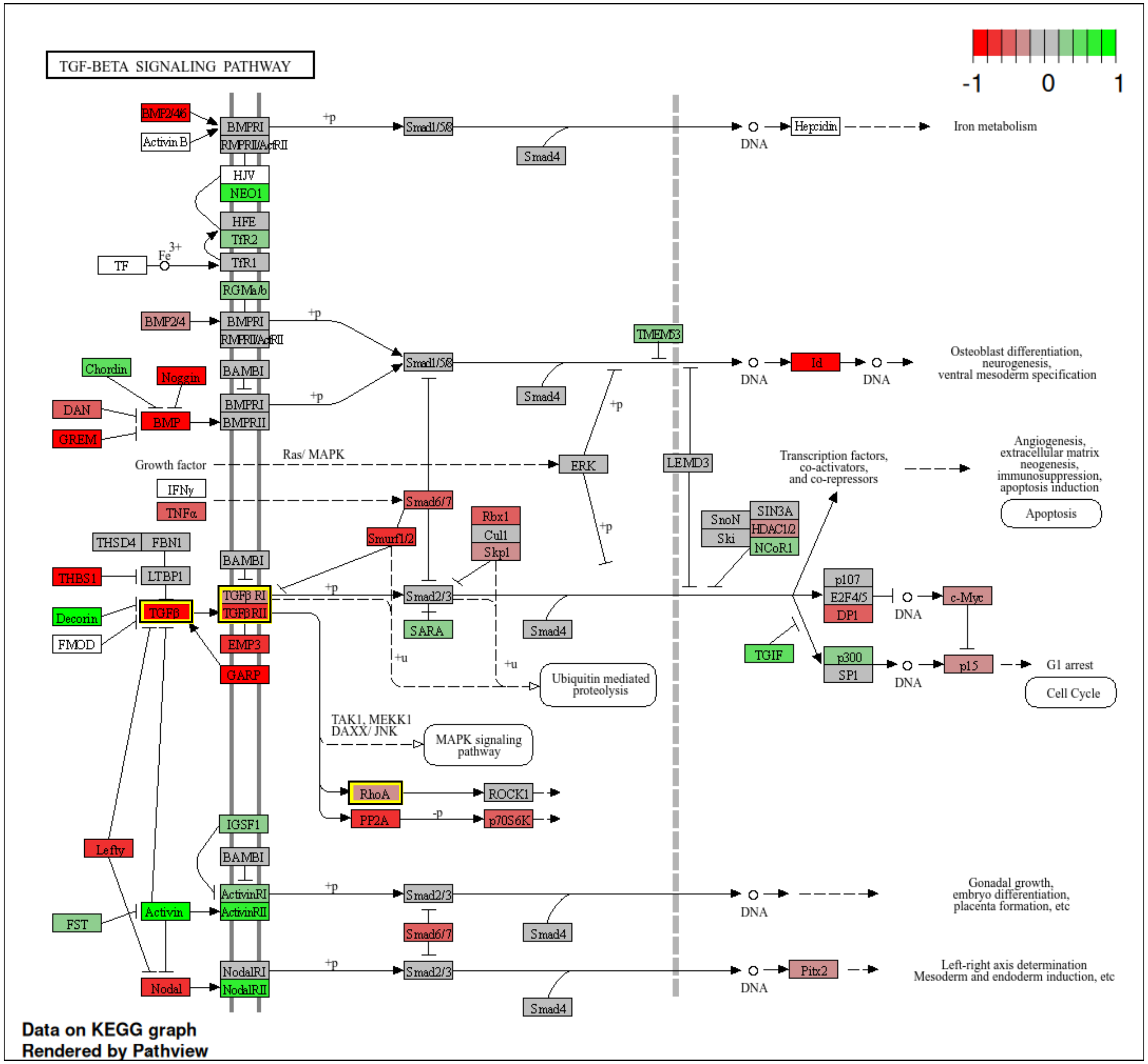

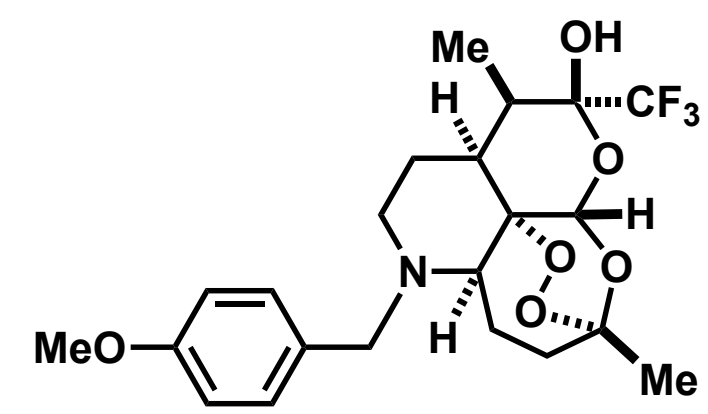

4

Figure S9. Pathview-based KEGG pathway mapping illustrating transcriptional changes in the TGF-β signaling pathway in iPSC-derived qHSCs treated with monomer 4 relative to DMSO control. Down-regulation is observed for TGF-β and selected downstream non-canonical components, including RhoA. Nodes showing markedly reduced gene expression are highlighted in yellow.

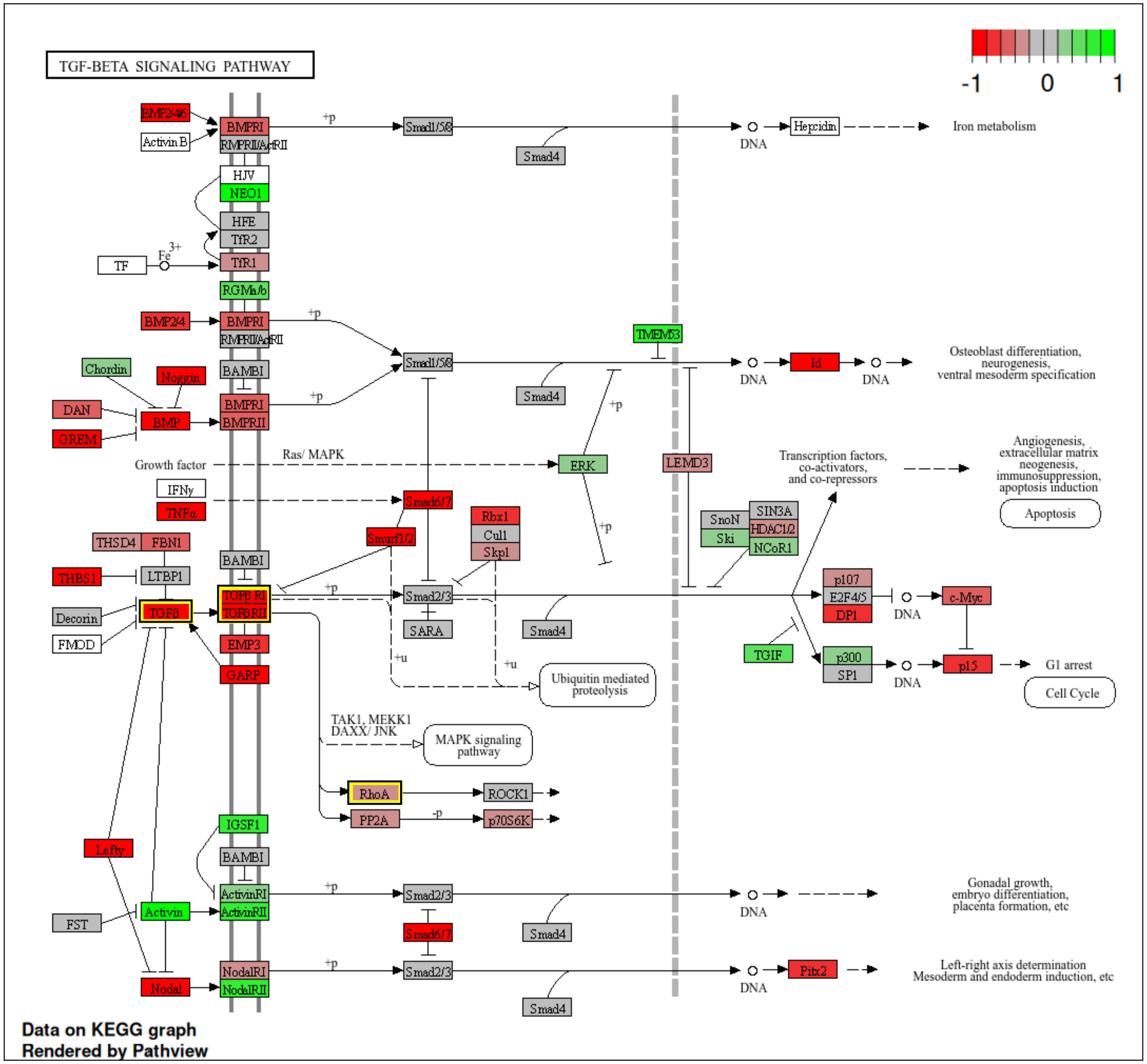

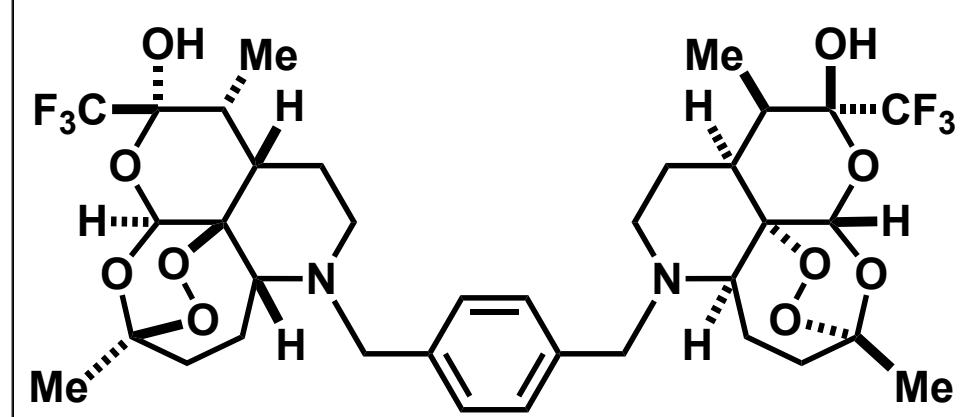

5

Figure S10. Pathview-based KEGG pathway mapping illustrating transcriptional changes in the TGF-β signaling pathway in iPSC-derived qHSCs treated with dimer 5 relative to DMSO control. Down-regulation is observed for TGF-β and selected downstream non-canonical components, including RhoA. Nodes showing markedly reduced gene expression are highlighted in yellow.

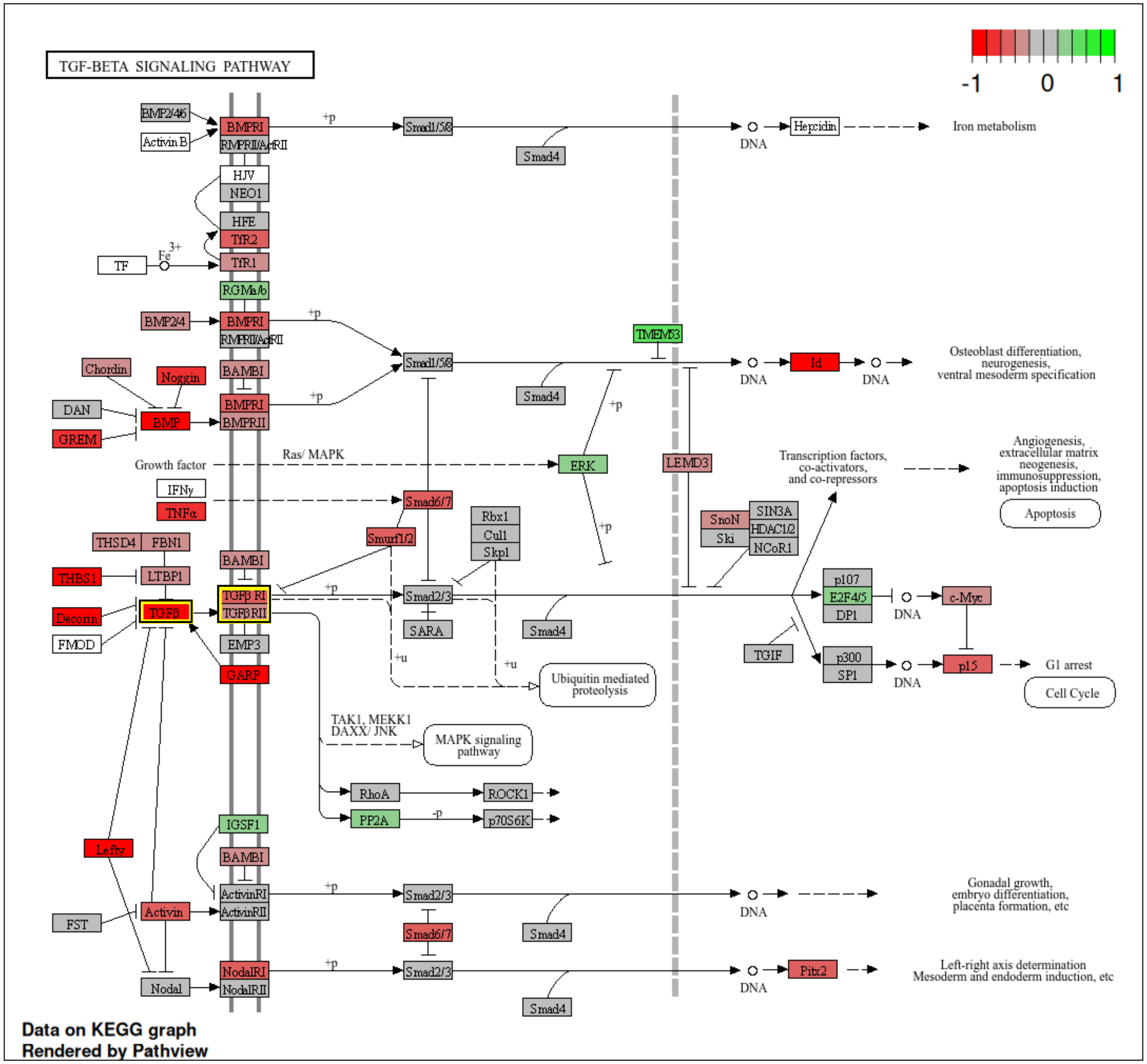

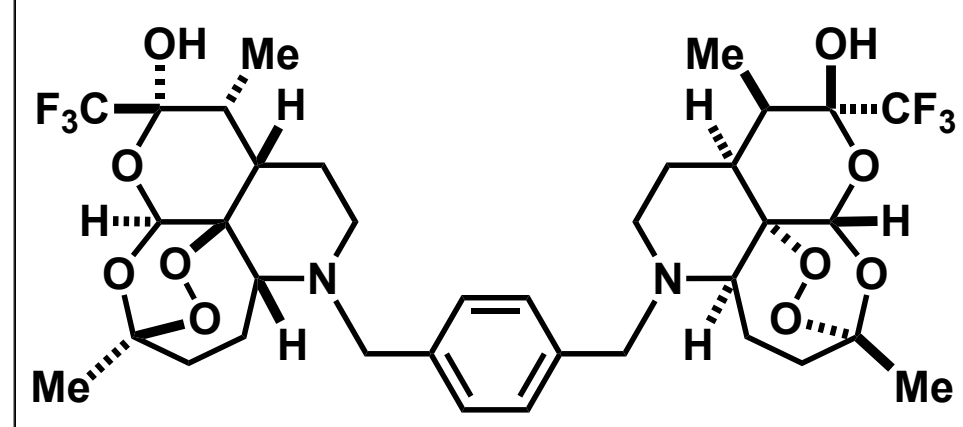

5

vs.

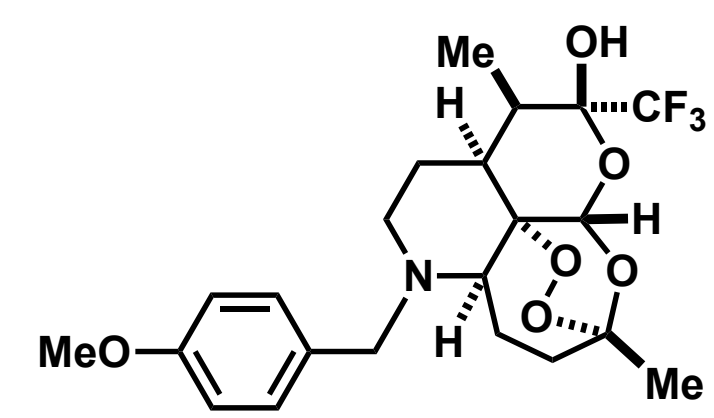

4

Figure S11. Pathview-based KEGG pathway mapping illustrating transcriptional changes in the TGF-β signaling pathway in iPSC-derived qHSCs treated with dimer 5 relative to monomer 4. Compared with monomer **4**, down-regulation is observed for TGF-β and its receptors TGFBR1 and TGFBR2 in cells treated with dimer **5**. Nodes showing markedly reduced gene expression are highlighted in yellow.

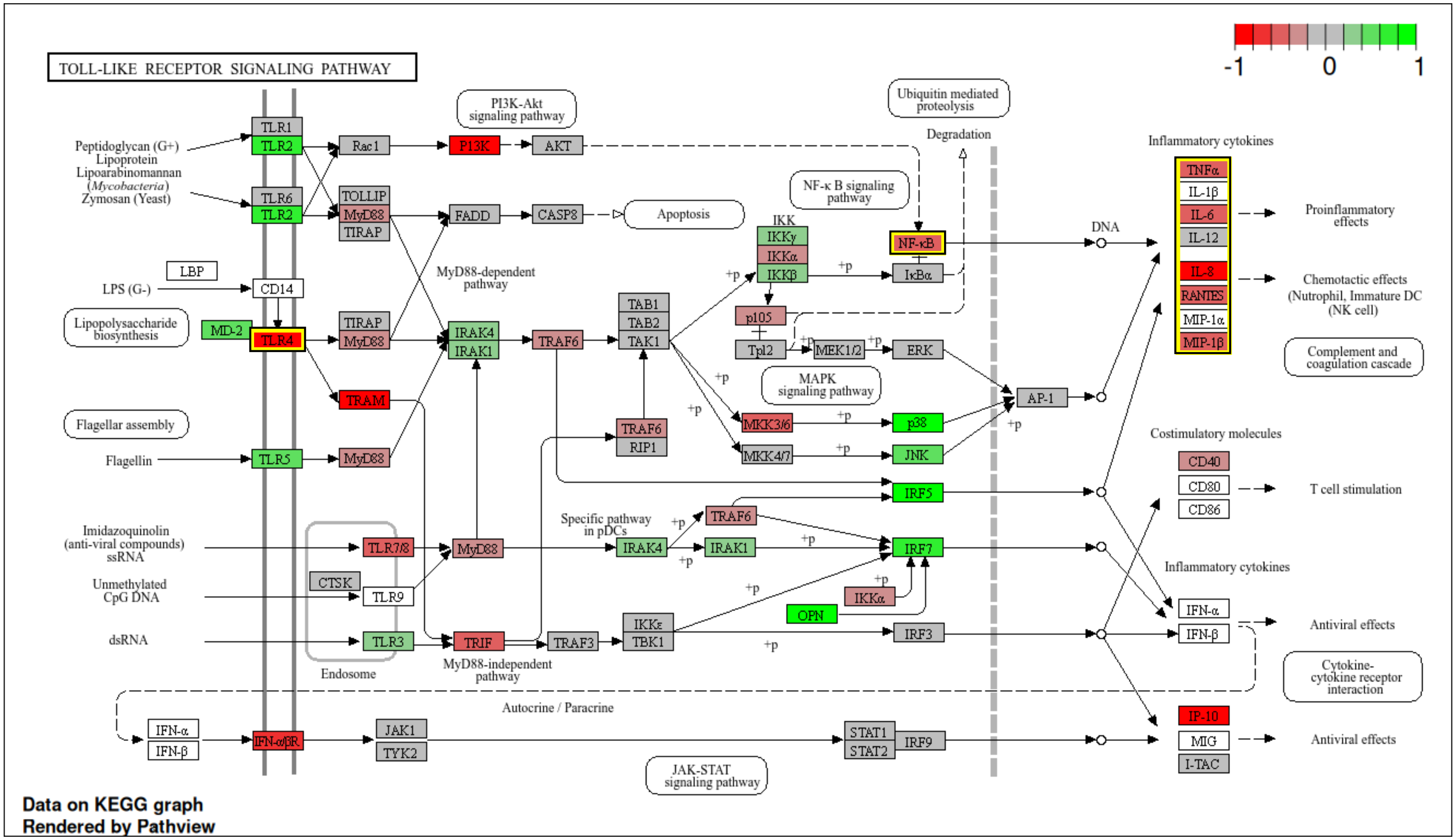

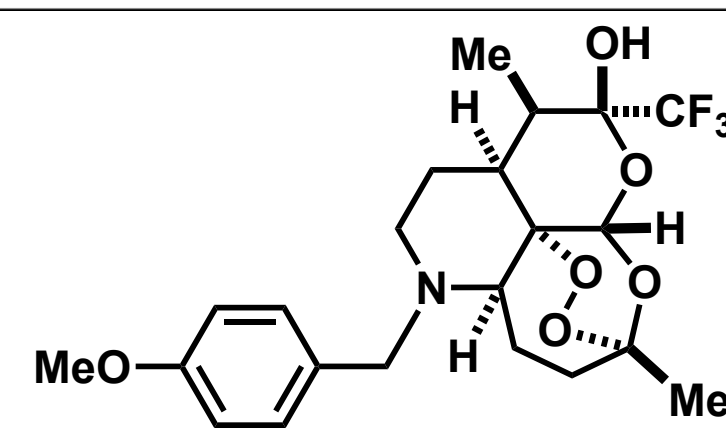

4

Figure S12. Pathview-based KEGG pathway mapping illustrating transcriptional changes in the Toll-like receptor signaling pathway in iPSC-derived qHSCs treated with monomer 4 relative to DMSO control. Reduced expression is observed for *TLR4*, the downstream transcription factor *NF-κB*, and a broad set of downstream NF-κB–dependent pro-inflammatory cytokine genes. Nodes showing markedly reduced gene expression are highlighted in yellow.

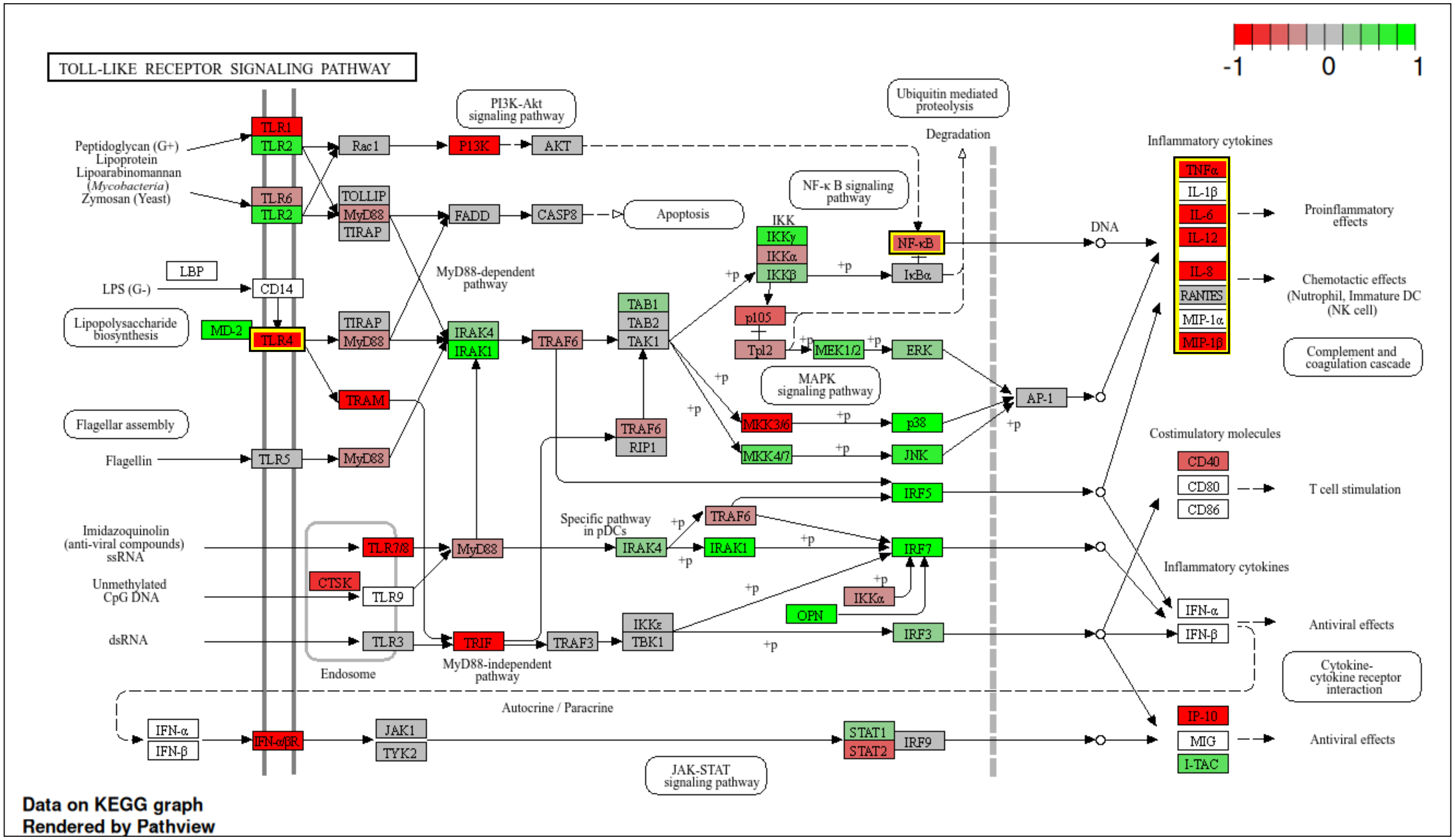

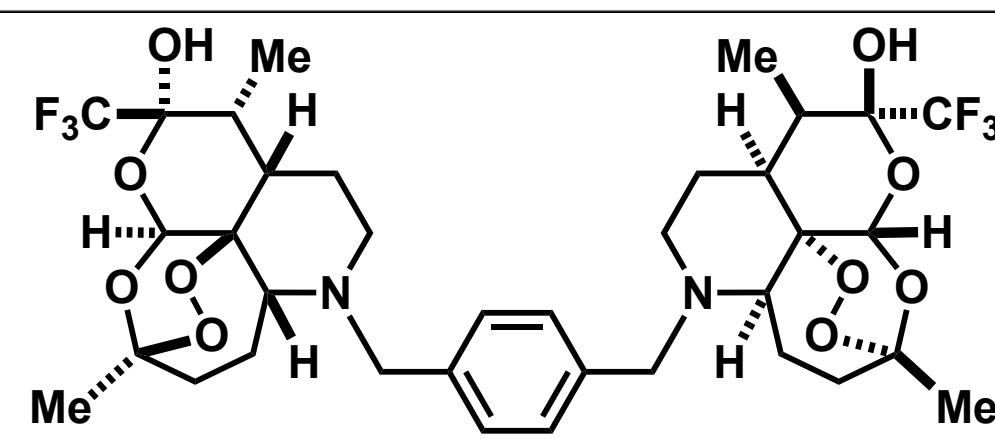

5

Figure S13. Pathview-based KEGG pathway mapping illustrating transcriptional changes in the Toll-like receptor signaling pathway in iPSC-derived qHSCs treated with dimer 5 relative to DMSO control. Reduced expression is observed for *TLR4*, the downstream transcription factor *NF-κB,* and a broad set of downstream NF-κB–dependent pro-inflammatory cytokine genes. Nodes showing markedly reduced gene expression are highlighted in yellow.

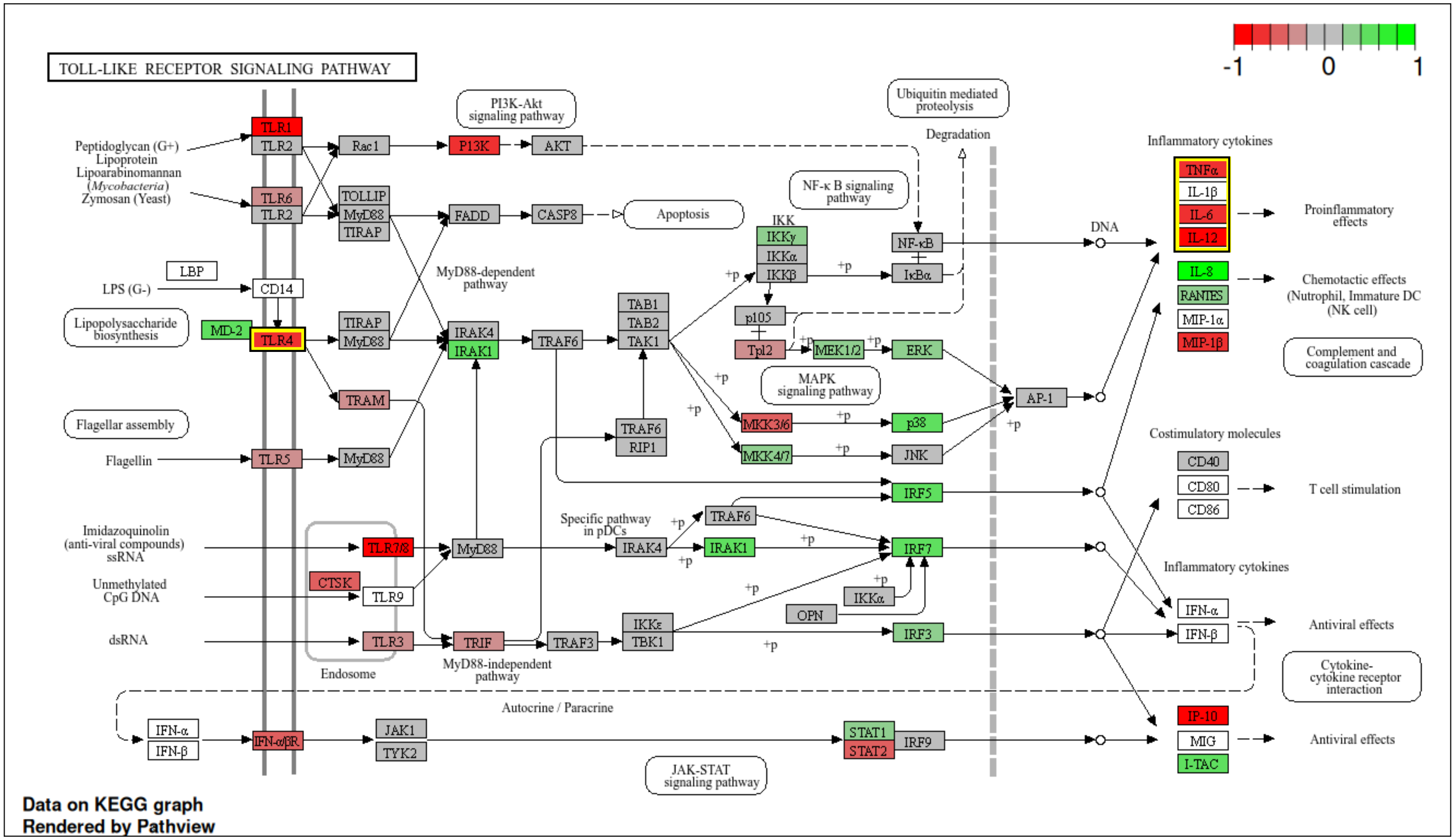

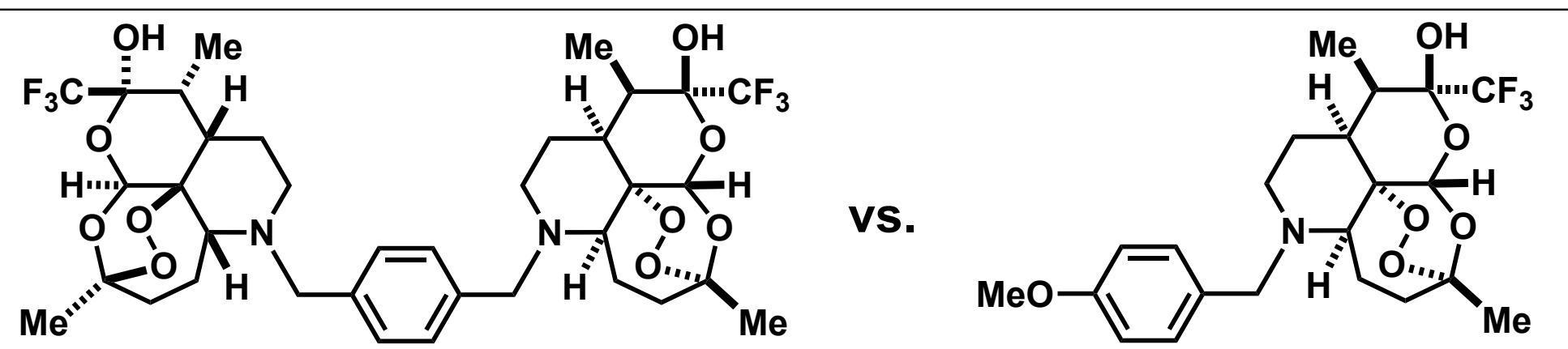

5 **4**

Figure S14. Pathview-based KEGG pathway mapping illustrating transcriptional changes in the Toll-like receptor signaling pathway in iPSC-derived qHSCs treated with dimer 5 relative to monomer 4. Compared with monomer **4**, reduced expression is observed for *TLR4* and downstream pro-inflammatory cytokine genes, including *TNF*, *IL6*, and *IL12*, in cells treated with dimer **5**. Nodes showing markedly reduced gene expression are highlighted in yellow.

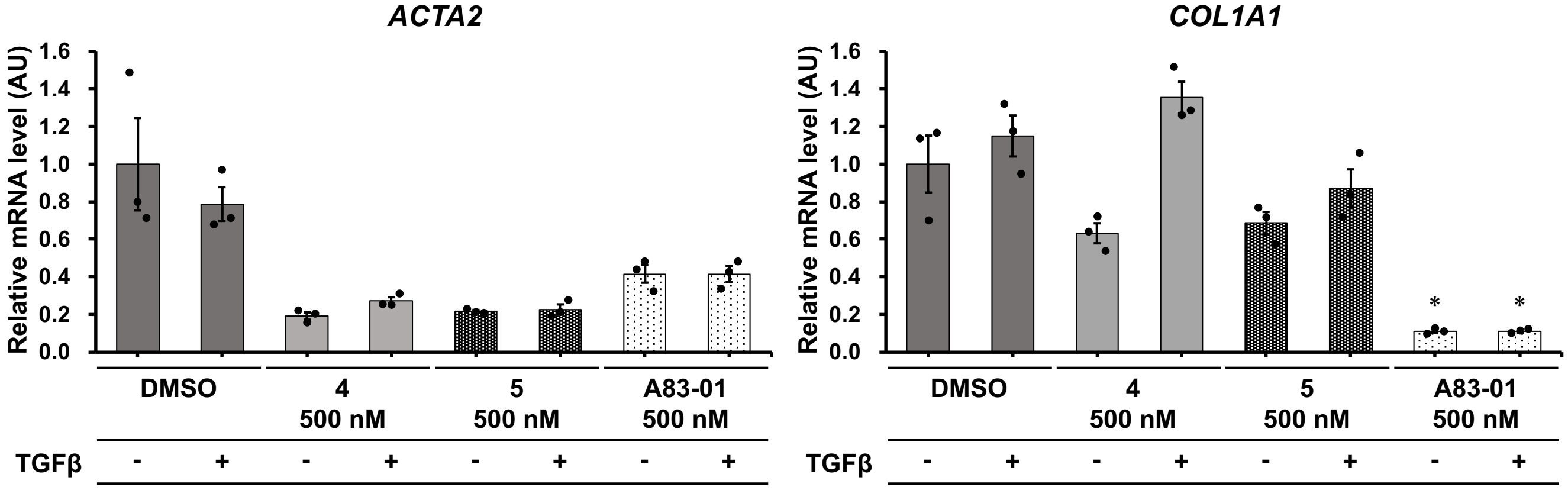

Figure S15. Effects of 6-aza-artemisinins on fibrogenic marker expression under exogenous TGF-β stimulation in iPSC-derived qHSCs. qRT-PCR analysis of fibrosis-associated genes (*ACTA2* and *COL1A1*) in iPSC-derived qHSCs pretreated with **4**, **5**, or A83-01 at 500 nM for 24 h, followed by stimulation with TGF-β at 10 ng/mL for 24 h. Gene expression levels were normalized to *GAPDH* and are shown relative to the DMSO-treated control without TGF-β stimulation. Data are presented as mean ± s.e. (n = 3 independent culture wells from the same differentiation batch of iPSC-derived qHSCs). Each dot represents one independent well and asterisks above individual bars indicate statistical significance compared with the DMSO control without TGF-β stimulation. * $p<0.05$, ** $p<0.01$, *** $p<0.001$.

Because the RNA-seq analysis indicated suppression of TGF-β–associated profibrotic transcriptional programs by **4** and **5**, we examined whether these compounds suppress fibrogenic marker expression under exogenous TGF-β stimulation. In DMSO-treated iPSC-derived qHSCs, TGF-β caused only modest additional changes in *ACTA2* and *COL1A1* expression, suggesting that the cells had already undergone substantial culture-associated activation under the present conditions. In the absence of exogenous TGF-β, **4**, and **5** reduced the expression of *ACTA2* and *COL1A1*. Upon TGF-β stimulation, *ACTA2* expression remained suppressed in cells treated with these 6-aza-artemisinins, whereas *COL1A1* expression was partially increased, particularly in cells treated with **4** or **5**. In contrast, A83-01, a reported inhibitor of ALK5/TGF-β type I receptor signaling,[1] strongly suppressed *COL1A1* expression regardless of TGF-β stimulation, consistent with its role as a direct ALK5/TGF-β receptor inhibitor. These results suggest that 6-aza-artemisinins attenuate the activated fibrogenic state of iPSC-derived qHSCs but do not fully suppress direct TGF-β-driven *COL1A1* induction under these experimental conditions.

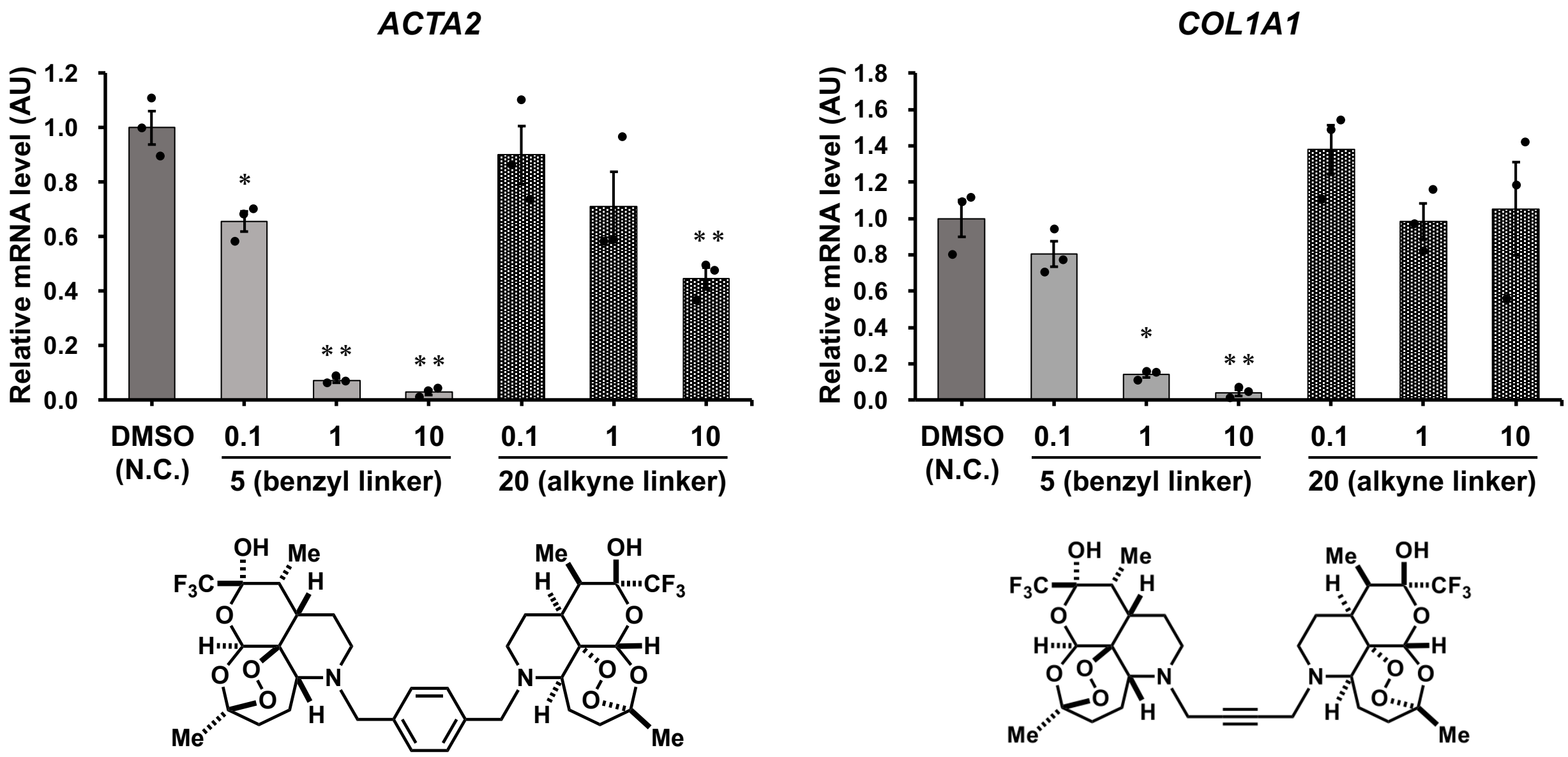

5 20

Figure S16. Linker-dependent antifibrotic activity of 6-aza-artemisinin dimers in systemic sclerosis (SSc) patient-derived dermal fibroblasts. qRT-PCR analysis of fibrosis-associated genes (*ACTA2* and *COL1A1*) in SSc patient-derived dermal fibroblasts treated with **5** or **20** at 0.1, 1, and 10 μM for 72 h. Gene expression levels were normalized to *GAPDH* and are shown relative to the DMSO-treated negative control. Data are presented as mean ± s.e. (n = 3 independent culture wells from the same patient-derived fibroblast line). Each dot represents one independent well and asterisks above individual bars indicate statistical significance compared with the DMSO control. * p<0.05, ** p<0.01, *** p<0.001.

Table S3. Microsomal intrinsic clearance of selected artemisinin analogs.

Compound	Clearance (μL/min/mg protein)	
	Mouse	Human
**4**	574	83
**16**	583	167
**5**	**23**	**67**
**20**	69	111
**artemisinin (1)**	145	59
**artesunate (2)**	552	293

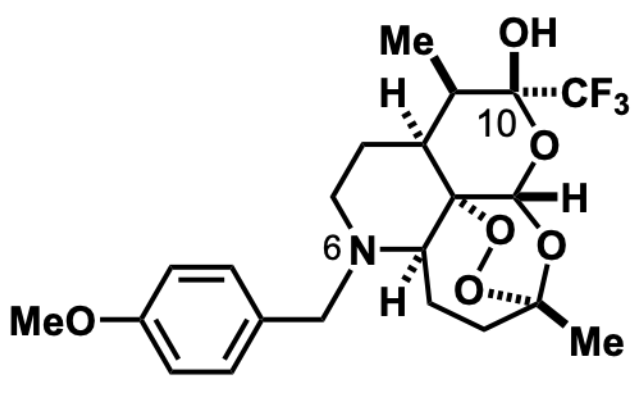

4

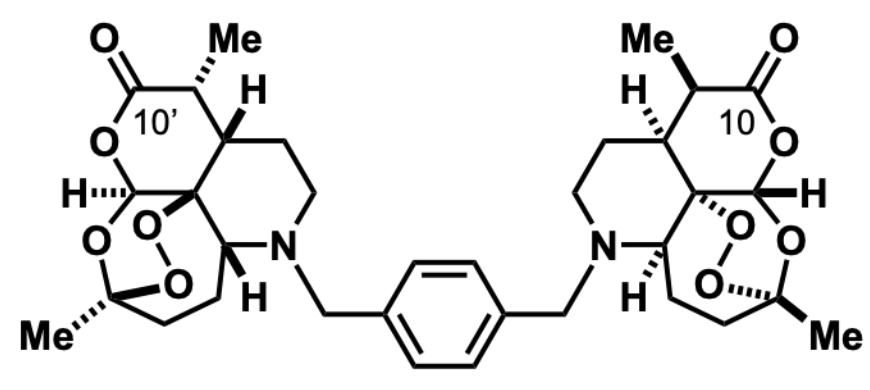

16

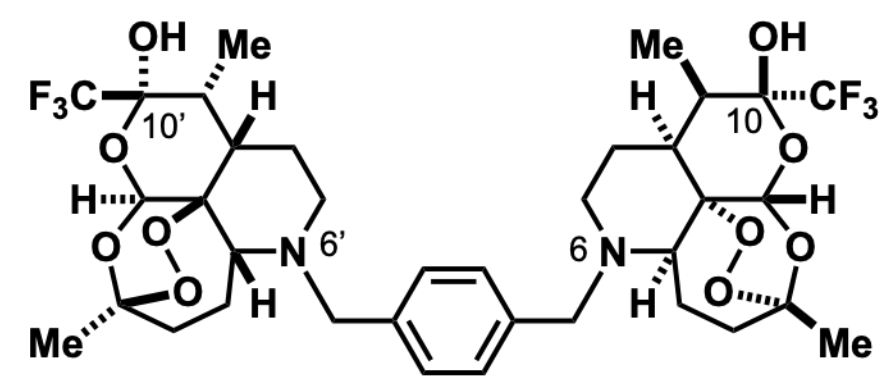

5

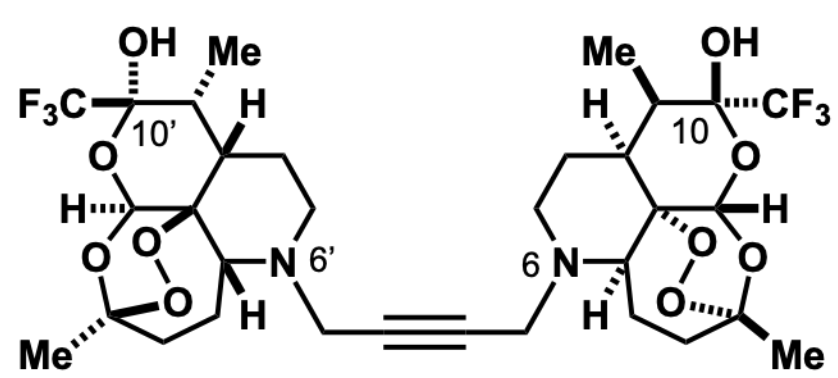

20

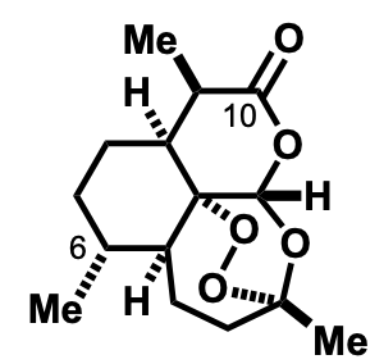

artemisinin (**1**)

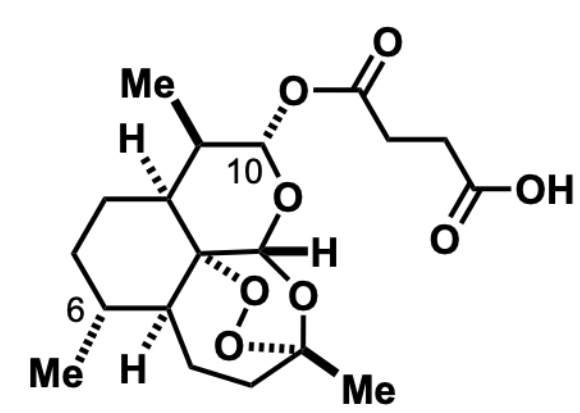

artesunate (**2**)

Microsomal metabolic stability analysis revealed species-dependent but informative trends among the artemisinin analogs. In mouse liver microsomes, compounds **4** and **16** exhibited high intrinsic clearance, whereas the C10/C10′-trifluoromethylated N6‑N6′ dimer **5** showed markedly reduced clearance. The alkyne-linked dimer **20** displayed intermediate clearance, indicating that dimerization alone was insufficient to achieve the metabolic stability observed for **5**. In human liver microsomes, the N6‑N6′ dimer **5** again exhibited the lowest intrinsic clearance among the 6-aza-artemisinin analogs evaluated, whereas compounds **16** and **20** showed substantially higher clearance values. Compound **4** showed low clearance than compounds **16** and **20** but remained less metabolically stable than **5**. Notably, the dimer **5** exhibited approximately 25-fold lower intrinsic clearance than either **4** or **16** in mouse liver microsomes and showed lower clearance than both artemisinin (**1**) and artesunate (**2**) under the same assay conditions. Collectively, these results demonstrate that the combination of C10/C10′ trifluoromethylation and the N6‑N6′ benzyl-linked dimeric architecture provides the greatest improvement in microsomal metabolic stability, with compound **5** exhibiting the highest metabolic stability among all compounds evaluated.

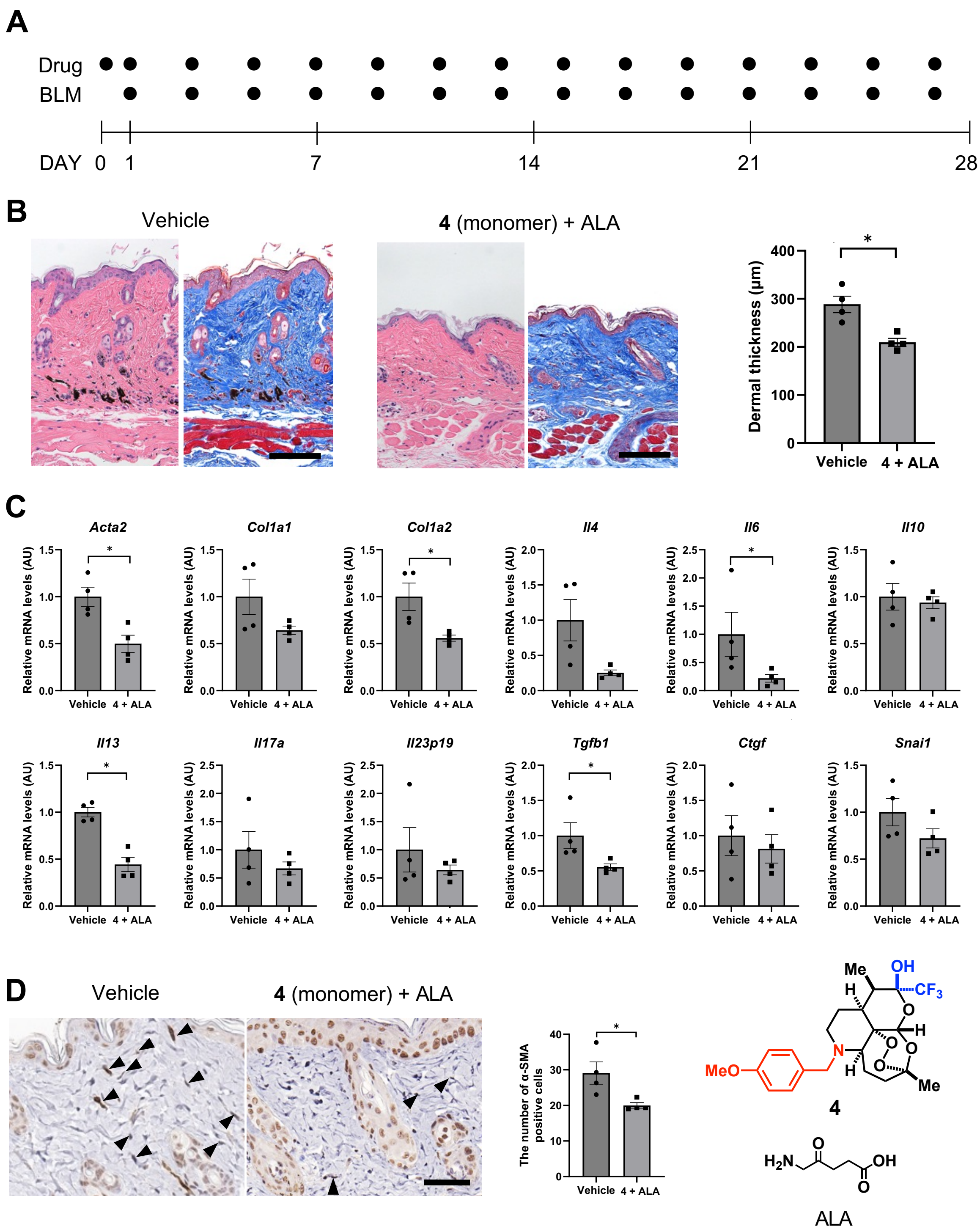

Figure S17. Prophylactic efficacy of 6-aza-artemisinin 4 (monomer) combined with ALA in the BLM-induced skin fibrosis model. (A) Treatment schedule of the prophylactic model. (B) Representative histological images of dermal sections from BLM-treated mice receiving vehicle or **4** (monomer, 5 mg/kg) + ALA. Scale bar, 100 μm. (C) qRT-PCR analysis of fibrosis- and cytokine-related genes in skin tissue following treatment with vehicle or **4** + ALA. (D) Immunohistochemical staining for α-SMA(ACTA2) in the skin sections from vehicle- or **4** + ALA treated mice. Scale bar, 50 μm. Abbreviations: BLM, bleomycin; ALA, 5-aminolevulinic acid. Data are presented as mean ± s.e. (n = 4). Gene expression levels were normalized to *Gapdh*. * $p<0.05$, ** $p<0.01$, *** $p<0.001$.

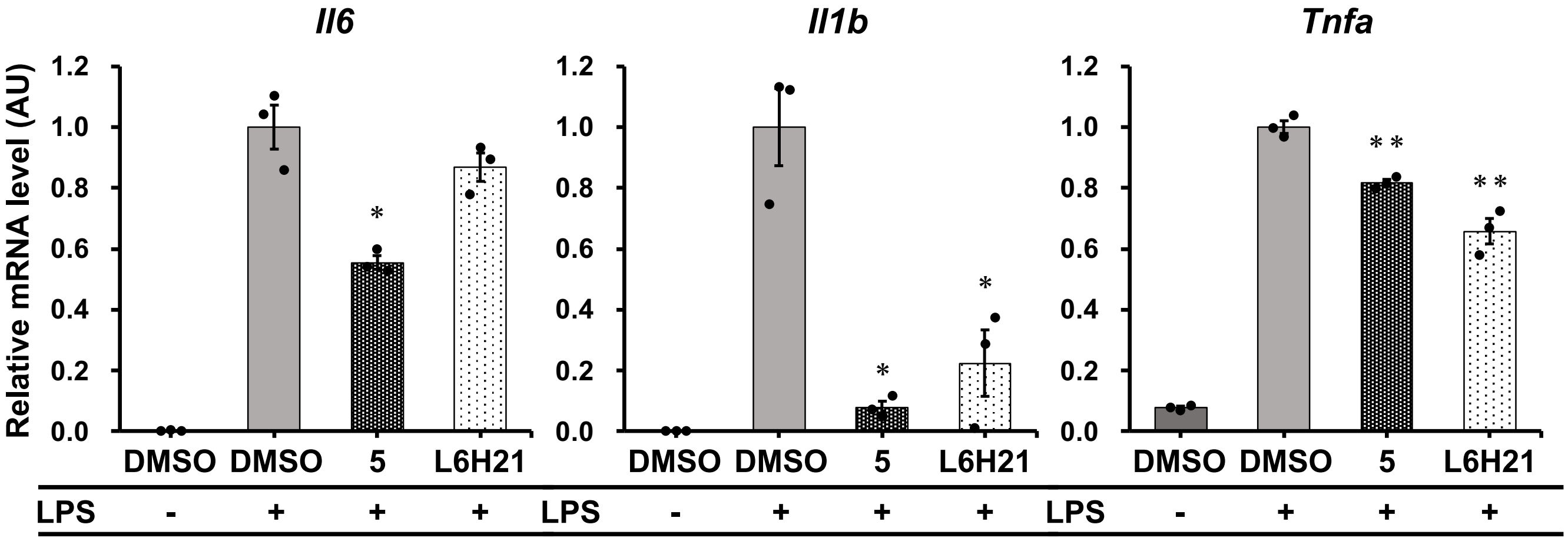

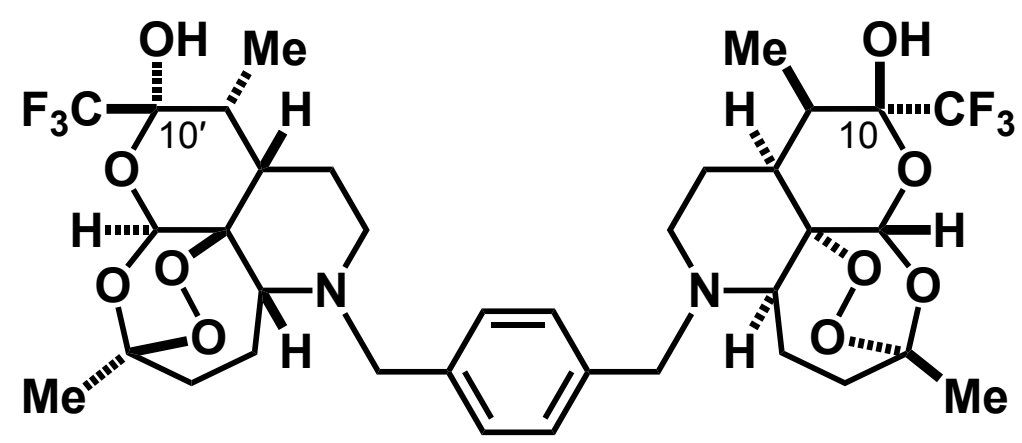

5

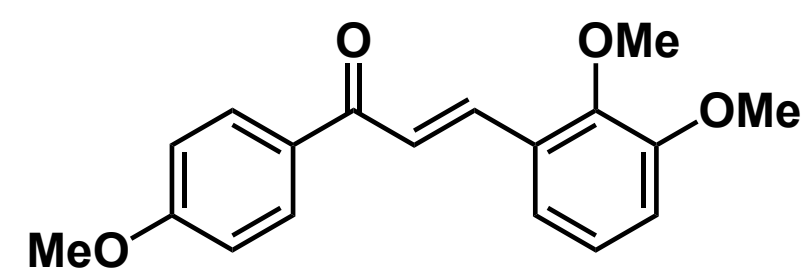

L6H21

Figure S18. Suppression of lipopolysaccharide (LPS)-induced proinflammatory cytokine expression by dimer 5 in RAW264.7 murine macrophage cells. qRT-qPCR analysis of proinflammatory cytokine genes (*Il6*, *Il1b*, and *Tnfa*) in RAW264.7 cells pretreated with dimer **5** or the reported MD2 inhibitor L6H21 at 10 μM for 1 h, followed by stimulation with LPS (100 ng/mL) for an additional 6 h. DMSO-treated cells without LPS stimulation served as the unstimulated control, whereas DMSO-treated cells with LPS stimulation served as the LPS-stimulated vehicle control. L6H21 was included as a positive control inhibitor. Gene expression levels were normalized to *Gapdh* and are shown relative to the LPS-stimulated vehicle control. Data are presented as mean ± s.e. (n = 3 independent culture wells). Each dot represents one independent well, and asterisks above individual bars indicate statistical significance compared with the LPS-stimulated vehicle control. *$p < 0.05$, **$p < 0.01$, ***$p < 0.001$.

To examine whether dimer **5** suppresses LPS-induced inflammatory responses, its effects on the expression of the proinflammatory cytokine genes *Il6*, *Il1b*, and *Tnfa* were evaluated in LPS-stimulated RAW264.7 cells by RT-qPCR. L6H21,[2] a reported inhibitor of MD2-mediated LPS signaling, was included as a mechanistic reference compound. LPS stimulation markedly increased the expression of all three genes relative to the unstimulated control. Pretreatment with dimer **5** significantly reduced the LPS-induced expression of *Il6*, *Il1b*, and *Tnfa*. Under the same conditions, L6H21 significantly suppressed *Il1b* and *Tnfa* expression and showed a trend toward reduced *Il6* expression. Thus, dimer **5** exhibited an inhibitory profile that broadly overlapped with that of the reported MD2 inhibitor. These findings suggest that dimer **5** suppresses LPS-responsive inflammatory signaling, consistent with modulation of MD2-dependent LPS signaling.

Table S4. List of quantitative PCR primers for mouse and human genes.

		Left Primer	Right Primer
**Mouse** (For Fig. S17)	***Acta2***	ACATCAAGGAGAAGCTGTGC	AGCGTTCGTTTCCAATGGTG
	Col1a1	TCAGCTGCATACACAATGGC	ATTGCATTGCACGTCATCGC
	Col1a2	AGGAAATGGCAACTCAGCTC	ACGCAATGCTGTTCTTGCAG
	Ctgf	AGTGTGCACTGCCAAAGATG	TTTGCAGCTGCTTTGGAAGG
	Gapdh	CGTGTTCCTACCCCCAATGT	TGTCATCATACTTGGCAGGTTTCT
	Il4	ACAGCAACGAAGAACACCAC	TCGAAAAGCCCGAAAGAGTC
	Il6	ACAAAGCCAGAGTCCTTCAGAG	TTGGATGGTCTTGGTCCTTAGC
	Il10	GCAGGACTTTAAGGGTTACTTGG	GCTCCACTGCCTTGCTCTTA
	Il13	CACACAAGACCAGACTCCCC	CCAGGGATGGTCTCTCCTCA
	Il17a	AAAGCTCAGCGTGTCCAAAC	TTCTGGAGCTCACTTTTGCG
	Il23p19	TGCCAACAGCTTAAGGATGC	GCCCTTCACGCAAAACAAAC
	Ifng	AACTGGCAAAAGGATGGTGAC	GACCTGTGGGTTGTTGACCT
	Snai1	TGAAGATGCACATCCGAAGC	AATGGCTTCTCACCAGTGTG
	Tgfb1	TGCTCGCTTTGTACAACAGC	TCATAGATGGCGTTGTTGCG
**Mouse** (For Fig. S18)	***Gapdh***	TGTGTCCGTCGTGGATCTGA	TTGCTGTTGAAGTCGCAGGAG
	Il1b	AGTTGACGGACCCCAAAA	AGCTGGATGCTCTCATCAGG
	Il6	CACGGCCTTCCCTACTTCAC	TGCAAGTGCATCATCGTTGT
	Tnfa	TATGGCTCAGGGTCCAACTC	GGAAAGCCCATTTGAGTCCT
**Human**	***ACTA2***	CAGCCAAGCACTGTCAGG	CCAGAGCCATTGTCACACAC
	COL1A1	AAGAGGAAGGCCAAGTCGAG	CACACGTCTCGGTCATGGTA
	COL1A2	GGCCCTCAAGGTTTCCAAGG	CACCCTGTGGTCCAACAACTC
	COL3A1	AGGGGAGCTGGCTACTTCTC	AGGACTGACCAAGATGGGAA
	GAPDH	AGGTGAAGGTCGGAGTCAAC	CCATGTAGTTGAGGTCAATGAAG

2. Organic Synthesis

2.1 General Considerations

All reagents and solvents were purchased from Sigma-Aldrich, Tokyo Chemical Industry (TCI), Kanto Chemical, and FUJIFILM Wako Pure Chemical Corporation, and used as received unless otherwise noted. The synthetic intermediate **11** was prepared according to an established protocol developed in our laboratory.[3]

All the reactions were performed under a nitrogen atmosphere unless otherwise specified. All the reactions requiring heating were conducted in a heating mantle, and reaction temperatures are reported as the temperature of the heat transfer medium surrounding the vessel. Reactions were monitored by thin layer chromatography using Merck Millipore TLC silica gel F254 plates (0.21– 0.27 mm), which were visualized using UV light, PMS stain, *p*-anisaldehyde stain, or ninhydrin stain. Flash column chromatography was performed using Wakosil® 60 (38–63 μm). The medium-pressure liquid chromatography (MPLC) purifications were performed on a Biotage® Isolera or Biotage® Selekt.

NMR spectra were recorded on a JEOL ECS 400 (^{1}H/400 MHz, ^{13}C{^{1}H}/100 MHz) spectrometer. Chemical shifts were quoted in parts per million (ppm) from chloroform as an internal standard of 7.26 and 77.16 for ^{1}H and ^{13}C NMR, respectively. Data for ^{1}H NMR were reported as follows: chemical shift (number of hydrogens, multiplicity, coupling constant). Multiplicity is abbreviated as follows: s (singlet), d (doublet), t (triplet), q (quartet), m (multiplet), br (broad). ESI-Mass spectra were recorded on a Bruker micrOTOF-II. Single crystal X-ray diffraction data were collected using a Bruker D8 VENTURE diffractometer or the BL41XU beamline at the SPring-8 synchrotron radiation facility.

2.2 Ozone oxidation to form 6-aza-artemisinin **13** [3]

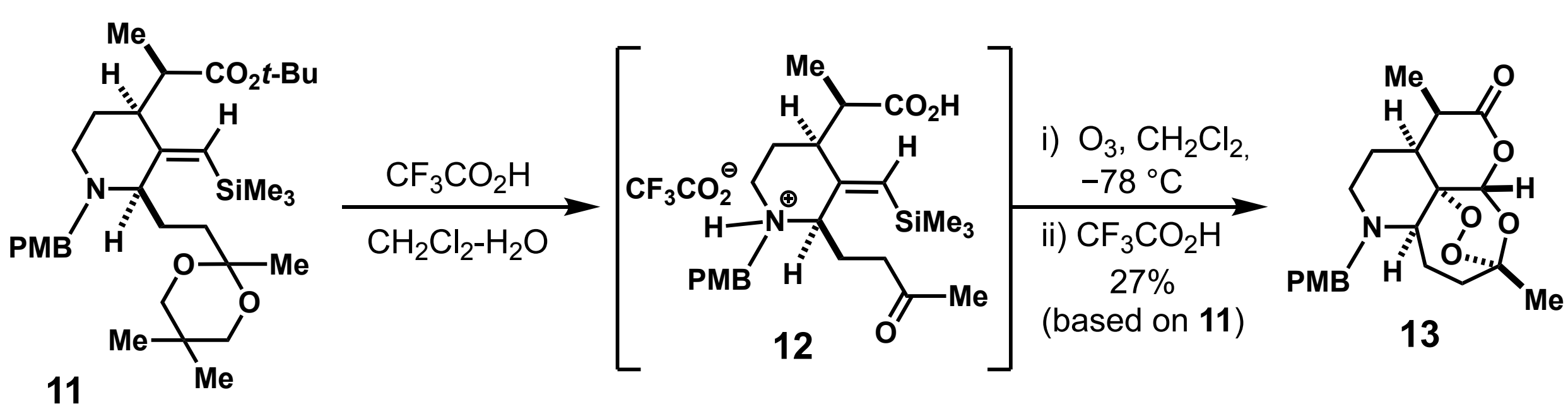

The synthetic intermediate **11** was prepared according to an established protocol developed in our laboratory, and the conversion of **11** to **13** has been previously reported.[3] In the present study, however, workup of the intermediate carboxylic acid **12** as its ammonium salt, as described below, was found to substantially improve reproducibility of the subsequent transformations. To a solution of vinyl silane **11** (903 mg, 1.57 mmol) in wet CH_2Cl_2 (16 mL) was added trifluoroacetic acid (8 mL) at 0 °C. After being allowed to warm to room temperature and stirred for 2.5 h, the mixture was concentrated *in vacuo*. The residue was passed through a short pad of silica gel (eluent: Hexane/AcOEt) to afford the crude carboxylic acid **12** as an ammonium salt, which was used directly in the next step without further purification.

The crude ammonium salt **12** was dissolved in CH_2Cl_2 (210 mL) and transferred into a three-necked round-bottom flask. A stream of O_3 (0.5 L/min) was bubbled into the solution for 10 min at −78 °C. The solution was then purged with nitrogen gas until the blue color disappeared. The resulting mixture was

then treated with trifluoroacetic acid (242 μL, 3.15 mmol) at −78 °C, warmed to room temperature and stirred for 12 h.
The reaction mixture was quenched with saturated aqueous solution of $NaHCO_3$ (90 mL) at 0 °C. After separation of the organic layer, the aqueous layer was extracted with CH_2Cl_2 (150 mL x 2). The combined organic layers were washed with brine, dried over Na_2SO_4. After filtration and concentration *in vacuo*, the residue was purified by silica-gel column chromatography (CH_2Cl_2/Et_2O) to afford **13** [3](165 mg, 424 μmol, 27% from **11**) as a white foamy solid.

2.3 Dimerization to form **16**

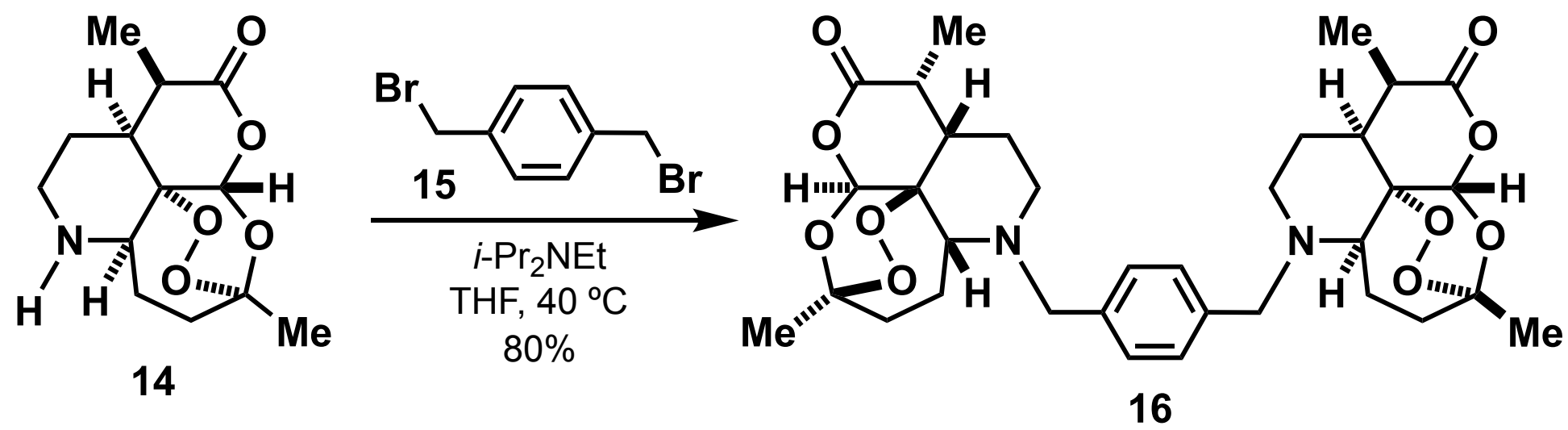

The synthetic intermediate **14** was prepared according to an established protocol developed in our laboratory.[3] A mixture of **14** (19.4 mg, 72.1 μmol), *i*-Pr_2NEt (17.8 μL, 103 μmol), and *p*-xylylene dibromide (**15**, 9.04 mg, 34.2 μmol) in THF (690 μL) was stirred at 35 °C for 18 h. The reaction mixture was treated with additional *i*-Pr_2NEt (17.8 μL, 103 μmol), then warmed to 40 °C, and stirred for 24 h. After being cooled to room temperature, the mixture was diluted with CH_2Cl_2 (10 mL) and treated with a saturated aqueous solution of $NaHCO_3$ (10 mL). After separation of the organic layer, the aqueous layer was extracted with CH_2Cl_2 (10 mL x 2). The combined organic layers were dried over Na_2SO_4. After filtration and concentration *in vacuo*, the residue was purified by silica-gel column chromatography to afford **16** (17.5 mg, 27.3 μmol, 80%) as a white solid.

16: $[\alpha]_D^{22}$ −3.9° (*c* 1.0, $CHCl_3$); ^{1}H-NMR (400 MHz, $CDCl_3$): δ 7.21 (4H, s), 6.30 (2H, s), 4.15 (2H, d, *J* = 13.3 Hz), 3.40–3.33 (2H, m), 3.07 (2H, d, *J* = 13.6 Hz), 2.85 (2H, dt, *J* = 11.6, 3.1 Hz), 2.48 (2H, td, *J* = 14.2, 3.2 Hz), 2.37 (2H, dd, *J* = 10.0, 7.6 Hz), 2.26–2.19 (2H, m), 2.14–2.08 (2H, m), 1.97–1.75 (6H, m), 1.72–1.67 (2H, m), 1.48 (6H, s), 1.25–1.18 (8H, m); ^{13}C-NMR (101 MHz, $CDCl_3$): δ 171.8, 137.7, 128.7, 105.1, 94.0, 77.5, 65.2, 56.6, 51.4, 44.1, 34.6, 32.7, 25.7, 25.2, 23.5, 12.5; HRMS (ESI, *m/z*): $[M+H]^+$ calcd. for $C_{34}H_{45}N_2O_{10}$, 641.3069; found, 641.3068.

2.4 Trifluoromethylation to form monomer **4** [4]

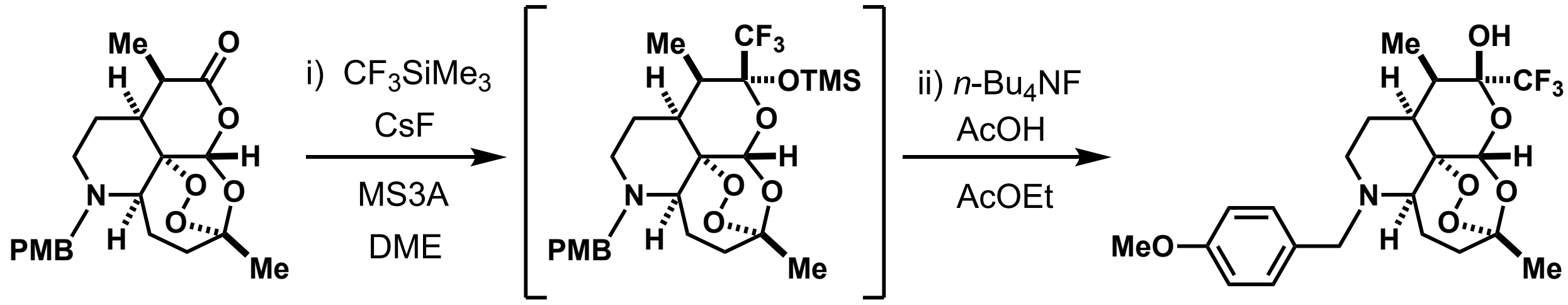

13 **S1** **4** (89% from **13**)

This procedure was adapted from Delpon *et al.*[4] by replacing TBAF (*n*-Bu_4NF) with CsF in the initial trifluoromethylation step. To a solution of **13** (10.3 mg, 26.4 μmol) in 1,2-dimethoxyethane (2.6 mL) was added 3A molecular sieves (430 mg). The mixture was treated with CsF (40.1 mg, 264 μmol) at 0 °C. After stirring for 10 min, CF_3SiMe_3 (39.1 μL, 264 μmol) was added at 0 °C. After stirring for 0.5 h, the resulting mixture was treated with water (5.0 mL) and extracted with AcOEt (20 mL). The organic extract was washed with brine (5.0 mL) and dried over Na_2SO_4. After filtration and concentration *in vacuo*, the crude siloxyketal **S1** was obtained and subjected to the next step without further purification.

To a solution of crude **S1** in AcOEt (2.6 mL) were added AcOH (4.53 μL, 79.2 μmol) and TBAF (158 μL, 79.0 μmol, 0.5 M in THF) at 0 °C. The resulting mixture was stirred at room temperature for 4 h and then treated with saturated aqueous solution of $NaHCO_3$ (5.0 mL) at 0 °C. The mixture was extracted with AcOEt (20 mL). The organic extract was washed with brine (5.0 mL) and dried over Na_2SO_4. After filtration and concentration *in vacuo*, the residue was purified by silica-gel column chromatography to afford **4** (10.8 mg, 23.5 μmol, 89% from **13**) as a white solid.

4: $[\alpha]_D^{21}$ −14.7° (*c* 1.0, $CHCl_3$); ^{1}H-NMR (400 MHz, $CDCl_3$): δ 7.18 (2H, d, *J* = 8.7 Hz), 6.83 (2H, d, *J* = 8.7 Hz), 5.83 (1H, s), 4.08 (1H, d, *J* = 13.3 Hz), 3.79 (3H, s), 3.13–3.02 (1H, br), 2.99 (1H, d, *J* = 13.3 Hz), 2.87–2.70 (2H, m), 2.41 (1H, td, *J* = 14.1, 3.7 Hz), 2.23 (1H, dd, *J* = 10.5, 6.4 Hz), 2.19–2.03 (2H, m), 2.02–1.70 (3H, m), 1.69–1.62 (1H, m), 1.52 (1H, dt, J = 13.4, 4.8 Hz), 1.42 (3H, s), 1.02 (3H, d, *J* = 6.0 Hz); ^{13}C-NMR (101 MHz, $CDCl_3$): δ 158.7, 131.0, 130.0, 122.7 (q, *J* = 287 Hz), 113.7, 104.3, 96.9 (q, *J* = 31 Hz), 89.1, 78.1, 67.3, 56.6, 55.4, 52.2, 45.1, 34.6, 28.2, 25.6, 25.4, 23.2, 12.4; HRMS (ESI, *m/z*): $[M+H]^+$ calcd. for $C_{22}H_{29}F_3NO_6$, 460.1942; found, 460.1945.

2.5 Trifluoromethylation to form dimer **5** [4]

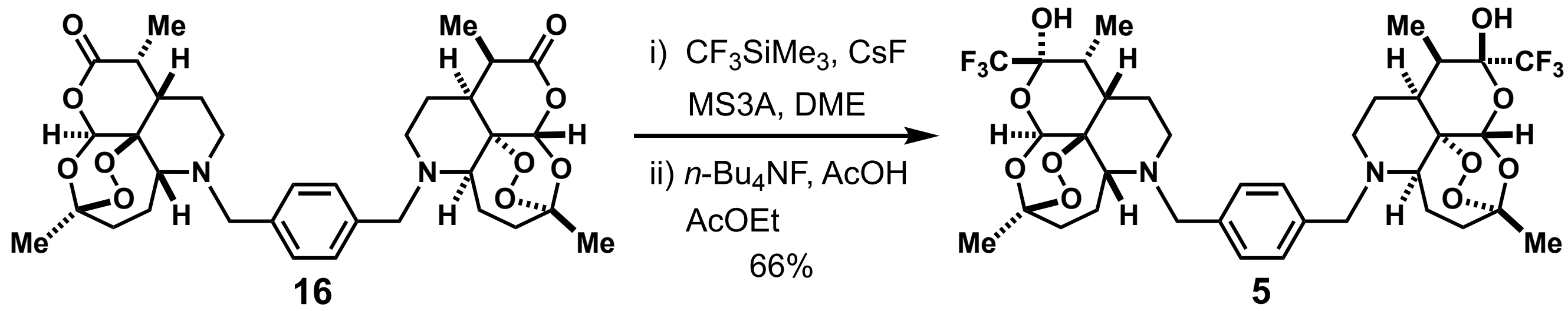

This procedure was adapted from Delpon *et al.*[4] by replacing TBAF (*n*-Bu_4NF) with CsF in the initial trifluoromethylation step. To a solution of **16** (9.14 mg, 14.3 μmol) in 1,2-dimethoxyethane (2.8 mL) was added 3A molecular sieves (400 mg). The mixture was treated with CsF (43.4 mg, 286 μmol) at 0 °C. After stirring for 10 min, CF_3SiMe_3 (42.4 μL, 286 μmol) was added at 0 °C. After stirring for 1 h, the reaction mixture was treated with additional CsF (21.5 mg, 142 μmol) and CF_3SiMe_3 (21.2 μL, 143 μmol), stirred for 30 min at 0 °C, and then quenched with H_2O (5.0 mL). The resulting mixture was extracted with AcOEt (20 mL). The organic extract was washed with brine (5.0 mL) and dried over Na_2SO_4. After filtration and concentration *in vacuo*, the corresponding crude siloxyketal was obtained and subjected to the next step without further purification.

To a solution of the crude siloxyketal in AcOEt (2.8 mL) were added AcOH (4.91 μL, 85.8 μmol) and TBAF (85.8 μL, 42.9 μmol, 0.5 M in THF) at 0 °C. After stirring 1 h at 0 °C, the reaction mixture was treated with TBAF (28.6 μL, 14.3 μmol, 0.5 M THF solution) and stirred at room temperature for 1.5 h. The resulting mixture was treated with saturated aqueous solution of $NaHCO_3$ (5.0 mL) at 0 °C and extracted with AcOEt (20 mL). The organic extract was washed with brine (5.0 mL) and dried over Na_2SO_4. After filtration and concentration *in vacuo*, the residue was purified by silica-gel column chromatography to afford **5** (7.41 mg, 9.49 μmol, 66%) as a white solid.

5: $[\alpha]_D^{21}$ 47.3° (*c* 1.0, $CHCl_3$); ^{1}H-NMR (400 MHz, $CDCl_3$): δ 7.20 (4H, s), 5.85 (2H, s), 4.14 (2H, d, *J* = 13.3 Hz), 3.24 (2H, br), 3.06 (2H, d, *J* = 13.3 Hz), 2.89–2.70 (4H, m), 2.43 (2H, td, *J* = 14.1, 3.5 Hz), 2.27 (2H, dd, *J* = 10.5, 6.9 Hz), 2.21–1.76 (10H, m), 1.71-1.60 (2H, m), 1.54 (2H, dt, *J* = 13.4, 4.8 Hz), 1.43 (6H, s), 1.04 (6H, d, *J* = 6.9 Hz); ^{13}C-NMR (101 MHz, $CDCl_3$): δ 137.8, 128.7, 122.7 (q, *J* = 288 Hz), 104.3, 96.9 (q, *J* = 31 Hz), 89.1, 78.1, 67.3, 56.9, 52.3, 45.1, 34.6, 28.2, 25.6, 25.4, 23.2, 12.4; HRMS (ESI, *m/z*): $[M+H]^+$ calcd. for $C_{36}H_{47}F_6N_2O_{10}$, 781.3130; found, 781.3131.

2.6 Dimerization to form **19**

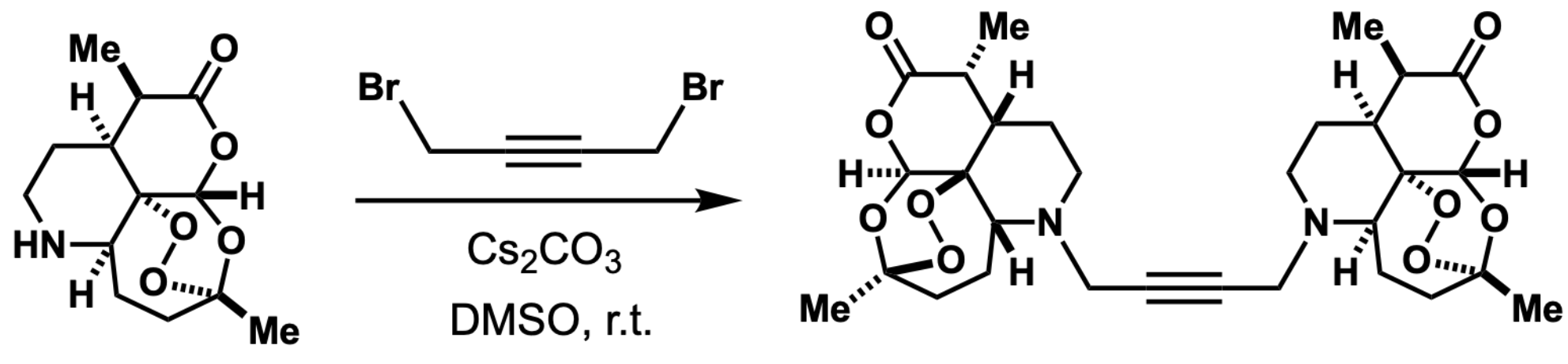

A mixture of **14** (117 mg, 430 μmol), Cs_2CO_3 (191 mg, 586 μmol), and 1,4-dibromo-2-butyne (41.4 mg, 195 μmol) in DMSO (1.0 mL) was stirred at room temperature for 12 h. The reaction mixture was diluted with water (5 mL), and extracted with AcOEt (10 mL x 3). The combined organic layers were washed with brine and dried over Na_2SO_4. After filtration and concentration *in vacuo*, the residue was purified by silica-gel column chromatography to afford **19** (97.7 mg, 166 μmol, 85%) as a white solid.

19: $[\alpha]_D^{24}$ +37.5° (*c* 1.0, DMSO); ^{1}H-NMR (400 MHz, $CDCl_3$): 3.73 (2H, d, *J* = 16.5 Hz), 3.44-3.27 (4H, m), 2.78 (2H, dt, *J* = 11.9, 3.2 Hz), 2.59-2.33 (6H, m), 2.15-1.95 (4H, m), 1.86-1.60 (6H, m), 1.45 (6H, s), 1.39-1.28 (2H, m), 1.22 (6H, d, *J* = 7.3 Hz); ^{13}C-NMR (101 MHz, $CDCl_3$): δ 171.4, 104.8, 93.6, 78.4, 77.1, 62.5, 51.9, 43.6, 42.6, 34.1, 32.5, 25.0, 24.4, 23.3, 12.2; HRMS (ESI, *m/z*): $[M+Na]^+$ calcd. for $C_{30}H_{40}N_2NaO_{10}$, 611.2575; found, 611.2584.

2.7 Trifluoromethylation to form dimer **20** [4]

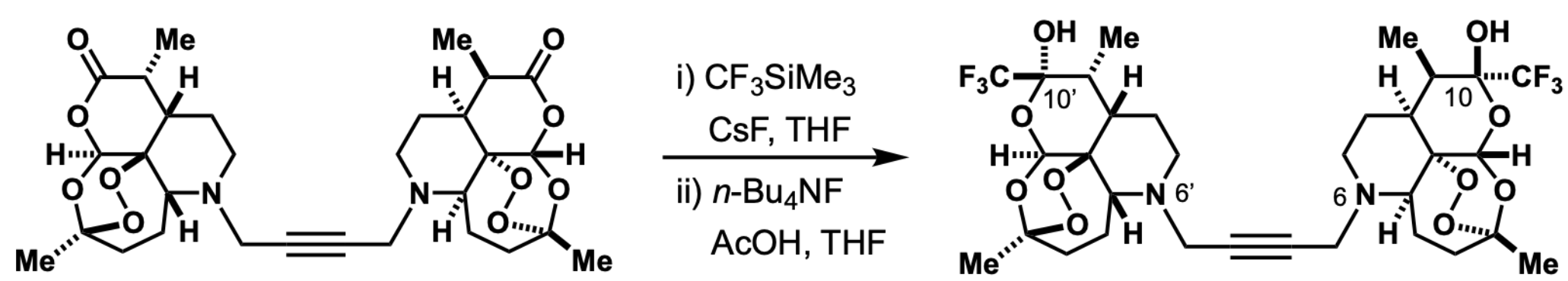

This procedure was adapted from Delpon *et al.*[4] by replacing TBAF (*n*-Bu_4NF) with CsF in the initial trifluoromethylation step. To a solution of **19** (94.8 mg, 161 μmol) in THF (16 mL) was added CsF (49.1 mg, 323 μmol) at room temperature. After stirring for 10 min, CF_3SiMe_3 (240 μL, 1.61 mmol) was added. After stirring for 3 h, the resulting mixture was quenched with H_2O (10 mL) and extracted with AcOEt (20 mL x 3). The combined organic extracts were dried over Na_2SO_4. After filtration and concentration *in vacuo*, the crude siloxyketal was obtained and subjected to the next step without further purification.

To a solution of crude siloxyketal in THF (13 mL) were added AcOH (158 μL, 2.63 mmol) and TBAF (1.32 mL, 1.32 mmol, 1.0 M in THF) at room temperature. The resulting mixture was stirred at room temperature for 1 h and then treated with saturated aqueous solution of $NaHCO_3$ (5.0 mL). The mixture was extracted with AcOEt (20 mL x 3). The combined organic extracts were dried over Na_2SO_4. After filtration and concentration *in vacuo*, the residue was purified by silica-gel column chromatography to afford **20** (42.0 mg, 57.6 μmol, 36%) as a white solid.

20: $[\alpha]_D^{24}$ +26.7° (*c* 1.0, DMSO); ^{1}H-NMR (400 MHz, DMSO-d_6): δ 7.35 (2H, s), 5.56 (2H, s), 3.72 (2H, d, *J* = 16.9 Hz), 3.44-3.25 (4H, m, partially overlapped with residual H_2O), 2.79-2.61 (2H, m), 2.36-2.13 (6H,

m), 2.13-2.00 (4H, m), 1.97-1.84 (2H, m), 1.72-1.50 (4H, m), 1.49-1.39 (2H, m), 1.30 (6H, s), 0.93 (6H, d, *J* = 6.9 Hz); ^{13}C-NMR (101 MHz, $CDCl_3$): δ 122.6 (q, *J* = 288 Hz), 104.1, 96.9 (q, *J* = 31 Hz), 88.8, 78.4, 77.6, 64.5, 53.0, 44.6, 43.1, 34.3, 29.6, 28.2, 25.5, 24.2, 22.9, 12.3; HRMS (ESI, *m/z*): calcd. for $C_{30}H_{43}F_6N_2O_{10}$, $[M+H]^+$ 729.2816; found, 729.2809.

3. Methods for *In Vitro* and ADME Evaluation

3.1 Maintenance of human iPSCs

A human iPSC line (TkDN4-M) was used in this study. TkDN4-M was provided by Dr. Otsu (Institute of Medical Science, The University of Tokyo)[5] and maintained on Vitronectin (Thermo Fisher Scientific)-coated dishes in StemFlex Medium (Thermo Fisher Scientific).

3.2 Generation of *ACTA2*-Luciferase (*ACTA2*-Luc) reporter iPSC line

The establishment and validation of the *ACTA2*-Luc reporter cells shown in Fig. S3 were performed as follows.

An *ACTA2*-luciferase (*ACTA2*-Luc) reporter human iPSC line was generated using a CRISPR-Cas9–mediated knock-in strategy. A targeting donor plasmid was designed to insert, immediately upstream of the endogenous human *ACTA2* stop codon, a P2A peptide followed by the firefly luciferase gene, a bidirectional SV40 polyadenylation signal, and a puromycin resistance cassette driven by the mouse Pgk promoter. The selection cassette was oriented opposite to *ACTA2* transcription and flanked by loxP sequences. Homologous recombination arms of approximately 1.0 kb flanking the *ACTA2* stop codon were incorporated to facilitate targeted integration (Fig. S3A).

TkDN4-M human iPSCs (1.0 × 10^6 cells) were electroporated with 1 µg of an spCas9-expressing plasmid, 1 µg each of two ACTA2-targeting gRNA-expressing plasmids, and 1 µg of the *ACTA2*-Luc donor plasmid using a Nucleofector 2b (Lonza) with the B-016 program and the Human Stem Cell Nucleofector Starter Kit. The gRNA target sequences were 5′-TAGGTAACGAGTCAGAGCTTTGG-3′ and 5′-ATAAATCTGAAATAGGCTACTGG-3′.

Two days after electroporation, puromycin was added to the culture medium at a final concentration of 0.6 µg/mL, and cells were selected for 7 days. Emerging colonies were manually picked and expanded. Correct heterozygous integration of the donor cassette into the *ACTA2* locus was confirmed by PCR using primer sets spanning the 5′ and 3′ junctions (Fig. S3B), followed by Sanger sequencing. The non-targeted allele was further verified to be free from Cas9-induced mutations.

To validate reporter functionality, quiescent hepatic stellate cells (qHSCs) were induced from the *ACTA2*-Luc reporter iPSCs and cultured under two-dimensional conditions. Upon activation, *ACTA2*-Luc iPSC-derived hepatic stellate cells exhibited a robust and time-dependent increase in luciferase activity, consistent with induction of *ACTA2* expression (Fig. S3C). This *ACTA2*-Luc reporter iPSC line enables real-time and highly sensitive monitoring of *ACTA2* promoter activity via luminescence readout.

3.3 Generation of qHSC-like cells from human iPSCs (iPSC-derived qHSCs)

Human qHSC-like cells were induced from iPSCs in a three-dimensional EB culture system. To form EBs, TkDN4-M iPSCs or *ACTA2*-Luc reporter iPSCs were dissociated into small cell clusters and seeded on Ultra-Low Attachment plates (Corning) at a density of 10,000 cells/cm^2. Cells were cultured in Stempro-34 SFM medium (Thermo Fisher Scientific) supplemented with Y27632 (10 µM) (Wako Pure Chemical Industries, Ltd.) and bone morphogenetic protein 4 (BMP4) (2 ng/mL) (PeproTech) (day 0 to day 1), Activin A (TkDN4-M: 5 ng/mL, FF-1: 1.25 ng/mL, and FF-2: 5 ng/mL) (PeproTech), basic fibroblast growth factor (bFGF) (5 ng/mL) (Thermo Fisher Scientific), and BMP4 (TkDN4-M: 30 ng/mL, FF-1: 120 ng/mL, and FF-2: 180 ng/mL) (day 1 to day 4), vascular endothelial growth factor (VEGF) (10 ng/mL) (PeproTech), SB431542 (5.4 µM) (Tocris), Dorsomorphin dihydrochloride (0.5 µM) (Tocris), and Y27632 (10 µM) (day 4 to day 9). EBs were maintained in a 5% CO_2, 4% O_2 environment from day 0 to day 6 and in a 5% CO_2, ambient O_2 environment from day 6 to day 9. After 9 days of culture, EBs were collected and dissociated in 1xTrypLE select (Thermo Fisher Scientific)/1 mM EDTA solution for 10 min at 37 °C. Cells were blocked by FcR blocking reagent (Miltenyi Biotech) for 20 min and incubated with APC-conjugated anti-human CD271 antibody (Miltenyi) for 30 min on ice, followed by incubation with anti-APC

MACS beads (Miltenyi) in MACS buffer (Miltenyi) for 15 min at 4 °C. CD271+ qHSC-like cells were isolated using autoMACS® Pro Separator (Miltenyi).

3.4 Luciferase reporter assay

qHSC-like cells derived from *ACTA2*-Luc reporter iPSCs were seeded on Cellmatrix Type I-C (Nitta gelatin)-coated 96-well plates at a density of 5,000 cells/well in Stempro-34 SFM medium supplemented with Y27632 (10 µM). The medium was replaced every 24 hours. After 48 hours of culture at 37 °C in a humidified atmosphere of 5% CO_2 and ambient O_2, test compounds prepared as stock solutions in DMSO were diluted in StemPro-34 SFM medium and added to each well to give a final compound concentration of 2 µM, with a final DMSO concentration of 0.1% (v/v). Cells were incubated with each compound for 72 hours under the same conditions, and firefly luciferase activity was measured using the Dual-Glo Luciferase Assay System (Promega).

3.5 Compound treatment in iPSC-derived qHSCs

iPSC-derived qHSCs were seeded on Cellmatrix Type I-C-coated 12-well plates at a density of 20,000 cells/cm² in StemPro-34 SFM medium supplemented with Y-27632 (10 µM) and 1% penicillin-streptomycin-L-glutamine (PSG). After 24 hours, the medium was replaced with StemPro-34 SFM containing 1% PSG and subsequently changed every 24 hours. After 48 hours of culture at 37 °C in a humidified atmosphere of 5% CO_2 and ambient O_2, test compounds prepared as stock solutions in DMSO were diluted in StemPro-34 SFM medium and added to each well to give a final compound concentration of 2 µM, with a final DMSO concentration of 0.1% (v/v). Cells were treated for 72 hours, a duration selected based on preliminary optimization experiments that provided robust and reproducible modulation of fibrosis-associated gene expression. Cells were then harvested for RT-qPCR analysis.

3.6 Cell viability and cytotoxicity assay

iPSC-derived qHSCs were seeded on Cellmatrix Type I-C-coated 96-well plates at a density of 5,000 cells/cm² in StemPro-34 SFM medium supplemented with Y-27632 (10 µM) and 1% penicillin-streptomycin-L-glutamine (PSG). After 24 hours, the medium was replaced with StemPro-34 SFM containing 1% PSG and subsequently changed every 24 hours. After 48 hours of culture at 37 °C in a humidified atmosphere of 5% CO_2 and ambient O_2, test compounds prepared as stock solutions in DMSO were diluted in StemPro-34 SFM medium and added to each well to give a final compound concentration of 500 nM, with a final DMSO concentration of 0.1% (v/v). Cells were treated for 72 hours, after which the medium was replaced with StemPro-34 SFM medium containing 0.2% Calcein AM and Ethidium Homodimer (CELL BIOLABS). Cells were incubated for 30 minutes, washed twice with medium, and fluorescence images were acquired using a fluorescence microscope (BZ-X810, Keyence).

3.7 Compound treatment and TGF-β stimulation in iPSC-derived qHSCs

hiPSC-derived qHSC-like cells were seeded on Cellmatrix Type I-C-coated 12-well plates at a density of 20,000 cells/cm² in StemPro-34 SFM medium supplemented with Y-27632 (10 µM) and 1% PSG. After 24 hours, the medium was replaced with StemPro-34 SFM containing 1% PSG and subsequently changed every 24 hours. After 48 hours of culture at 37 °C in a humidified atmosphere of 5% CO_2 and ambient O_2, test compounds prepared as stock solutions in DMSO were diluted in StemPro-34 SFM medium and added to each well to give a final concentration of 500 nM, with a final DMSO concentration of 0.1% (v/v). After 24 hours of compound treatment, cells were stimulated with recombinant human TGF-β1 (Wako) at a final concentration of 10 ng/mL and cultured for an additional 24 hours. Cells were then harvested for RT-qPCR analysis.

3.8 Quantitative Reverse Transcription (qRT)-PCR

Total RNA from cultured cells was extracted using NucleoSpin® RNA Plus (MACHEREY-NAGEL) according to the manufacturer's protocol. 1 μg of RNA was used to synthesize cDNA using PrimeScript RT Master Mix Kit (Takara bio). Quantitative real-time PCR was performed using TB Green® *Premix Ex Taq*™ II (Takara Bio) on a LightCycler® System (Roche) in technical duplicates. The sequences of primers are summarized in Table S4.

3.9 Immunocytochemistry

hiPSC-derived qHSC-like cells were seeded on Cellmatrix Type I-C-coated 6-well plates at a density of 20,000 cells/cm² in StemPro-34 SFM medium supplemented with Y-27632 (10 μM) and 1% penicillin-streptomycin-L-glutamine (PSG). After 24 hours, the medium was replaced with StemPro-34 SFM containing 1% PSG. After 24 hours of culture at 37 °C in a humidified atmosphere of 5% CO_2 and ambient O_2, DMSO or compound **5** prepared as stock solutions in DMSO were diluted in StemPro-34 SFM medium and added to each well to give a final compound concentration of 300 nM, with a final DMSO concentration of 0.1% (v/v). After 48 hours of incubation under the same conditions, cells were fixed with 10% formaldehyde for 20 min at 4 °C, washed three times with PBS (5 min each), and blocked with blocking solution (3% FBS, 0.2% Triton X-100 in PBS) for 20 min at 4 °C. Cells were incubated overnight at 4 °C with primary antibodies against Collagen I (Bio-Rad) and α-SMA (Sigma). After washing, cells were incubated for 20 min at room temperature with fluorescent secondary antibodies Alexa Fluor 488–conjugated anti-rabbit IgG (Thermo Fisher) and Alexa Fluor 555–conjugated anti-mouse IgG (Thermo Fisher), together with Hoechst 33342 (Thermo Fisher) for nuclear staining. Images were acquired using a fluorescence microscope (BZ-X810, Keyence).

3.10 Primary dermal fibroblasts from systemic sclerosis (SSc) patients

Primary dermal fibroblasts were isolated from patients' skin biopsy samples obtained for diagnostic purposes. They were obtained from the forearms using a 4 mm dermal punch. Half of the skin piece was placed on a 6 cm dish and cultured in minimum essential medium (Sigma) supplemented with 10% fetal bovine serum. When the plate became confluent with the isolated fibroblasts (P0), the cells were passaged into a T-75 flask and cultured until confluent. These P1 cells were frozen and kept in liquid nitrogen until use. Patients having been treated with corticosteroids or other immunosuppressants prior to the biopsy were excluded. The use of human-derived samples was approved by the Ethical Committee of the Graduate School of Medicine, The University of Tokyo (Approval number: 0695) and was conducted in accordance with the Declaration of Helsinki. Written informed consent was obtained from all participants.

3.11 Compound treatment in dermal fibroblasts from SSc patients

Primary dermal fibroblasts from systemic sclerosis patients were seeded on 12-well plates at a density of 5,000 cells/cm² in MEM (Wako) supplemented with 10% FBS (Gibco) and 1% antibiotics. The medium was replaced every 24 hours. After 72 hours of culture at 37 °C in a humidified atmosphere of 5% CO_2 and ambient O_2, test compounds prepared as stock solutions in DMSO were diluted in MEM medium and added to each well to give a final compound concentration of 0.1,1, or 10 μM, with a final DMSO concentration of 0.1% (v/v). After 24 hours of compound treatment, cells were then harvested for RT-qPCR analysis. The procedure for RT-qPCR analysis is described in Section 3.8, and the primer sequences are listed in Table S4.

3.12 Microsomal metabolic stability assay

Microsomal metabolic stability assays were performed by Axcelead Drug Discovery Partners, Inc. using human and mouse liver microsomes. Test compounds were incubated at a final concentration of 1 μM with liver microsomes at a final protein concentration of 0.2 mg/mL in the presence of an NADPH-generating system. The final organic solvent concentration was 0.01% DMSO and 0.99% acetonitrile. Reactions were performed at 37 °C and initiated by the addition of the NADPH-generating system. Aliquots were collected at 0, 15, and 30 min, and the reactions were terminated by the addition of acetonitrile. The remaining parent compound was quantified by LC/MS/MS without a calibration curve. Each test compound was analyzed in duplicate at each time point. Blank samples without test compound and positive control compounds, including cilostazol, flutamide, and quinidine, were included in the assay. The percentage of compound remaining was plotted as a function of incubation time, and the elimination rate constant *k* was calculated by fitting the remaining ratio to a first-order exponential decay model. Intrinsic clearance was calculated according to the following equation:

$$CL_{\text{int}} = 1000 \times k / m$$

where CL_{int} is the intrinsic clearance in μL/min/mg protein, *k* is the first-order elimination rate constant, and *m* is the microsomal protein concentration in mg/mL. The microsomal half-life was calculated as $t_{1/2} = 0.693 / k$.

3.13 Compound treatment for RAW264.7 murine macrophage cells

RAW264.7 murine macrophage cells, kindly provided by SBI Biotech Co., Ltd., were seeded in 24-well plates at a density of 10,000 cells/well in DMEM (Wako) supplemented with 10% FBS (Gibco) and 1% penicillin–streptomycin–L-glutamine. After 24 hours of culture at 37 °C in a humidified atmosphere of 5% CO_2 and ambient O_2, test compounds prepared as stock solutions in DMSO were diluted in DMEM and added to each well to give a final compound concentration of 10 μM, with a final DMSO concentration of 0.1% (v/v). After 1 hour of pretreatment with the test compounds, lipopolysaccharide (LPS; Merck) was added to each well at a final concentration of 100 ng/mL, and the cells were cultured for an additional 6 hours. The procedure for RT-qPCR analysis is described in Section 3.8, and the primer sequences are listed in Table S4.

4. Transcriptomic analysis in iPSC-derived quiescent hepatic stellate cells

4.1 Compound treatment for RNA extraction

Compound treatment was performed as described in section 3.5, except that the final compound concentration was 500 nM and the treatment duration was 48 h. Total RNA was extracted from cultured cells using the NucleoSpin® RNA Plus kit according to the manufacturer's protocol. The purified RNA samples were stored at –20 °C until shipment to Azenta Life Sciences (Suzhou, China) for sequencing.

4.2 mRNA library preparation and sequencing

At Azenta, RNA concentration and purity were determined spectrophotometrically using a NanoDrop instrument, and RNA integrity was assessed prior to library construction. Samples meeting the quality criteria (RIN > 9.5) were subjected to sequencing. For each sample, 1 µg of total RNA was used for library construction. Poly(A)+ mRNA was isolated using oligo(dT) magnetic beads, followed by fragmentation in the presence of divalent cations under high temperature. First-strand cDNA was synthesized using random primers, and second-strand cDNA was generated subsequently. The double-stranded cDNA was end-repaired, subjected to dA-tailing, and ligated with sequencing adapters via T-A ligation. Adapter-ligated fragments were purified and size-selected using DNA Clean Beads. Libraries were amplified by PCR using P5 and P7 primers and validated for quality and insert size distribution. Indexed libraries were pooled and sequenced on an Illumina HiSeq/NovaSeq platform using a 2×150 bp paired-end configuration according to the manufacturer's instructions. On average, 38–51 million high-quality reads per sample were obtained, with Q30 > 91% and GC contents around 48%, ensuring data suitability for downstream analysis.

4.3 Differential gene expression and pathway enrichment analyses

Raw reads were quality-checked using FastQC (v0.10.1) and trimmed using Cutadapt (v1.9.1). Clean reads were aligned to the human reference genome using HISAT2[6] (v2.2.1) by Azenta Life Sciences. Gene-level counts were generated with HTSeq (v0.6.1), resulting in a raw count matrix for downstream analysis. The reference datasets were obtained from the Ensembl database (https://www.ensembl.org/) as follows:

- Genome: Homo_sapiens.GRCh38.dna.primary_assembly.fa.gz
- Gene annotation: Homo_sapiens.GRCh38.107.chr.gff3.gz
- Transcript sequences: Homo_sapiens.GRCh38.cdna.all.fa.gz

Principal component analysis (PCA) was performed using iDEP[7] (v2.21). Raw count data were imported into iDEP, where lowly expressed genes were removed using default filtering criteria, followed by normalization to counts per million (CPM) and $\log_2$ transformation prior to PCA.

Differential expression analysis was performed using DESeq2[8] within iDEP based on the raw count matrix. Genes with |log2(fold change)| > 1 and Benjamini–Hochberg–adjusted p values (FDR) < 0.05 were considered significantly differentially expressed.

KEGG[9,10] pathway analysis was conducted using the Gene Set Enrichment Analysis (GSEA)[11] algorithm implemented in iDEP. Genes were pre-ranked by $\log_2$(fold change), and pathway significance in the dimer **5** vs monomer **4** comparison was assessed based on normalized enrichment scores (NES) with FDR-adjusted p values < 0.05. From the significant pathways, those with clear relevance to fibrosis were selected for visualization. Pathway diagrams were generated using the Pathview package[12] based on KEGG pathway maps.

RNA-seq data generated in this study have been deposited in NCBI under BioProject accession number PRJNA1370295. Raw sequence data are available under SRA accession numbers SRR36217662–SRR36217670. Sample metadata are registered under BioSample accessions

SAMN53424923–SAMN53424931. RNA-seq sample information and accession numbers are summarized in Table S5.

Table S5. RNA-seq sample information and accession numbers.

Sample ID	Treatment	Replicate	BioSample ID	SRA Run ID	Read layout
qHSC-DMSO-rep1	DMSO	1	SAMN53424923	SRR36217670	PE150
qHSC-DMSO-rep2	DMSO	2	SAMN53424924	SRR36217669	PE150
qHSC-DMSO-rep3	DMSO	3	SAMN53424925	SRR36217668	PE150
qHSC-Compound4-rep1	Compound **4**	1	SAMN53424926	SRR36217667	PE150
qHSC-Compound4-rep2	Compound **4**	2	SAMN53424927	SRR36217666	PE150
qHSC-Compound4-rep3	Compound **4**	3	SAMN53424928	SRR36217665	PE150
qHSC-Compound5-rep1	Compound **5**	1	SAMN53424929	SRR36217664	PE150
qHSC-Compound5-rep2	Compound **5**	2	SAMN53424930	SRR36217663	PE150
qHSC-Compound5-rep3	Compound **5**	3	SAMN53424931	SRR36217662	PE150

4.4 Pathview-based KEGG pathway mapping

To facilitate mechanistic interpretation of the RNA-seq results, Fig. S8 summarizes key inflammation-driven and TGF-β–dependent signaling pathways implicated in fibroblast activation and extracellular matrix production. This schematic illustration is informed by differential expression and pathway enrichment analyses performed in this study and is intended to provide a unifying framework for the Pathview-based KEGG pathway mappings described below (Figs. S9–S14).

Engagement of the TLR4–MD2 complex activates NF-κB signaling, leading to transcriptional induction of pro-inflammatory cytokines, including *IL6*, *IL12*, and *TNF* (TNFα), which are known to contribute to inflammation-driven fibroblast activation. In parallel, TGF-β signaling through TGFβR1 and TGFβR2 activates canonical SMAD2/3–SMAD4 signaling as well as non-canonical pathways involving RhoA–ROCK and PI3K–Akt–mTOR, thereby promoting the transcription of profibrotic genes such as *ACTA2* and *COL1A1*. While 6-aza-artemisinins (**4** or **5**) may affect additional pathways, Fig. S8 focuses on representative pathways most directly relevant to fibroblast activation and fibrotic gene expression in the present study.

5. Methods for *In Vivo* Activity Evaluation

5.1 Ethics statement

This study was approved by the Institutional Animal Care and Use Committee of the Tohoku University Environmental & Safety Committee (Approval number: 2022-068) and were conducted in accordance with the Regulations for Animal Experiments and Related Activities at Tohoku University.

5.2 Mice

We used C57BL/6NCR, 8-week-old female wild-type mice. All mice were housed in four per cage in specific-pathogen-free conditions and maintained on a 12-hour light/dark cycle in a constant temperature (20 °C to 22 °C) and humidity (45 to 55%) with ad libitum access to food and water.

5.3 A BLM-induced murine model of skin fibrosis

The model of skin fibrosis *in vivo* was created with bleomycin (BLM). Bleomycin (Nippon Kayaku, Tokyo, Japan) was dissolved in phosphate-buffered saline (PBS) (1 mg/ml) and sterilized by filtration. BLM (200 µg) or PBS was injected subcutaneously into a single location on the shaved lower backs of mice. The injection was administered for four weeks every other day. The artemisinin derivatives, **4** (5 mg/kg), **5** (0.3 mg/kg), and artesunate (**2**) (4.2 mg/kg) were administered intraperitoneally alongside bleomycin. We utilized the prevention and treatment models to evaluate the effects of the artemisinin derivatives on skin fibrosis. The detailed protocols of these models are shown in Fig. 4A and Supplementary Fig. S17A. Artemisinin derivatives were initiated one day before the start of bleomycin treatments and administered during the whole treatment course in the prevention model, while they were given only during the second half of the bleomycin treatments in the treatment model. **4** was given with 5-aminolevulinic acid (ALA) (Nacalai Tesque, Kyoto, Japan) in drinking water at the concentration of (1.5 mg/ml). The drinking water was changed every five days. ALA was not given with **5** and artesunate.

The reagent was reconstituted as follows. **4** (25 mg/ml in dimethyl sulfoxide, DMSO, Sigma-Aldrich, St. Louis, USA) was dissolved in a pH 4.6 citrate buffer to achieve a 5 mg/kg concentration. 100 µl of this solution was then administered intraperitoneally to each mouse. **5** (375 µg/ml in DMSO) was adjusted to 0.3 mg/kg in corn oil (Sigma-Aldrich), and 200 µl of this solution was administered intraperitoneally to each mouse. Artesunate (21 mg/ml at DMSO, Tokyo Chemical Industry, Tokyo, Japan) was dissolved in PBS at 4.2 mg/kg, and 100 µl of this solution was given intraperitoneally. All injections were conducted under inhalation anesthetics of isoflurane. After the 4-week experiment, mice were euthanized by cervical dislocation. The back skin specimens were excised for hematoxylin-eosin staining, Masson's trichrome staining, immunohistochemistry staining, and RNA extraction.

5.4 Dermal thickness measurement

Dermal thickness was measured to assess the improvement of the skin fibrosis. Dermal thickness was measured by ImageJ software (US National Institute of Health) and was defined based on the average of 10 randomly chosen measurements of the dermis in each specimen.

5.5 RNA isolation and quantitative reverse transcription (qRT)-PCR

Total RNA from murine skin was extracted using RNeasy mini kit (Qiagen, Hilden, Germany). 2 µg of RNA was used to synthesize cDNA using ReverTra Ace qPCR RT master mix (TOYOBO, Osaka, Japan). Quantitative real-time PCR was performed using THUNDERBIRD SYBR qPCR Mix (TOYOBO) on a StepOnePlus Real-Time PCR System (Thermo Fisher Scientific) in technical triplicates. The sequences of primers are summarized in Table S4.

5.6 Immunohistochemistry

Chromogenic immunohistochemistry was performed on formalin-fixed, paraffin-embedded skin sections. DAB (3, 3'-diaminobenzidine) was used as a chromogenic substrate. A primary antibody against α-smooth muscle actin (α-SMA) (Abacam, Cambridge, UK) was used. Antigen retrieval was performed using Dako Target Retrieval Solution pH 6 (Dako, Agilent, Santa Clara, CA, USA). The primary antibody was incubated overnight at 4 °C. Vectastain Elite ABC kit (Vector Laboratories, Burlingame, CA, USA) was used thereafter, which includes biotinylated secondary antibodies and reagents for avidin-biotin complex formation. Counterstaining was performed using hematoxylin. α-SMA-positive fibroblasts were counted in three randomly selected sites at a magnification of 200x. Fibroblasts are defined as cells found in the dermal stroma with spindle-shaped nuclei. Two independent dermatologists (T. Ikawa and Y. Asano) determined the α-SMA positivity of fibroblasts.

6. Statistical analysis

Statistical analyses were performed according to the experimental design of each assay. For the *in vitro* experiments shown in Figures 2, 3, S5, S15, S16, and S18, statistical comparisons were performed using two-tailed Welch's t-tests in Microsoft Excel. This test was selected because these assays were conducted as exploratory cell-based evaluations using independent culture wells, and equal variance between groups was not assumed. Data are presented as the mean ± standard error of the mean. The number of biological replicates is indicated in each figure legend. For Figures 3 and S16, n = 3 independent culture wells from the same patient-derived fibroblast line.

For the *in vivo* experiments shown in Figures 4, 5, and S17, statistical analysis was conducted using GraphPad Prism 9 software (GraphPad Software, San Diego, CA, USA). For comparisons between two groups, the Mann–Whitney U test was used. For comparisons among multiple groups, the Kruskal–Wallis test followed by Dunn's multiple-comparison test was used. The number of biological replicates was n = 4 unless otherwise indicated. The error bars on each graph show the standard error of the mean. Statistical significance was defined as $p<0.05$. In all figures, statistical significance is denoted as follows: * $p<0.05$, ** $p<0.01$, and *** $p<0.001$.

7. References

(1) Tojo, M.; Hamashima, Y.; Hanyu, A.; Kajimoto, T.; Saitoh, M.; Miyazono, K.; Node, M.; Imamura, T. The ALK-5 Inhibitor A-83-01 Inhibits Smad Signaling and Epithelial-to-Mesenchymal Transition by Transforming Growth Factor-β. *Cancer Sci.* **2005**, *96*, 791–800.

(2) Wang, Y.; Shan, X.; Chen, G.; Jiang, L.; Wang, Z.; Fang, Q.; Liu, X.; Wang, J.; Zhang, Y.; Wu, W.; Liang, G. MD-2 as the Target of a Novel Small Molecule, L6H21, in the Attenuation of LPS-Induced Inflammatory Response and Sepsis. *Br. J. Pharmacol.* **2015**, *172*, 4391–4405.

(3) Bonepally, K. R.; Hiruma, T.; Mizoguchi, H.; Ochiai, K.; Suzuki, S.; Oikawa, H.; Ishiyama, A.; Hokari, R.; Iwatsuki, M.; Otoguro, K.; Omura, S.; Oguri, H. Design and *De Novo* Synthesis of 6-Aza-artemisinins. *Org. Lett.* **2018**, *20*, 4667–4671.

(4) Begue, J. P.; Bonnet-Delpon, D. Fluoroartemisinins: Metabolically More Stable Antimalarial Artemisinin Derivatives. *ChemMedChem* **2007**, *2*, 608–624.

(5) Takayama, N.; Nishimura, S.; Nakamura, S.; Shimizu, T.; Ohnishi, R.; Endo, H.; Yamaguchi, T.; Otsu, M.; Nishimura, K.; Nakanishi, M.; Sawaguchi, A.; Nagai, R.; Takahashi, K.; Yamanaka, S.; Nakauchi, H.; Eto, K. Transient Activation of c-MYC Expression Is Critical for Efficient Platelet Generation from Human Induced Pluripotent Stem Cells. *J. Exp. Med.* **2010**, *207*, 2817-2830.

(6) Kim, D.; Paggi, J. M.; Park, C.; Bennett, C.; Salzberg, S. L. Graph-Based Genome Alignment and Genotyping with HISAT2 and HISAT-Genotype. *Nat. Biotechnol.* **2019**, *37*, 907–915.

(7) Ge, S. X.; Son, E. W.; Yao, R. iDEP: An Integrated Web Application for Differential Expression and Pathway Analysis of RNA-Seq Data. *BMC Bioinformatics* **2018**, *19*, 534.

(8) Love, M. I.; Huber, W.; Anders, S. Moderated Estimation of Fold Change and Dispersion for RNA-Seq Data with DESeq2. *Genome Biol.* **2014**, *15*, 550.

(9) Kanehisa, M.; Goto, S. KEGG: Kyoto Encyclopedia of Genes and Genomes. *Nucleic Acids Res.* **2000**, *28*, 27–30.

(10) Kanehisa, M.; Furumichi, M.; Sato, Y.; Kawashima, M.; Ishiguro-Watanabe, M. KEGG for Taxonomy-Based Analysis of Pathways and Genomes. *Nucleic Acids Res.* **2023**, *51*, D587–D592.

(11) Subramanian, A.; Tamayo, P.; Mootha, V. K.; Mukherjee, S.; Ebert, B. L.; Gillette, M. A.; Paulovich, A.; Pomeroy, S. L.; Golub, T. R.; Lander, E. S. et al. Gene Set Enrichment Analysis: A Knowledge-Based Approach for Interpreting Genome-Wide Expression Profiles. *Proc. Natl. Acad. Sci. U. S. A.* **2005**, *102*, 15545–15550.

(12) Luo, W.; Brouwer, C. Pathview: an R/Bioconductor Package for Pathway-Based Data Integration and Visualization. *Bioinformatics* **2013**, *29*, 1830–1831.

8. NMR Spectra

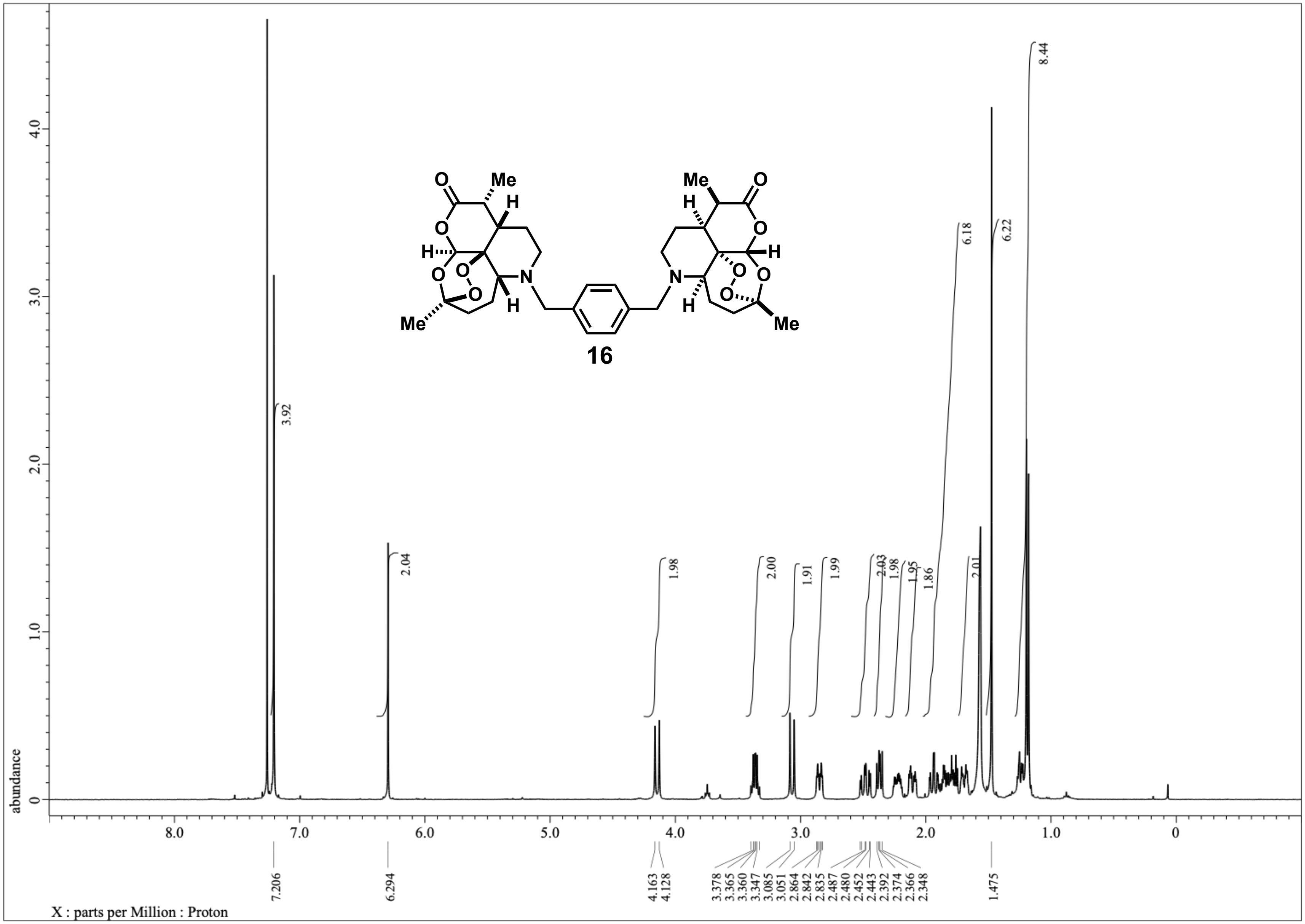

Figure S19. ^{1}H NMR (400 MHz, $CDCl_3$) spectrum of compound **16**

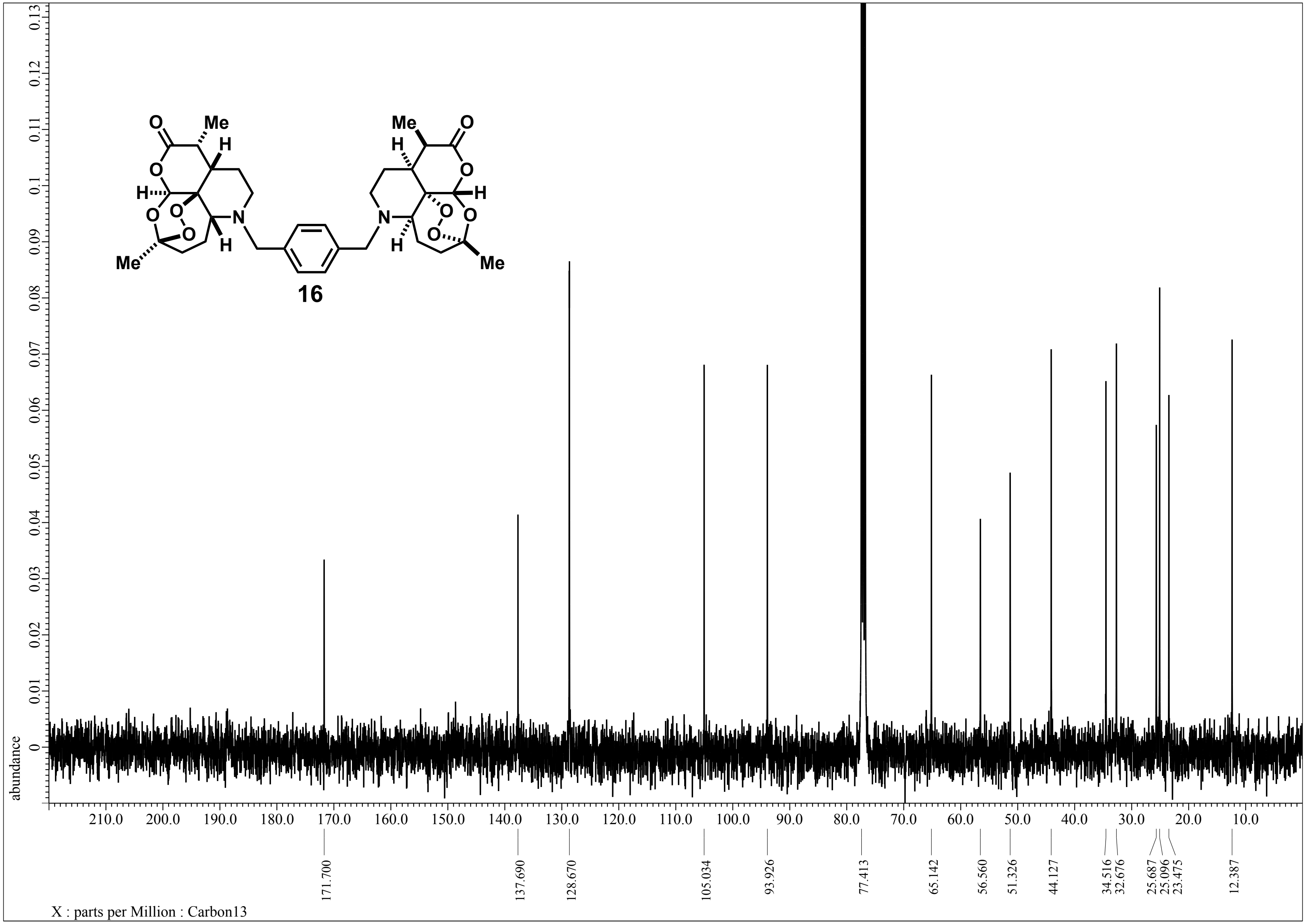

Figure S20. ^{13}C NMR (101 MHz, $CDCl_3$) spectrum of compound **16**

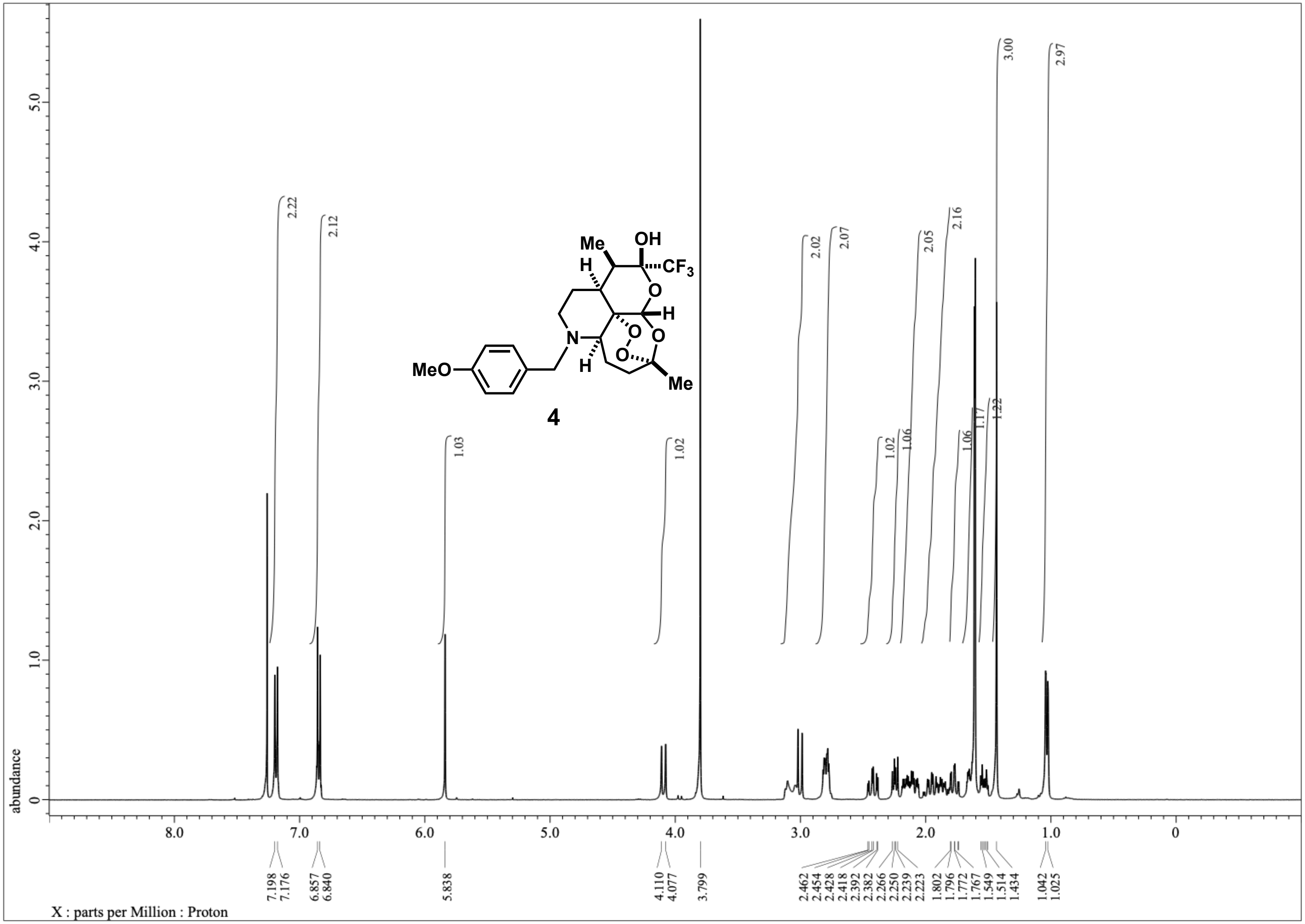

Figure S21. ^{1}H NMR (400 MHz, $CDCl_3$) spectrum of compound **4**

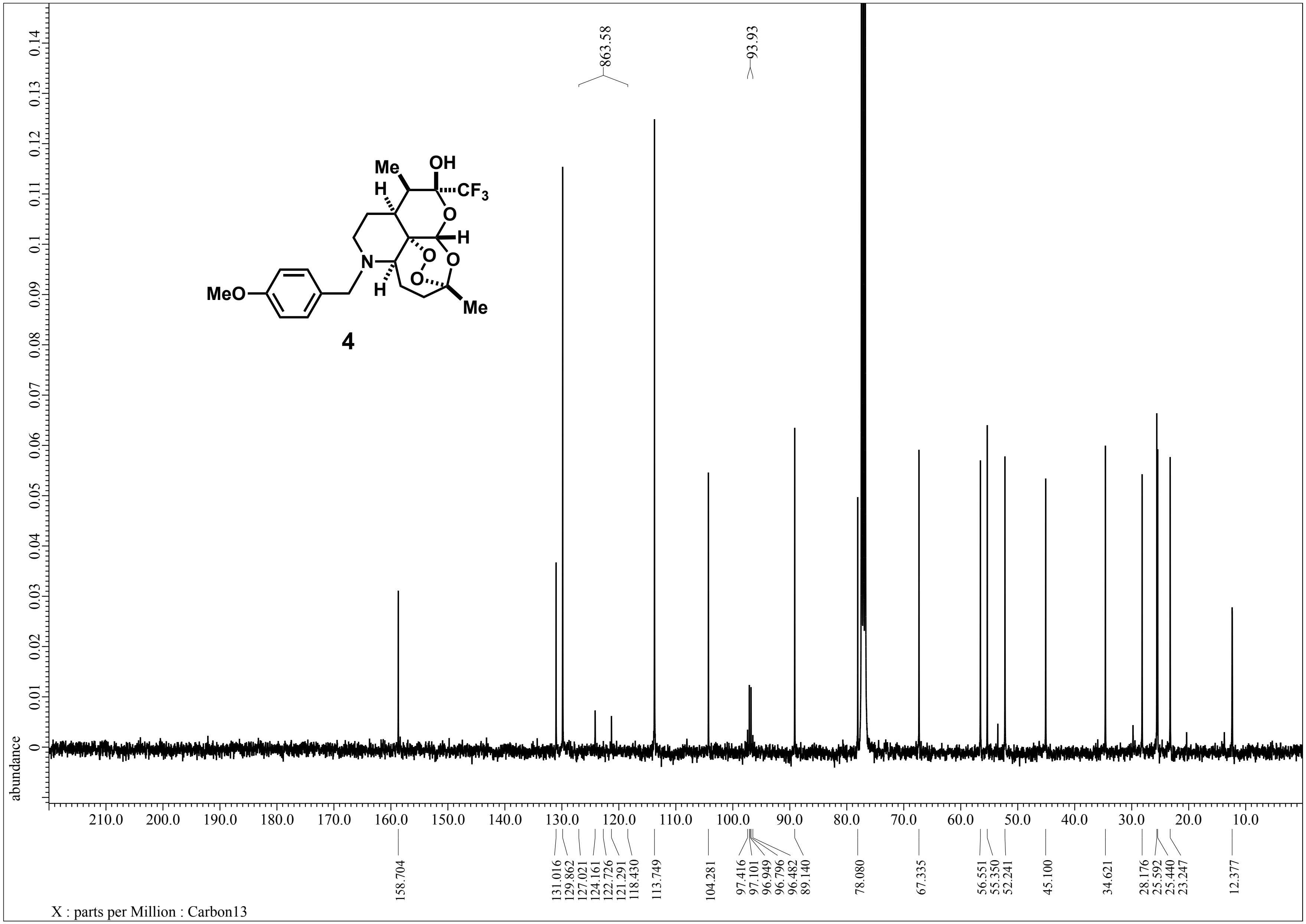

Figure S22. ^{13}C NMR (101 MHz, $CDCl_3$) spectrum of compound **4**

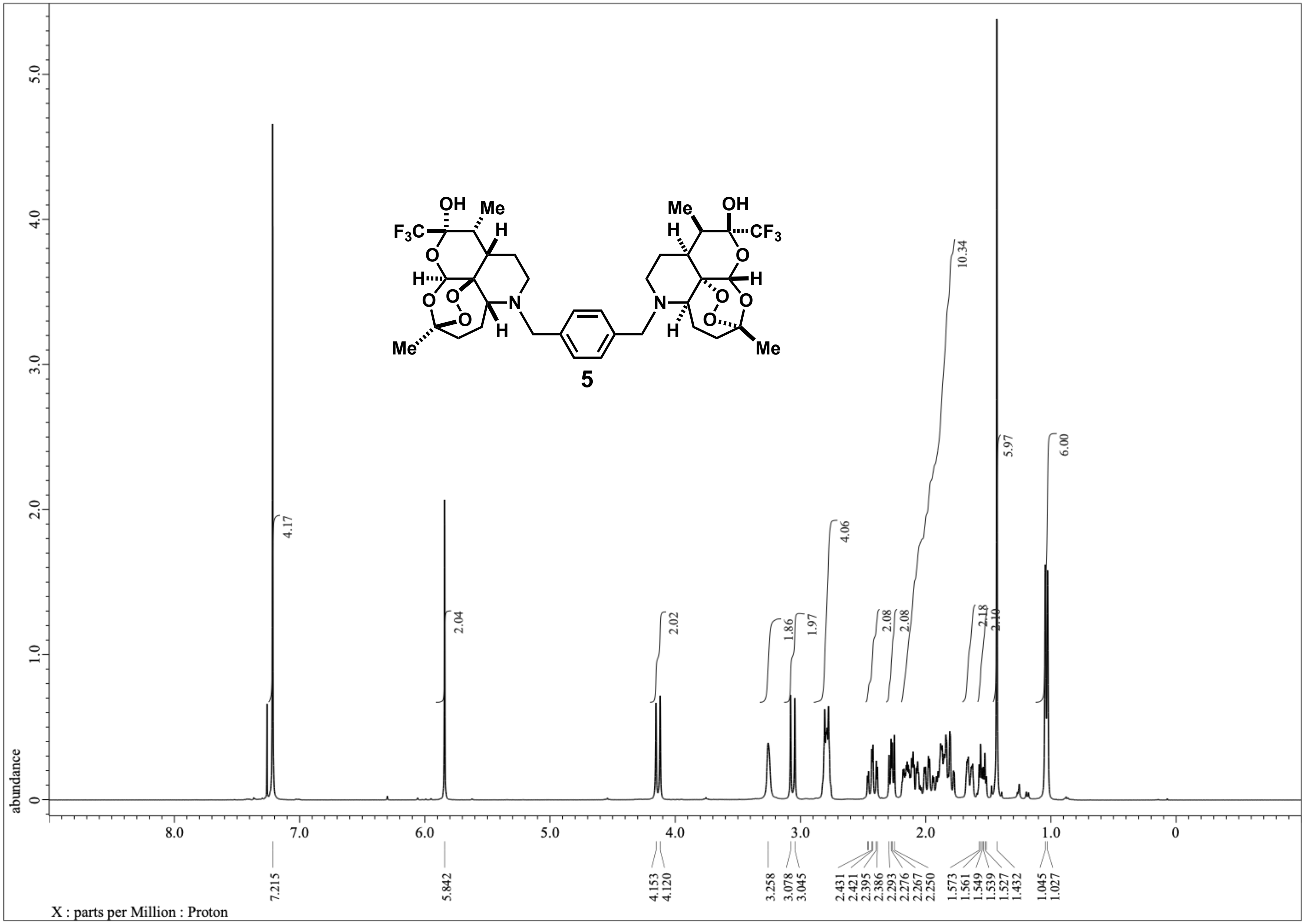

Figure S23. ^{1}H NMR (400 MHz, $CDCl_3$) spectrum of compound **5**

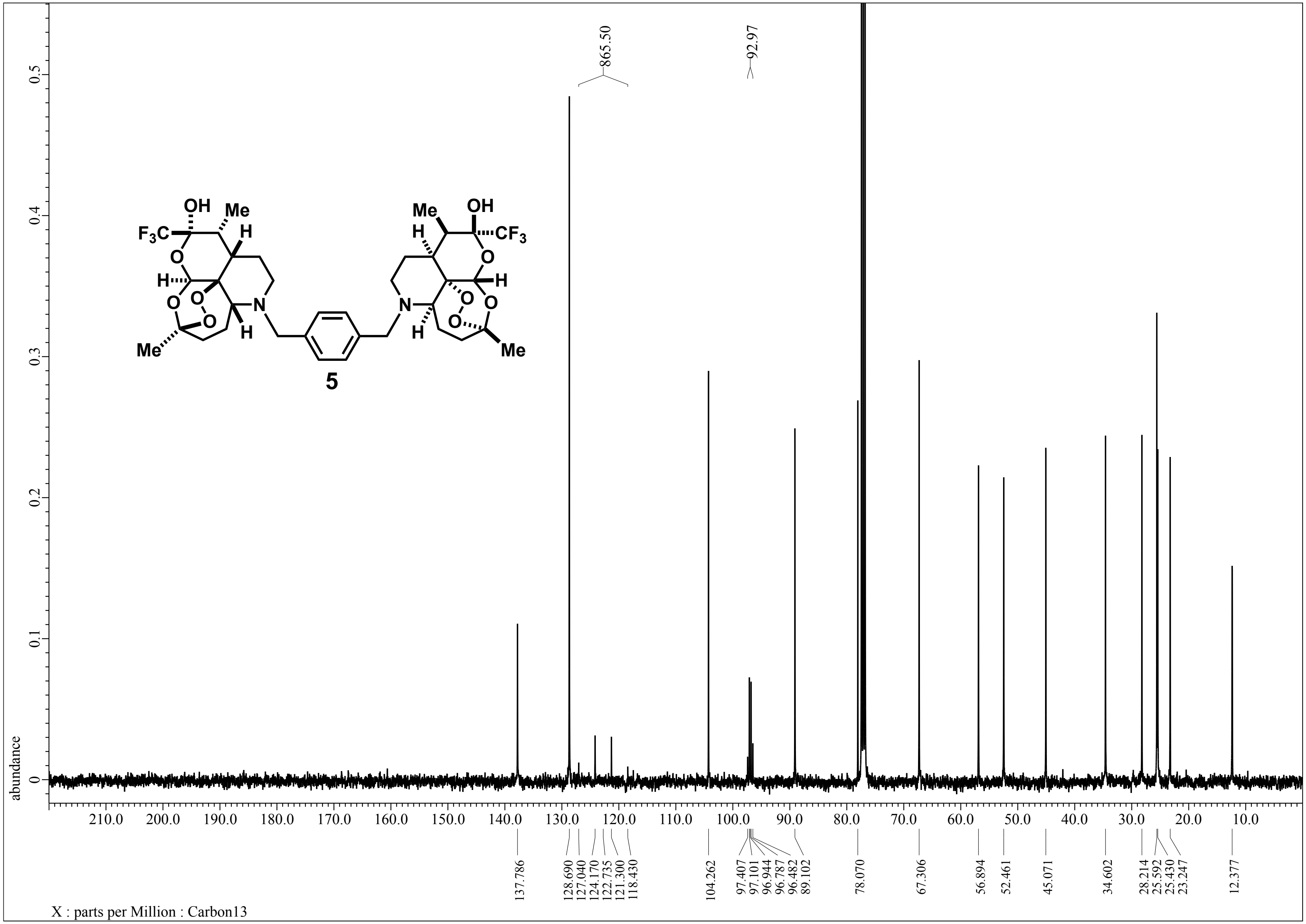

Figure S24. ^{13}C NMR (101 MHz, $CDCl_3$) spectrum of compound **5**

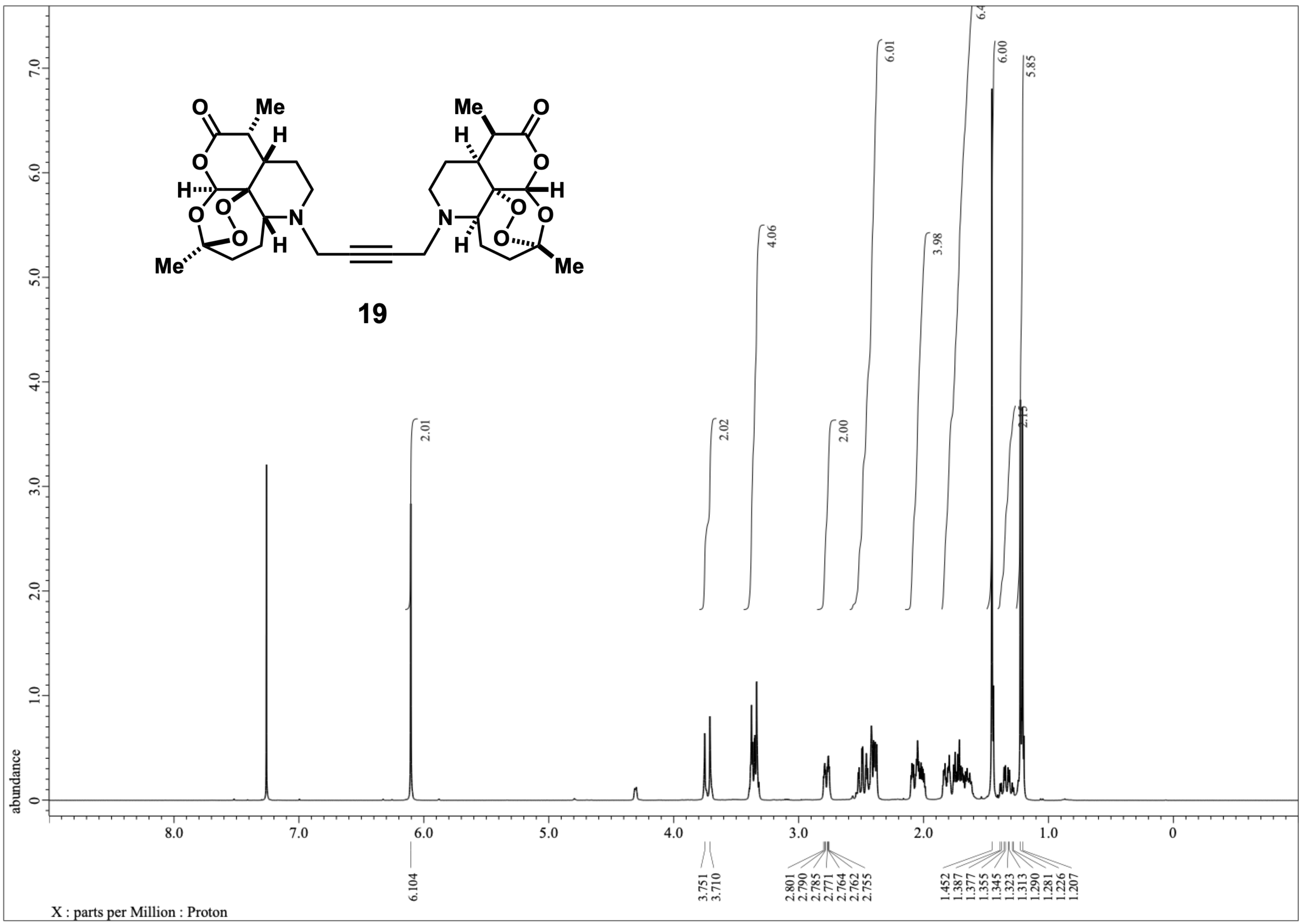

Figure S25. ^{1}H NMR (400 MHz, $CDCl_3$) spectrum of compound **19**

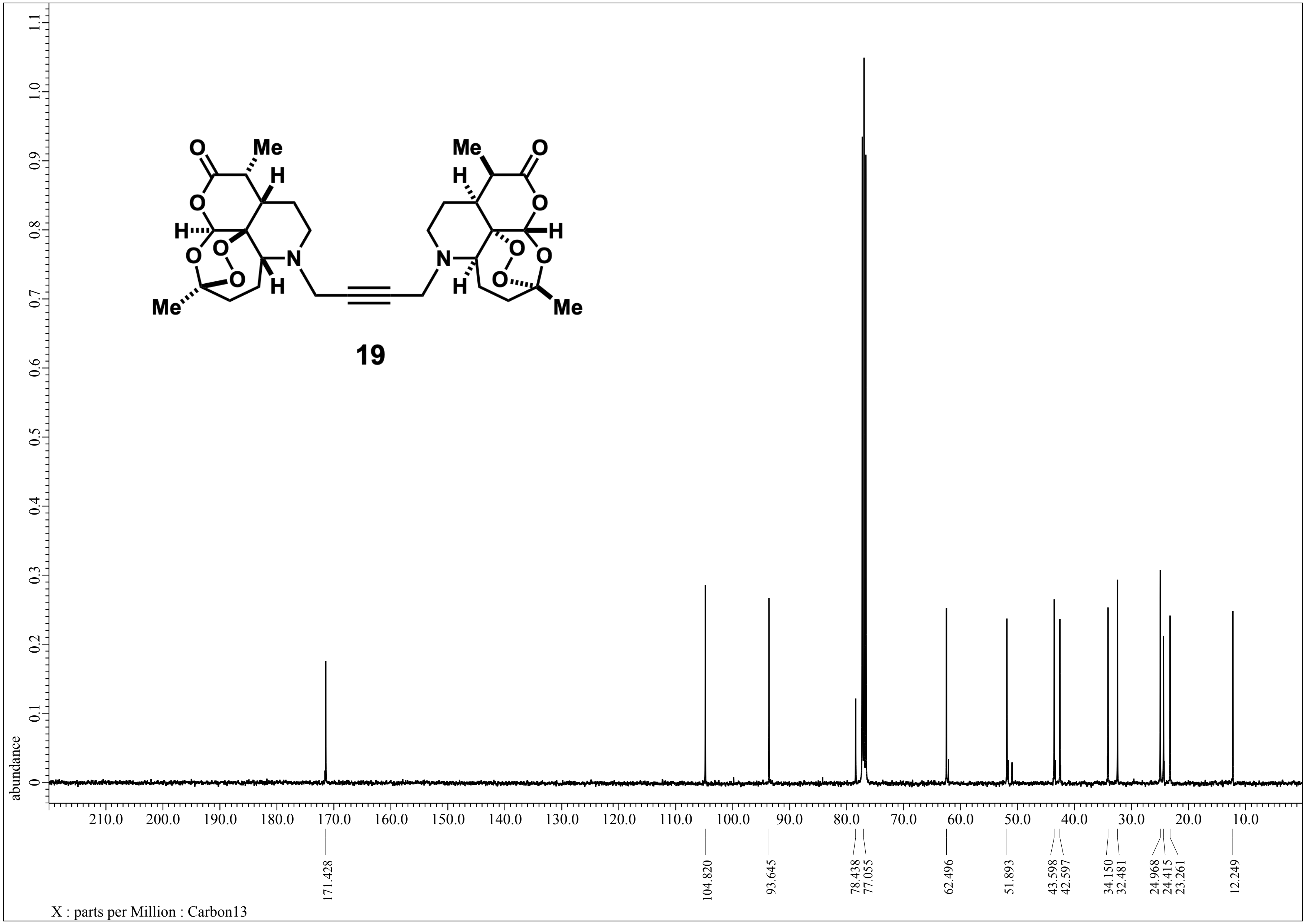

Figure S26. ^{13}C NMR (101 MHz, $CDCl_3$) spectrum of compound **19**

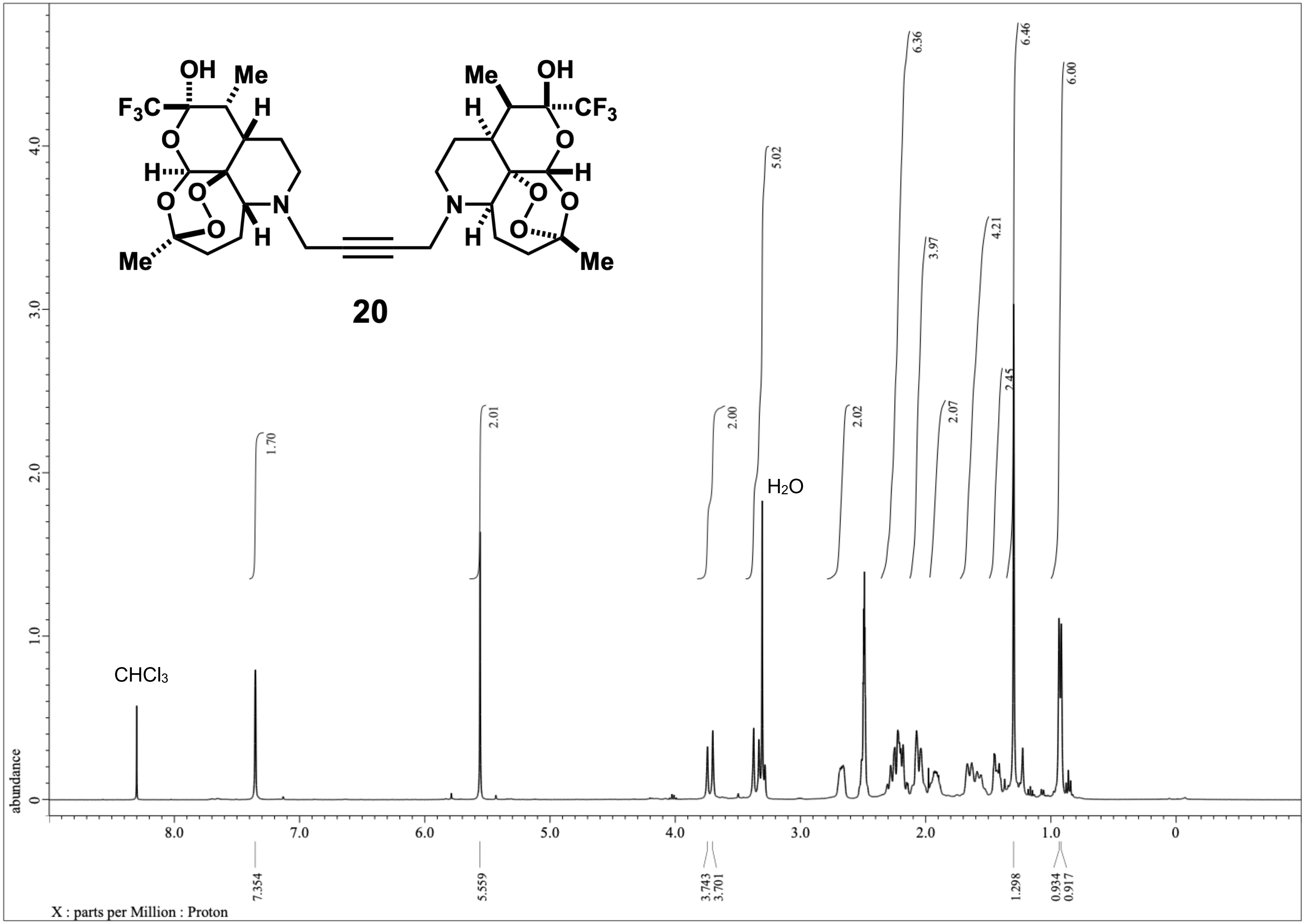

Figure S27. ^{1}H NMR (400 MHz, DMSO-d_6) spectrum of compound **20**

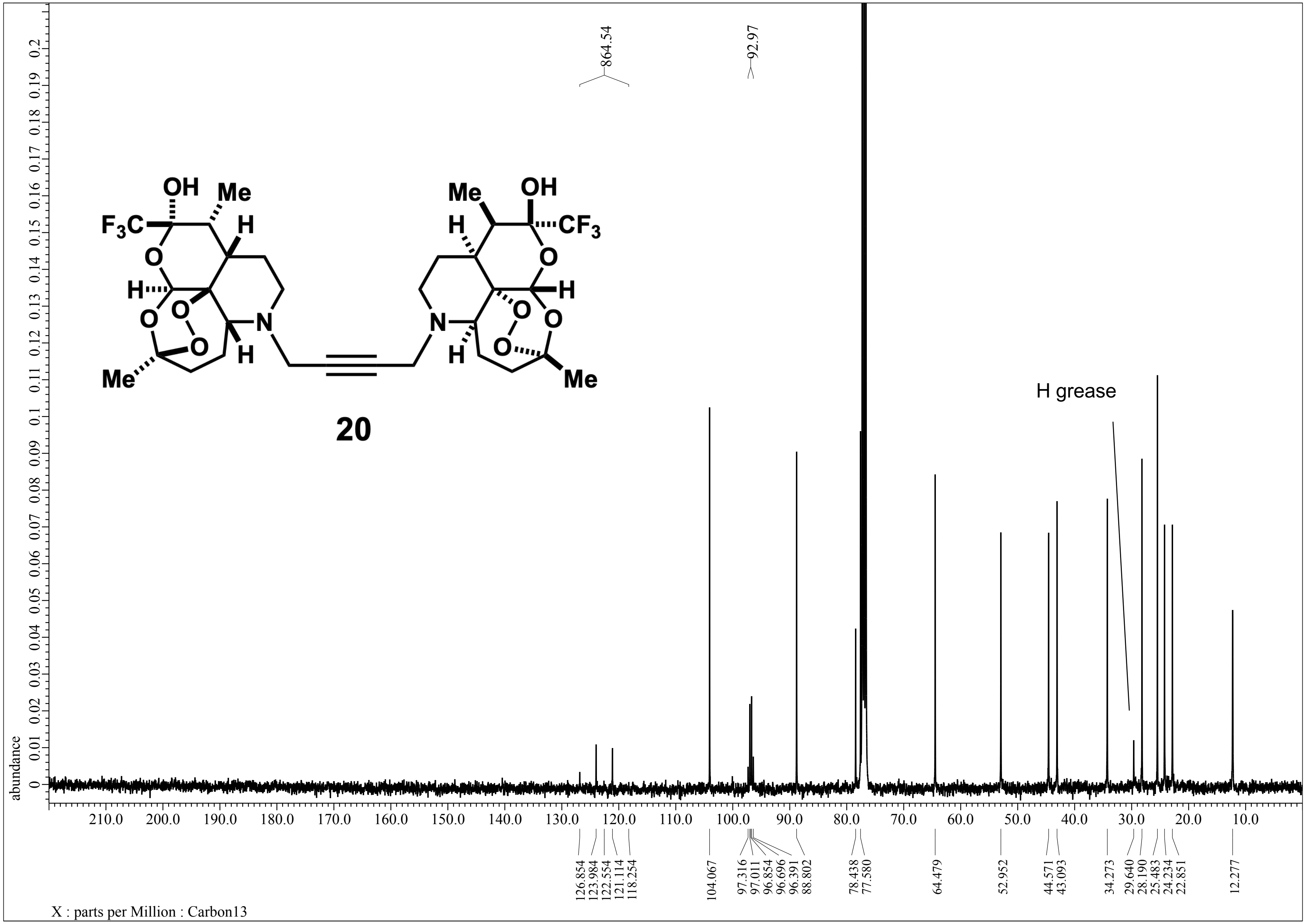

Figure S28. ^{13}C NMR spectra (101 MHz, $CDCl_3$) of compound **20**
